# Design, Synthesis and Biological Evaluation of Arylpyridin-2-yl Guanidine Derivatives and Cyclic Mimetics as Novel MSK1 Inhibitors. An Application in an Asthma Model

**DOI:** 10.3390/molecules26020391

**Published:** 2021-01-13

**Authors:** Maud Bollenbach, Simona Nemska, Patrick Wagner, Guillaume Camelin, François Daubeuf, Adeline Obrecht, Pascal Villa, Didier Rognan, Frédéric Bihel, Jean-Jacques Bourguignon, Martine Schmitt, Nelly Frossard

**Affiliations:** 1 Laboratoire d’Innovation Thérapeutique, Institut du Médicament (IMS), UMR 7200, CNRS, Faculté de Pharmacie, Université de Strasbourg, F-67400 Illkirch, France; maudbollenbach@gmail.com (M.B.); simona.nemska@humanitasresearch.it (S.N.); pwagner@unistra.fr (P.W.); camelin.guillaume@gmail.com (G.C.); daubeuf@unistra.fr (F.D.); drognan@unistra.fr (D.R.); fbihel@unistra.fr (F.B.); jjb@unistra.fr (J.-J.B.); 2 PCBIS Plate-forme de Chimie Biologique Intégrative de Strasbourg, Institut du Médicament (IMS), UMS 3286, CNRS, Université de Strasbourg, F-67412 Illkirch, France; aobrecht@unistra.fr (A.O.); pvilla@unistra.fr (P.V.)

**Keywords:** Pyridine-2-yl guanidine, MSK1, kinase inhibitors, inflammation, asthma

## Abstract

Mitogen- and Stress-Activated Kinase 1 (MSK1) is a nuclear kinase, taking part in the activation pathway of the pro-inflammatory transcription factor NF-kB and is demonstrating a therapeutic target potential in inflammatory diseases such as asthma, psoriasis and atherosclerosis. To date, few MSK1 inhibitors were reported. In order to identify new MSK1 inhibitors, a screening of a library of low molecular weight compounds was performed, and the results highlighted the 6-phenylpyridin-2-yl guanidine (compound **1a**, IC_50_~18 µM) as a starting hit for structure-activity relationship study. Derivatives, homologues and rigid mimetics of **1a** were designed, and all synthesized compounds were evaluated for their inhibitory activity towards MSK1. Among them, the non-cytotoxic 2-aminobenzimidazole **49d** was the most potent at inhibiting significantly: (i) MSK1 activity, (ii) the release of IL-6 in inflammatory conditions in vitro (IC_50_~2 µM) and (iii) the inflammatory cell recruitment to the airways in a mouse model of asthma.

## 1. Introduction

**Figure 1:**
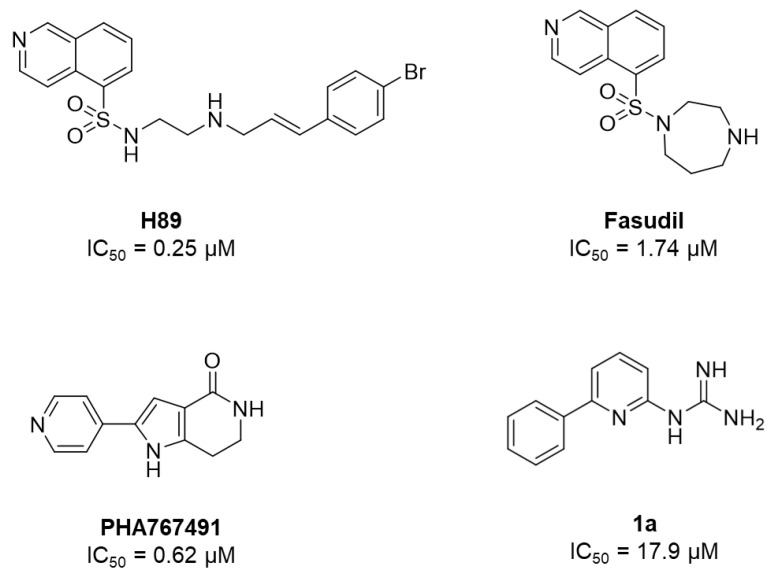
MSK1 inhibitors. Chemical structures and IC_50_ values of three of the most common MSK1 inhibitors, **H89**, **fasudil** and **PHA767491**, and of the compound **1a**, selected hit from an enzymatic screening.

Mitogen- and Stress-Activated Kinase 1 (MSK1) is a nuclear Ser/Thr kinase from the AGC kinase family and is activated in inflammatory conditions (IL-1β, TNFα) or upon stimulation by growth factors (EGF, TGF), mitogens or stress via phosphorylation by ERK1/2 and/or p38 MAPK [1]. Following these stimuli, MSK1 activates the phosphorylation of nuclear substrates including the transcription factors NF-kB, CREB and the histone H3 [2,3,4], thereby inducing the transcription of pro-inflammatory genes such as the cytokines IL-6, IL-11, IL-2 [1,2,3,5,6,7,8,9,10,11]. The inflammatory transcription factor NF-kB is phosphorylated by MSK1 on its p65 sub-unit Ser276, which allows the increased production of the pro-inflammatory cytokine IL-6 in pulmonary fibroblasts upon IL-1β stimulation [2]. MSK1 has been involved in various inflammatory diseases such as asthma, psoriasis, atherosclerosis [7,11,12,13,14,15], suggesting that MSK1 could be a potential therapeutic target for such inflammatory disorders. A few compounds have been reported as MSK1 inhibitors like **H89** [16], **fasudil** (HA-1077) [16] and **PHA767491** [17] (Figure 1).

**Figure 2:**
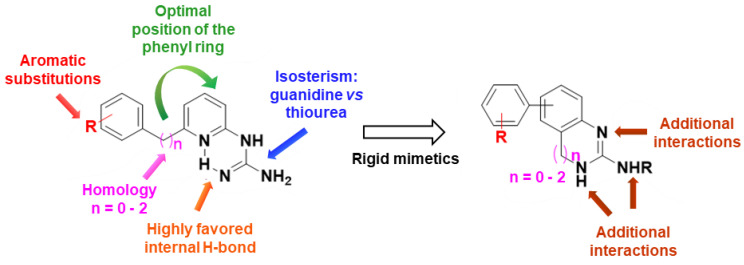
Pyridinyl-guanidine template (hit compound 1a) and strategy for SAR.

Regarding specificity for kinase inhibition, **H89** is a potent MSK1 inhibitor, also inhibiting PKA, S6K1 and ROCKII [16]. Fasudil inhibits MSK1 as well as MLCK, Rho kinases and PRK and was used for treatment of cerebral vasospasms [18,19]. **PHA767491** is MSK1 inhibitor with potent CDC7, CDK9 and MK2 inhibitory activity [17]. Other compounds such as **SB 747651A** [12] and **Ro318220** [16] were also described as potent and selective MSK1 inhibitors in vitro, but were less used in in vivo studies [12]. In this direction, there was a need for the discovery of innovative, safe and active in vivo MSK1 inhibitors with a therapeutic and/or pharmacological tool potential. In order to achieve this purpose, a screening of approximately 6800 molecules with large chemical diversity has been performed using an enzymatic assay with a peptide substrate mimetic of the sequence surrounding the Ser276 of NF-kB [2]. For the reference inhibitors **H89**, **fasudil** and **PHA767491**, we obtained comparable IC_50_ values as reported previously (Figure 1). From our screening, we selected the 1-(6-phenylpyridin-2-yl)guanidine (**1a**, IC_50_ = 17.9 µM, Figure 1) as a promising starting hit with multiple options for structural modifications (Figure 2).

Our aim was to provide a deeply structure–activity relationship (SAR) analysis resulting from the: (i) evaluation of a potential beneficial effect of a phenyl ring at position 3–6 along the pyridine nucleus; (ii) introduction of chemical diversity by means of different types of substituents accounting for lipophilic, electronic and geometric parameters, and search for additional interactions, homology and isosterism; (iii) replacement of the pyridine nucleus by a bicyclic scaffold as a valuable rigid frame, able to mimic a highly favored internal H-bond interaction. For this purpose, different homologues were considered and aminodihydroquinazoline derivatives **37**, **43**, **60**, benzimidazoles **49**, **50**, **74**, **75** and the dihydro 1,3-benzodiazepine **57** were synthesized, as illustrated in Figure 2.

## 2. Results

### 2.1. Screening and Hit Selection

**Figure 3:**
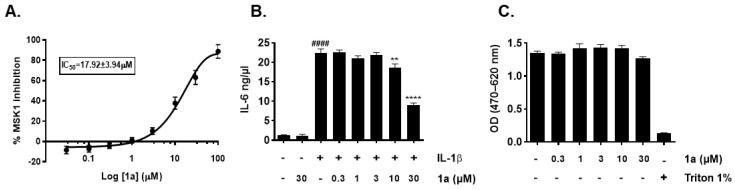
Enzymatic and in vitro characterization of the hit compound **1a**. (**A**) Enzymatic inhibition of MSK1 (Kinase-Glo^®^, Promega). Bullets are means and bars are SD (*n* = 4). (**B**) Inhibition IL-6 production (ELISA) after in vitro inflammatory stimulation of human primary pulmonary fibroblasts by IL-1β. Blocks are means and bars are SEM (*n* = 4). ** *p* < 0.01, **** *p* < 0.0001 compared to the control IL-1β group. ^####^ *p* < 0.0001 compared to the non-stimulated control. (**C**) Cell toxicity evaluation in human pulmonary fibroblasts (WST1 assay) after 24 h of incubation. Blocks are means and bars are SEM (*n* = 4).

We optimized a miniaturized enzymatic screening assay using a substrate mimetic of the p65 NF-κB subunit, surrounding the Ser276, which is the natural phosphorylation site of MSK1. We used a purified active human enzyme for the tests. We screened compounds from: (i) the Strasbourg University chemical library, which consists of around 4800 new chemical entities (NCE) resulting from specific organic chemistry methodologies and medicinal chemistry programs, including mainly heterocyclic derivatives and natural compounds from plants; (ii) the Prestwick chemical library (http://www.prestwickchemical.com; 1200 compounds); and (iii) The National French Essential Library (http://chimiotheque-nationale.enscm.fr; 640 compounds representative of different chemical families). Altogether around 6800 molecules were screened. The robustness of the assay was assessed by using the Z’ factor [20], which is usually used in high-throughput screening (HTS). We obtained a Z’ > 0.5 which was a guarantor of the quality of our assay. Among the active compounds identified, we selected one molecule of interest, compound **1a** with an IC_50_ value for MSK1 of 17.9 µM, which was active and non-toxic in a cell-based assay, as a leading hit to perform the SAR analysis (Figure 3).

### 2.2. Synthesis of the Compounds

**Scheme 1:**
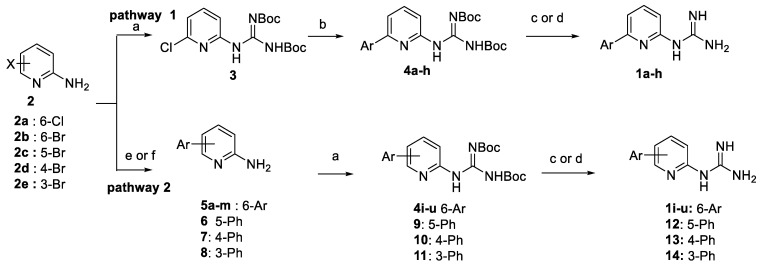
General preparation of 2-guanidinopyridines 1, 12–14. *Reaction conditions*: (a) *N*,*N*′-diBoc-*S*-methylisothiourea, HgCl_2_, NEt_3_, DCM, rt, 16 h, 29–98%; (b) ArB(OH_)2_, Pd(OAc)_2_, X-Phos, Cs_2_CO_3_, *n*BuOH:H_2_O (4:1), 50 °C, 16 h, 42–94%; (c) TFA:DCM (1:1), rt, 1 h, 33–100%; (d) HCl, Et_2_O, rt, 5 h, 42–80%. (e) ArB(OH)_2_, Pd(PPh_3_)_4_, Na_2_CO_3_, Tol:EtOH:H_2_O (5:1:1), 120 °C, 2 h–16 h, 59–93%; (f) ArB(OH)_2_, Pd(OAc)_2_, S-Phos, K_2_CO_3_, MeCN:H_2_O (2:1), 105 °C, 3 h, 80–82%.

Chemical methodologies used for the preparation of target 2-guanidinopyridine derivatives **1**, **12**–**14** are illustrated in Scheme 1. Accelerating drug optimization has a great importance in drug design and needs development of convergent strategies. Considering this, we focused our attention on the use of diprotected-6-chloropyridine guanidine **3** as a key precursor for rapid introduction of various aromatics or heteroaromatics in position **6**, using the Suzuki-Miyaura reaction (Pathway 1, Scheme 1) [21]. Starting from the commercially available 6-chloro 2-aminopyridine (**2a**), a guanylation reaction using di-Boc-SMe-isothiourea in presence of HgCl_2_ led to compound **3** in 86% yield. In the presence of Pd(OAc)_2_ and X-Phos at 50 °C [22], different aryl boronic acids (see Table 1, Table 2 and Table 3), carrying either electron donating or withdrawing substituents enabled the formation of 6-arylpyridineguanidines **4a**–**h**.

These mild conditions have the advantage of avoiding the deprotection of the Boc group during the cross-coupling reaction, but yields depend on the steric hindrance of the starting phenylboronic acid. Indeed, starting from a bulky 2-chloro-3-trifluoromethylphenylboronic acid, the desired product **1i** was isolated in poor yield (<30%, see the Materials and Methods section). To overcome this poor reactivity, a second synthetic route has been optimized (pathway 2) in which the Suzuki-Miyaura reaction using Pd(PPh_3_)_4_ was performed first, followed by the guanylation reaction. Acidic treatment of **4** allowed to isolate the desired product 1 as salts. Starting from 5-bromo aminopyridine (**2c**), the same conditions (pathway 2) were successfully applied for the preparation of 5-phenyl pyridinylguanidine (**12**). However, starting from a more basic halogeno-aminopyridines **2d** and **2e**, the cross-coupling reactions needed the use of Pd(OAc)_2_ and S-Phos to proceed in good yields, as previously reported by our group [21].

**Scheme 2:**
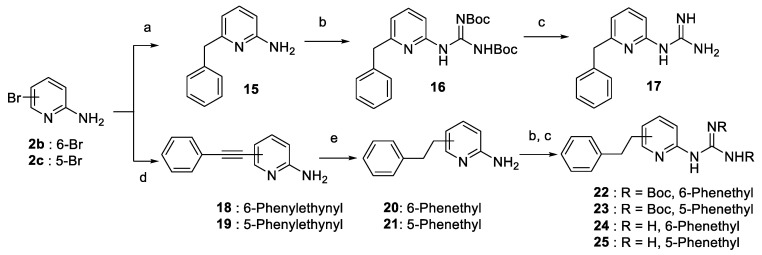
Synthesis of 6-benzyl and 5 (6)-phenethyl 2-guanidinopyridines 17 and 24–25*. Reaction conditions*: (a) B-Benzyl-9-BBN, Pd(OAc)_2_, S-Phos, K_3_PO_4_, THF, 100 °C, 1.5 h, 99%, (b) *N*,*N*′-diBoc-*S*-methylisothiourea, HgCl_2_, NEt_3_, DCM, rt, 16 h, 80–88%, (c) TFA:DCM (1:1), rt, 1 h, 43–56%. (d) phenylacetylene, PdCl_2_(PPh_3_)_2_, CuI, NEt_3_, MeCN, 100 °C, 3 h, 43–90%; (e) Pd/C, H_2_ (70 psi), rt, 24 h, 49–63%.

A catalyzed Pd(OAc)_2_/S-Phos Suzuki–Miyaura cross-coupling reaction between **2b** and B-Benzyl-9-BBN furnished the 6-Bn aminopyridine derivative **15** in a quantitative yield. Subsequent guanylation of **15** followed by deprotection of the Boc group, afforded **17** as described in Scheme 2. The phenethyl analogs **24** and **25** were prepared in four-steps. The key phenethyl intermediates **20** and **21** were synthesized starting from the corresponding bromo-2-aminopyridine **2b** or **2c** via a Sonogashira reaction, followed by a Pd/C catalytic hydrogenation. As previously described, a guanylation reaction followed by an acidic treatment allowed to isolate the desired compounds **24** and **25** [23].

**Scheme 3:**
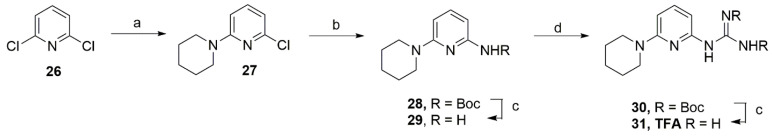
Synthesis of 2-guanidino-6-piperidinopyridine **31**. ^a^ *Reaction conditions*: (a) Piperidine, K_3_PO_4_, dioxane, 105 °C, 36 h, 81%; (b) NH_2_Boc, Pd(OAc)_2_, XantPhos, Cs_2_CO_3_, dioxane, 70 °C, 4 h, 83%; (c) TFA:DCM (1:1), rt, 2 h, 82–94%; (d) *N*,*N*′-diBoc-S-methylisothiourea, HgCl_2_, NEt_3_, DCM, rt, 16 h, 57%.

The piperidinyl-pyridine derivative **31** was prepared in five-steps sequence starting from the commercially available 2,6-dichloropyridine (**26**, Scheme 3). Two consecutive C-N bonds reactions (nucleophilic aromatic substitution (SNAr) with piperidine and Buchwald-Hartwig cross-coupling reaction with *tert*-butylcarbamate using XantPhos as ligand were performed, and yielded the key intermediate **28**. Removal of the *N*-Boc protecting group and introduction of the guanidine moiety with previously established guanylation procedure afforded the trifluoroacetic salt of the target compound **31** (Table 3) after acidic treatment. 

**Scheme 4:**
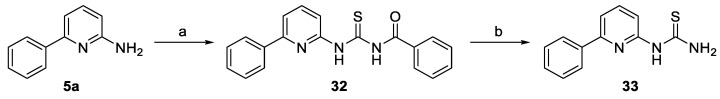
Synthesis of 1(6-phenylpyridin-2-yl)thiourea **33***. Reaction conditions*: (a) PhCON=C=S, THF, 70 °C, 14 h, 97%; (b) NaOH, EtOH:H_2_O (1:1), 80 °C, 1.5 h, 36%.

Treatment of 2-amino-6-phenylpyridine **5a** with benzoylisothiocyanate afforded the corresponding N-heteroaryl N-benzoylthiourea (**32**), which was smoothly hydrolyzed under basic conditions, to yield the desired thiourea **33** (Scheme 4).

**Scheme 5:**
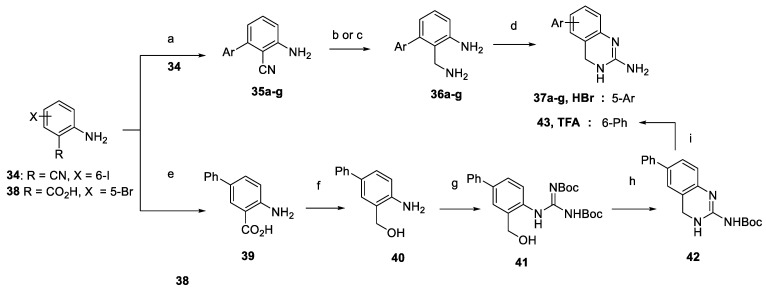
Synthesis of 5 and 6-aryldihydroquinazolines **37a**–**g** and **43**. *Reaction conditions*: (a) ArB(OH)_2_, Pd(PPh_3_)_4_, Na_2_CO_3_, Tol:EtOH:H_2_O (5:1:1), 120 °C, 2 h, 80–92%; (b) BH_3_.SMe_2_, THF, 75 °C, 2 h, 89%; (c) Ar = 2-OMePh, LiAlH_4_, AlCl_3_, THF, rt, 22 h, 70%; (d) BrCN, Toluene, 110 °C, 4 h, 49–79%; (e) PhB(OH)_2_, Pd(OAc)_2_, Na_2_CO_3_, H_2_O, 95 °C, 16 h; (f) LiAlH_4_, THF, rt, 12 h, 95% over 2 steps; (g) *N*,*N*′-diBoc-S-methylisothiourea, HgCl_2_, NEt_3_, DCM, rt, 16 h, 70%; (h) SOCl_2_, DCM, 50 °C, 2.5 h; (i) (TFA:DCM (1:1), rt, 1 h, 36% over 2 steps.

5-Aryl-dihydroquinazolines **37a**–**g** were prepared via a Pd(PPh_3_)_4_ catalyzed Suzuki-Miyaura reaction with the respective boronic acids, starting from commercially available 2-amino-6-iodo-benzonitrile (**34**, Scheme 5). Reduction of the cyano derivatives **35a**–**f** with BH_3_.SMe_2_ yielded the corresponding 1,2 diaminoarenes **36a**–**f** in good yields. Starting from **35g** (Ar = 2-OMePh), these reaction conditions were tedious and purification was time consuming. However, when the reaction was carried out in presence of a more powerful reducing agent (e.g., LiAlH_4_ in the presence of AlCl_3_) [24] compound **36g** was isolated in 70% yield after purification. Finally, intermediates **36a**–**g** were subjected to an intramolecular cyclisation using BrCN that efficiently allowed the formation of **37a**–**g**. Starting from 5-bromoanthranilic acid (**38**), a ligand-free Suzuki reaction was performed directly with Pd(OAc)_2_ in water. Reduction of the resulting carboxylic acid **39** with LiAlH_4_ yielded the key 2-aminophenylmethanol intermediate **40**. A guanylation reaction followed by an intramolecular cyclisation using SOCl_2_, afforded the monoprotected dihydroquinazoline **42** as described previously [22]. A final acidic treatment led to the desired 6-phenyldihydroquinazoline (**43)** as a salt.

**Scheme 6:**
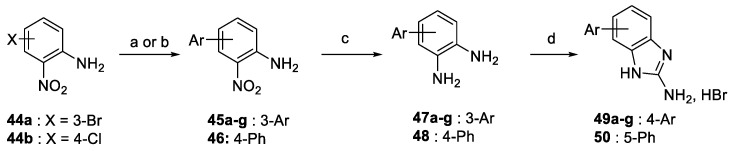
Synthesis of 4(5)-arylbenzimidazoles **49a**–**g** and **50**. *Reaction conditions*: (a) ArB(OH)_2_, Pd(OAc)_2_, S-Phos, K_2_CO_3_, MeCN:H_2_O (2:1), 105 °C, 3 h, 46–93%; (b) ArB(OH)_2_, Pd(PPh_3_)_4_, Na_2_CO_3_, Tol:EtOH:H_2_O (5:1:1), 120 °C, 2 h, 78–81%; (c) Sn, HCl, EtOH, 0–80 °C, 1 h, 68–100%; (d) BrCN, toluene, 110 °C, 4 h, 31–79%.

The lower homologues—the 2-aminobenzimidazoles **49** and **50**—were prepared from 3(4)-halogeno-2-nitroaniline **44** through a Suzuki-Miyaura/tin-HCl reduction/cyanogen bromide sequence following the procedure described in Scheme 6 [21]. Two optimized conditions (a or b) were developed for the Suzuki-Miyaura cross-coupling reaction. The catalytic Pd(OAc)_2_/S-Phos system was successfully employed for various boronic acids (see the Materials and Methods section). Unfortunately, under the same conditions, the reaction using 4-chlorophenylboronic acid was unsuccessful and the target compound **45c** was obtained in only 17% yield. In addition to **45c**, we observed the formation of a di-adduct resulting from two consecutive cross-coupling reactions (C-N arylation prior to C-C arylation, see Materials and Methods section). Instead, the use of standard Pd(PPh_3_)_4_ proceeded in good yield and **45c** was isolated in 78% yield after purification.

**Scheme 7:**
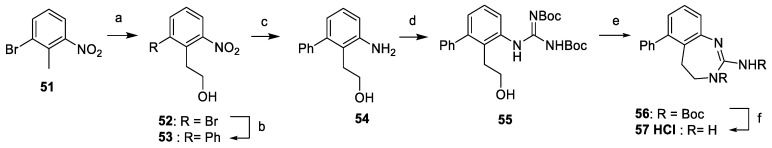
Synthesis of 6-phenyldihydrobenzodiazepine **57**. *Reaction conditions*: (a) CH_2_O, Triton B, DMSO, 90 °C, 16 h, 37%; (b) PhB(OH)_2_, Pd(OAc)_2,_ TBAB, K_2_CO_3_, H_2_O, 70 °C, 3 h, 91%; (c) Pd/C, H_2_ (60 psi), MeOH, rt, 3 days, quant.; (d) *N*,*N*′-diBoc-S-methylisothiourea, HgCl_2_, NEt_3_, DCM, rt, 16 h, 98%; (e) DIAD (2.0 equiv.), PPh_3_ (2.0 equiv.), THF, rt, 1.5 h; (f) HCl in dioxane, rt, 12 h, 49%.

Finally, the superior homologue of **37a**, the 6-phenyl-2-aminodihydrobenzodiazepine (**57**) was prepared in six steps according to Scheme 7. Nucleophilic substitution reaction of the commercially available 2-bromo-6-nitrotoluene (**51**) with paraformaldehyde and Triton B in DMSO, afforded the target precursor **52** in 77% yield. The bromoaryl derivative **52** was then subjected to a ligand-free Suzuki-Miyaura cross-coupling reaction in water, with TBAB as a phase transfer catalyst [25]. After a Pd/C catalytic hydrogenation of the nitro derivative **53**, a guanylation reaction was performed under conventional conditions. With the key intermediate **55** in hand, we performed an intramolecular Mitsunobu reaction using triphenylphosphine and diisopropyl azodicarboxylate (DIAD) in THF, followed by an acidic treatment and obtained the expected dihydrobenzodiazepine **57** as a salt.

**Scheme 8:**
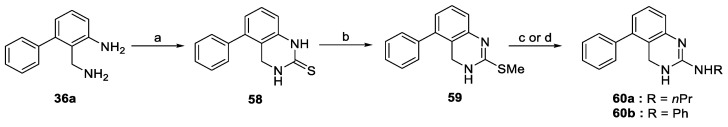
Preparation of N-substituted quinazolines **60**. *Reaction conditions*: (a) CSCl_2_, NEt_3_, Et_2_O, −78 °C to rt, 10 h, 72%; (b) MeI, acetone, rt, 12 h, 90%, (c) for R = nPr: propylamine, 110 °C, 0.5 h, 31%; (d) for R = Ph: aniline, 160 °C, 3 h, 19%.

Preparation of N-substituted quinazolines **60** involved a key intermediate, the 2-methylthio quinazoline (**59**), according to Scheme 8. Thione **58** was prepared by condensation at −78 °C between the previously described o-aminobenzylamine (**36a**) and thiophosgene [26] and then was methylated with iodomethane to give **59**. Finally, **59** reacted with aliphatic or aromatic amines under microwaves irradiation to afford **60**.

**Scheme 9:**
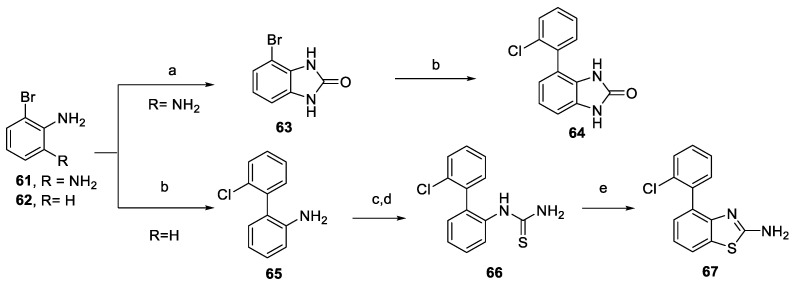
Access to 4-aryl dihydrobenzoimidazolone **64** and 4-aryl-2 aminobenzothiazole **67**. *Reaction conditions*: (a) CDI, THF, 50 °C, 18 h, 71%; (b) Pd(PPh_3_)4, Na_2_CO_3_, Tol:EtOH:H_2_O (5:1:1), 120 °C, 5 h–16 h, 61–87%; (c) PhCON=C=S, THF, reflux, 14 h, (d) NaOH, EtOH: H_2_O (1:1), 80 °C, 1.5 h, 94%; (e) Br_2_, DCM, 40 °C, 2 h, 42%.

4-Arylbenzimidazolone derivative **64** was obtained via a Pd(PPh_3_)_4_ catalyzed Suzuki-Miyaura reaction starting from the easily available 4-bromobenzimidazolone **63** [27]. Biphenyl-amine **65** was converted to the corresponding phenylthiourea **66** which undergoes intramolecular cyclisation in presence of bromine to yield substituted 2-aminobenzothiazole **67** (Scheme 9).

**Scheme 10:**
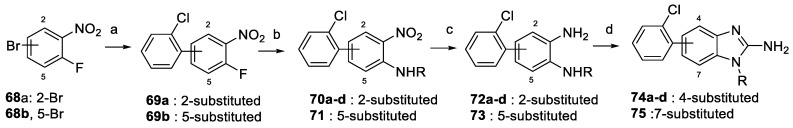
Access to N1-alkyl-2-amino benzimidazoles **74** and **75**. *Reaction conditions*: (a) Pd(PPh_3_)_4_, Na_2_CO_3_, Tol:EtOH:H_2_O (5:1:1), 110 °C, 2 h, 94%; (b) RNH_2_, DIEA, DMF, 25 °C, 54–74%; (c) Sn, HCl, EtOH, 2 h, reflux then NaOH, 30–53%; (d) BrCN, MeCN:MeOH (1:1), 80 °C, 3 h, 25–77%.

*N1*-alkyl-2-amino benzimidazoles **74** and **75** were prepared on the basis of molecular diversity concepts (Scheme 10). They were conveniently accessed via sequential nucleophilic aromatic substitution of an *o*-fluoronitobenzene (**68**) with various alkylamines (Table 4), reduction of the nitro group and final cyclisation of **72** and **73** with BrCN.

### 2.3. Evaluation of the New Compounds in an Enzymatic Assay for MSK1 Inhibition

**Figure 4:**
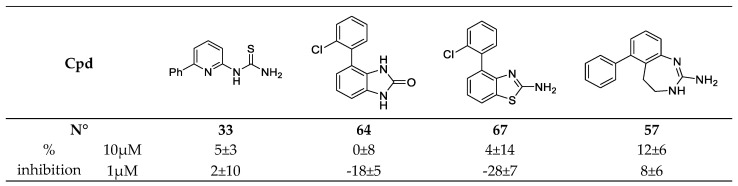
Structurally-related inactive compounds.

All synthesized compounds were evaluated for inhibitory activity of human synthetic MSK1 (14-438-K, Millipore) at two concentrations: 1 and 10 µM. Further the IC_50_ was measured for the best compounds (inhibition > 30% at 10 µM). All results are summarized in Table 1, Table 2, Table 3, Table 4 and Table 5 and on Figure 4. Our initial hit **1a** showed a 42% inhibitory activity on MSK1 at 10 µM with an IC_50_ = 17.9 ± 3.9 µM (Table 1, entry 5). The shift of the phenyl group from position 6 on the pyridine towards positions 3 (**14**, entry 1), 4 (**13**, entry 2) and 5 (**12a**, entry 3) led to inactive compounds at 10 µM. Introduction of an alkyl spacer in position 6 of compound **1a** led respectively to the benzyl (**17**, entry 6) and the phenethyl (**24**, entry 7) homologues, which were found inactive at 10 µM. These data clearly emphasized the critical role played by a phenyl ring directly linked at position 6 of the pyridine nucleus.

We next evaluated the substituent effect on the aromatic ring (Table 2). When compared with the parent compound **1a**, a fairly but significantly better activity was found with the 3-chloro derivative **1e** (entry 5 compared to entry 1). On the other hand, introduction of an electron-donating group (OMe) in the *meta* position (**1f**) and *para* position (**1d**) or an electron-withdrawing group (Cl and CF_3_) in the *para* position (**1b** and **1c**) led to inactive derivatives. However, a beneficial *ortho*-substitution was observed with both electro-donating (Table 2, entries 9 and 11) and electron-withdrawing groups (entries 7, 8, and 10). This gain of activity could be explained by the twist of the phenyl group, which optimized the interaction with the target.

This effect was optimal for a chlorine substitution (**1k**), with the improvement of one log affinity (IC_50_ = 0.6 ± 0.1 µM). As a beneficial 2-chloro substitution has been highlighted, we next turned our attention to a possible dichloro substitution, in order to find a synergistic effect. The disubstituted derivatives in position *ortho* and *meta*/*meta’* (**1o** and **1q**) have similar MSK1 inhibitory activities than our monochlorine compound **1k**, highlighting a steric tolerance. On the opposite, the *ortho*, *para*-dichloro derivative (**1p**) was less active (entry 13 compared to entries 12 and 14).

We also evaluated the possibility of exchanging aryl for heteroaryl rings (Table 3). In particular, the introduction of oxygen-containing heterocycles led to less active (**1g**) or totally inactive (**1h**) compounds. On the other hand, while the presence of 4-pyridyl moiety (**1r**) was detrimental to the activity, a nice beneficial effect was found with the 3-pyridyl derivative **1s** (IC_50_ = 5.8 ± 0.6 µM). Unfortunately, subsequent addition on position 2 of a chlorine atom (**1t**) did not improve, or a methoxy group (**1u**) abolished the inhibitory activity. Finally, introduction of a lipophilic piperidinyl moiety on position 6 (**31**) led to a totally inactive compound. This result supports the existence of specific π-π interactions of the phenyl group with the target. Subsequently, replacement of the guanidine moiety in **1a** by its isosteric thiourea **33** (Figure 4) yielded an inactive compound. The results clearly emphasize the critical role played by the entire guanidine moiety. This hypothesis is further supported by the inactivity of the 4-arylbenzo-imidazolone (**64**) and 4-aryl-2-aminobenzothiazole (**67**, Figure 4).

Rozas et al. highlighted the existence of an intramolecular hydrogen bonding system (see Figure 2), between the pyridine N1 atom and the guanidinium protons for pyridin-2-yl guanidine derivatives [28]. In order to determine the active conformation of our parent compound 1a and to design innovative scaffolds, a rigidification of this hydrogen bond to form a covalent bond has been realized, yielding three types of bicycles: 2-aminodihydroquinazolines (**37**, Table 4), 2-aminobenzimidazoles (**49**, Table 5) and 2-aminodihydrobenzodiazepine (**57**, Figure 4). The inhibitory effect on MSK1 was proportional to the size of the bicycle. Compared to our starting hit **1a**, the lower homologue 2-aminobenzimidazole 49a was more potent with an IC_50_ = 3.6 ± 0.5 µM, 2-aminodihydroquinazoline **37a** presented comparable activity IC_50_ = 15.8 ± 3.2 µM, while dihydrobenzodiazepine **57** was inactive.

As the benzimidazole and dihydroquinazoline derivatives showed promising MSK1 inhibition, we investigated the impact of an aromatic substitution. In the dihydroquinazoline serie (Table 4), different substituents were introduced but none showed improved efficiency. Only, the presence of a chlorine atom in ortho position was tolerated (**37f**), but it did not improve the potency compared to **37a**. A steric hindrance around the exocyclic nitrogen (Table 4) has been shown with the inactivity of the N-propyl (**60a**) and N-phenyl (**60b**) derivatives.

A set of substituted aryl-2-aminobenzimidazoles were tested (**49a**–**g**, **50**, **74a** and **74b**, Table 5). A fairly but significantly better activity was found with the introduction of a chlorine atom in *ortho* position (**49d**, IC_50_ = 1.6 ± 0.1), but this *ortho* substitution effect was not as strong as the acyclic series. We next examined possible substitution at N-1 with different functionalized aliphatic groups in order to identify an additional point of interaction of the receptor–ligand complex (entries 8–11, 13, Table 5), unfortunately no compound showed improved efficiency.

### 2.4. In Vitro Evaluation of Compounds

**Figure 5:**
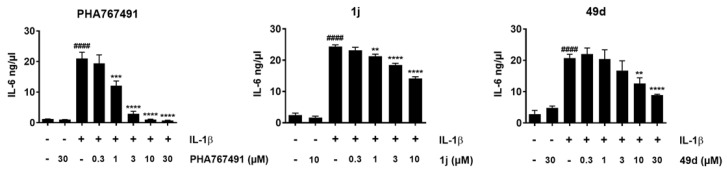
Inhibition of IL-6 production by reference compound **PHA767491**, **1j** (cytotoxic at 30 µM) and **49d** in inflammatory conditions. Blocks are means and bars are SEM values (*n* = 4). ** *p* < 0.01, *** *p* < 0.001, **** *p* < 0.0001 compared to the control IL-1β group. ^####^ *p* < 0.0001 compared to the non-stimulated control.

Monocyclic (**1a, 1j** and **1q**) and bicyclic **(37a**, **37f**, **49a** and **49d**) compounds exhibiting the best IC_50_ values for MSK1 inhibition, were selected for further in vitro analysis. In particular, we evaluated the inhibition of IL-6 production by the selected compounds cultured in human lung fibroblasts in inflammatory conditions using as positive control the three commonly known MSK1 inhibitors (PHA767491, H89 and fasudil). A first evaluation of the cell viability in presence of the compounds at concentrations up to 30 µM used the WST-1 assay, and strongly eliminated the cytotoxic compounds (cutoff cell viability < 75% at 10 µM). All results are summarized in Table 6. PHA767491 was the most effective MSK1 inhibitor in this assay with an IC_50_ = 1.0 ± 0.1 µM. Pyridine guanidine derivatives were tested but were found fairly active in comparison to PHA767491 (**1a**, IC_50_ = 16.3 ± 6.1, Figure 5) or cytotoxic (**1j** and **1q**). 2-Aminodihydroquinazolines were also evaluated but again were found to be inactive (**37a**, IC_50_ > 30 µM) or cytotoxic at 30 µM (**37f**). However, the 2-aminobenzimidazole series was less cytotoxic. Among them, the 2-chloro derivative **49d** was particularly interesting and presented the same potency for IL-6 inhibition (IC_50_ = 13.9 ± 9.7 µM) as fasudil (IC_50_ = 8.5 ± 5.1 µM) and H89 (IC_50_ = 10.5 ± 5.1 µM). Finally, we selected **49d** for further investigation in vivo in our murine asthma model.

### 2.5. In Vivo Evaluation of Compounds

Considering the inflammatory pathways in which MSK1 is implicated, we chose a mouse model of allergic asthma to evaluate the in vivo activity of the selected compounds. We already showed in the past the effect of **H89** in the asthma model [14]. As from all known MSK1 inhibitors, **PHA767491** showed the best inhibitory effect on MSK1 in the enzymatic assay and in vitro studies. 

**Figure 6:**
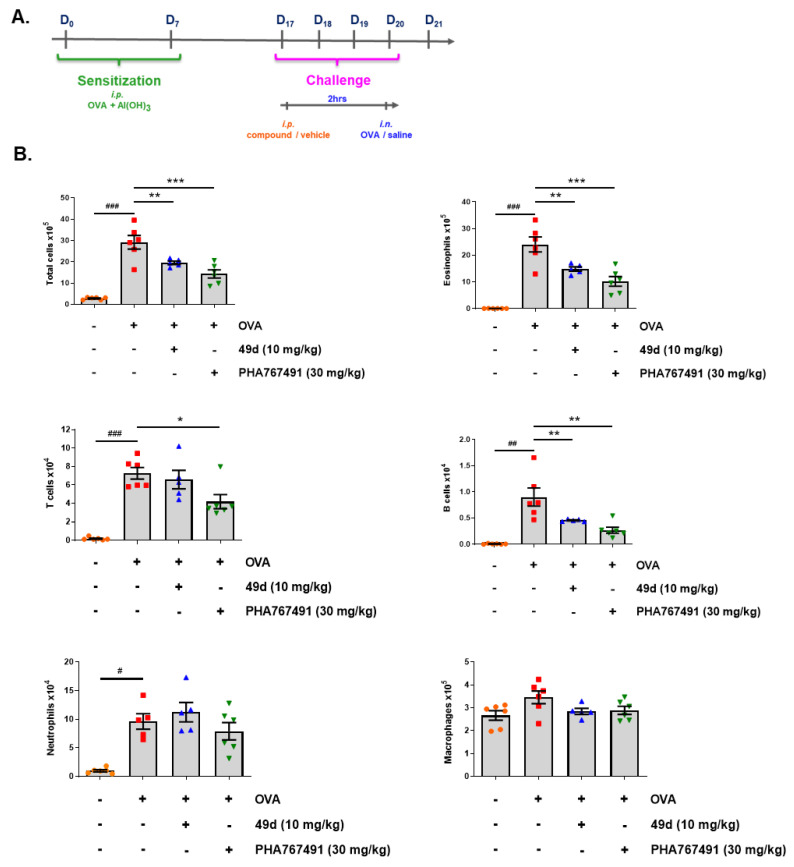
Effect of the new MSK1 inhibitor, **49d**, on airway inflammatory cells recruitment in an OVA-induced asthma model in Balb/c mice, compared to reference compound **PHA76491**. (**A**) Experimental design. Mice were sensitized by *i.p.* injections of OVA on days 0 and 7, and treated by *i.p.* with **49d** (10 mg/kg), PHA767491 (30 mg/kg) or the solvent (DMSO 5%, NaCl 0.9%), 2 h before each OVA or saline challenge (25 µl, *i.n.*) on days 17, 18, 19 and 20. (**B**) Absolute numbers of total cells, eosinophils, B cells, T cells, neutrophils and macrophages in BAL are shown. Blocks are means and bars are SEM values (*n* = 5–6). * *p* < 0.05, ** *p* < 0.01, *** *p* < 0.001 compared to OVA-solvent group. # *p* < 0.05, ## *p* < 0.01, ### *p* < 0.001 compared to control group**.**

We decided to use this compound in vivo together with our newly synthesized compound **49d**. We first checked for absence of compound toxicity by injecting **PHA767491** (30 mg/kg) or **49d** (10 mg/kg) by intraperitoneal route in mice by a unique injection to verify that no toxicity sign was observed for up to 48 h. Both compounds induced no sign of toxicity in Balb/C mice. We further tested a higher dose of **49d** (30 mg/Kg), that induced some signs of toxicity 48 h after a unique intraperitoneal injection (reduced motility, back curved with mid-closed eyes), so that the dose of 10 mg/mL was chosen for the in vivo experiment. We performed a 21-day model of eosinophilic airway inflammation induced by ovalbumin (OVA) in mice using these two compounds, **49d** and **PHA767491**, as a 4 day treatment (D7–D20, Figure 6). OVA sensitized/challenged mice displayed a significant infiltration of eosinophils, B and T cells and neutrophils in the broncho-alveolar lavage (BAL) fluid as compared to control mice (Figure 6). Treatment with **49d** significantly inhibited the total cell infiltration by 33 ± 3%, eosinophil infiltration by 39 ± 3% and B cell infiltration by 47 ± 1% (*p* < 0.05, Figure 6), clearly showing that this new MSK1 inhibitor is active in vivo as an anti-inflammatory agent. Treatment with **PHA767491** (30 mg/kg) has a significant and more pronounced effect, with decreased total cell infiltration by 51 ± 6%, eosinophil infiltration by 57 ± 7% (*p* < 0.001) and B cells infiltration by 68 ± 7% compared to control vehicle-treated mice (Figure 6). No significant effect was observed for any of the compounds on T-cell and neutrophil recruitment.

## 3. Discussion

In the last years, the research of new therapeutics for inflammatory disorders focused on intracellular targets such as kinases. The substrate phosphorylation by kinases leading to gene activation represents a major type of post-translational modification, allowing a multitude of possibilities for therapeutics [29,30]. Here we describe the synthesis of a new class of MSK1 inhibitors and the in vitro and in vivo characterization of the potent compound **49d**. Over the last years, the nuclear MSK1 kinase has been involved in various studies on inflammatory diseases such as asthma, psoriasis, atherosclerosis [7,11,12,13,14,15]. This kinase is directly activating the transcription factor NF-κB, thus representing a good therapeutic target with possible less side effects than upward kinases, in particular MAP kinases [2]. Asthma is a common inflammatory respiratory disease spread worldwide [31]. Our previous study in a mouse model of asthma showed that one of the available inhibitors of MSK1, **H89**, significantly inhibits the eosinophil recruitment in bronchoalveolar lavage [14]. It was already reported that this key cell of the allergic response during asthma, the eosinophil, is activated by cytokines (IL-5) and chemokines (CCL5, CCL11) via the activation of MSK1 [32]. In addition, MSK1 has also been implicated in the production of a major component of mucus, mucin MUC5AC, by airway epithelial cells in inflammatory conditions [33,34]. MSK1 shows also an increased activation and triggers the transcription of pro-inflammatory genes (IL-1β, Cox2) in macrophages activated by LPS [35,36,37]. Furthermore, the glucocorticoids that have proven anti-inflammatory effects in the airways are able to induce the export of MSK1 from the nucleus to the cytoplasm, and thus to inhibit the expression of MSK1-dependent pro-inflammatory genes [38,39]. Considering all these studies, MSK1 has been proposed as an interesting therapeutic target because of its pro-inflammatory activity and its nuclear localization, both suggesting a more specific downstream action of MSK1 inhibitors since preventing inhibition of the upstream p38 and ERK MAP kinases. The three common reference inhibitors we used here, **H89, fasudil** and **PHA767491**, were not developed as MSK1 inhibitors. Only compound **SB-747651A** was described as selective MSK1 inhibitor but was poorly used in vivo [12]. The only in vivo application was described by use of a unique administration in a microvascular neutrophil recruitment model in mice [11]. We aimed to develop an innovative MSK1 inhibitor, non-toxic and active in vivo, that could be used as pharmacological tool for further studies of the MSK1 implication in inflammatory disorders.

In this study, a primary screen of 6800 drug-like compounds using an enzymatic assay with a peptide substrate mimetic of the Ser276 NF-κB p65 subunit led to the selection of an initial hit, the 1-(6-phenylpyridin-2-yl)guanidine **1a**, as a new MSK1 inhibitor. Extensive structure-activity relationship study highlighted the benzimidazole as a privileged scaffold and led to the discovery of a 10-fold more potent MSK1 inhibitor (**49d**) compared to the parent compound, after rigidification of the intramolecular hydrogen bond leading to the 4-substituted aminobenzimidazole **49d**. Compound **49d** is a non-cytotoxic derivative active at inhibiting the production of the proinflammatory cytokine IL-6 in primary human pulmonary fibroblasts in culture. IL-6 production was selected as the read-out in our in vitro study since its expression is under the control of MSK1 activation during inflammation [2,4,40]. Furthermore, this new compound shows a potent in vivo effect in our asthma model by inhibiting the recruitment of eosinophils in the airways. We also show for the first time the in vivo effect in asthma of the reference MSK1 inhibitor **PHA767491**, with activity comparable to that of **H89** provided previously [14], thus consolidating the implication of the MSK1 kinase in asthma.

In conclusion, we here describe the synthesis and biological effect of a new potent and safe inhibitor of the nuclear kinase MSK1, that could be used as pharmacological tool to study the role of this kinase in animal models of inflammatory disorders. 

## 4. Materials and Methods

### 4.1. Chemical Synthesis

#### 4.1.1. General Information

All reactions were carried out under usual atmosphere unless otherwise stated. Chemicals and solvents were purchased from Sigma-Aldrich (St. Louis, MO, USA) and were used without further purification. Analytical TLC were performed using silica gel 60F254 plates (Merck, Kenilworth, NJ, USA) and plates were visualized by exposure to ultraviolet light. Compounds were purified on silica gel Merck 60 (particle size 0.040–0.063nm) or using Armen spot flash chromatography (normal phase column: Interchim 30 SHIP 25 g; reverse phase column: AIT 50 g C18). Yields refer to isolated compounds, estimated to be >95% pure as determined by ^1^H-NMR or HPLC. ^1^H-, ^19^F- and ^13^C-NMR spectra were recorded on an Avance spectrometer (Bruker, Billerica, MA, USA) operating at 400 or 500 MHz, 376 MHz and 100 or 125 MHz, respectively. All chemical shift values δ and coupling constants J are quoted in ppm and in Hz, respectively, multiplicity (s = singlet, d = doublet, t = triplet, q = quartet, quin = quintet, sex = sextet m = multiplet, br = broad). Melting points were realized using a B-540 Melting Point apparatus (Büchi, New Castle, DE, USA). Analytical RP-HPLC-MS was performed using a LC-MSD 1200SL (Agilent, Santa Clara, CA, USA) equipped with a Hypersilgold^®^ column (C18, 30 mm × 1 mm; 1.9 µm, Thermo, Waltham, Massachusetts, USA) using the following parameters: (1) Solvent system: A (acetonitrile) and B (0.05% TFA in H_2_O); (2) A linear gradient: t = 0 min, 98%B; t = 5 min, 5%B; t = 6 min, 5%B; t = 7 min, 98%B; t = 9 min, 98%B; (3) Flow rate of 0.3 mL/min; (4) Column temperature: 50 °C; (5) The ratio of products was determined by integration of spectra recorded at 210 nm or 254 nm; (6) Ionization mode: MM-ES+APCI. HPLC were performed using an UltiMate 300 system (Dionex, Sunnyvale, CA, USA) using the following parameters: Flow rate of 0.5 mL/min, column temperature: 30 °C, solvent system: A (MeOH) and B (0.05% of TFA in H_2_O), t = 0 min to 1 min: 50 to 60% of B then t = 1 min to t = 10 min: 60 to 100% of B and t = 10 min to t = 15 min: 100% of B. Microwave irradiation was performed with an Initiator EXP (external sensor type system, Biotage, Uppsala, Sweden).

The synthesis of compounds **1**, **17**, **37**, **49** will be reported here. Experimental details for all other analogues (compounds **31**, **33**, **60**, **64**, **67**, **74**, **75**) are available as Supporting Information. Compounds **1a**, **1b**, **1d**–**f**, **1j**, **1k**, **1n**, **10**, **11**, **12**–**14**, **37a**, **43**, **47**, **48**, **50 [21]**, **24–25 [23]**, and their respective intermediates were previously described in the literature and their analytical data were consistent with the previously reported characterization.

#### 4.1.2. General Method A: Preparation of Boc-Protected Guanidines Derivatives **3**, **4j**, **4l**, **4m**, **4o**–**u**, **16**

A solution of the appropriate 2-aminopyridine derivative (1.0 equiv.), *N*,*N*′-bis-(*tert*-butoxycarbonyl)-*S*-methylisothiourea (0.95 equiv.), triethylamine (4.4 equiv.) and mercury chloride (1.1 equiv.) were stirred at rt overnight in CH_2_Cl_2_ (3.2 mL/mmol). After completion of the reaction, the reaction mixture was filtered through a pad of Celite^®^ with CH_2_Cl_2_ as the washing solvent. The filtrate was concentrated under vacuum and purified by silica gel column chromatography, eluting with the appropriate hexane:EtOAc mixture.

∗ *2-[2,3-Di(tert-butoxycarbonyl)guanidino]-6-chloropyridine* (**3**). Following general method A and starting from 2-amino-6-chloropyridine (**2a**, 1.00 g, 7.78 mmol), **3** was obtained as a white solid (2.36 g, 6.37 mmol, 86%). ^1^H-NMR (400 MHz, CDCl_3_) *δ* ppm 1.53 (s, 18H), 7.03 (d, 1H, *J* = 7.9 Hz), 7.65 (t, 1H, *J* = 7.9 Hz), 8.36 (d, 1H, *J* = 7.9 Hz), 10.85 (s, 1H), 11.50 (s, 1H); ^13^C-NMR (101 MHz, CDCl_3_) *δ* ppm 28.0, 28.1, 80.2, 84.2, 114.2, 119.8, 140.6, 153.0.
∗ *2-[2,3-Di(tert-butoxycarbonyl)guanidino]-6-(2-fluorophenyl)pyridine* (**4j**). Following general method A and starting from **5b** (258 mg, 1.37 mmol), **compound 4j** was obtained as a white solid (495 mg, 1.15 mmol, 88%). ^1^H-NMR (400 MHz, CDCl_3_) *δ* ppm 1.51 (s, 9H), 1.54 (s, 9H), 7.16 (d, 1H, *J* = 7.4 Hz), 7.47–7.52 (m, 2H), 7.59 (t, 1H, *J* = 7.4 Hz), 7.73–7.79 (m, 2H), 8.41 (br s, 1H), 10.83 (s, 1H), 11.56 (s, 1H); ^13^C-NMR (101 MHz, CDCl_3_) *δ* ppm 28.0, 28.2, 79.7, 79.9, 115.1, 120.0, 122.7, 125.4, 126.3 (q, *J* = 5.9 Hz), 128.3, 128.2, 131.5, 131.6, 138.2, 139.5. 150.0, 153.3
∗ *2-[2,3-Di(tert-butoxycarbonyl)guanidino]-6-(2-methylphenyl)pyridine (***4l***)*. Following general method A and starting from **5d** (100 mg, 0.54 mmol), compound **4l** was obtained as a colorless oil (216 mg, 0.51 mmol, 98%). ^1^H-NMR (400 MHz, CDCl_3_) *δ* ppm 1.44 (s, 9H), 1.47 (s, 9H), 2.30 (s, 3H), 7.06 (d, 1H, *J* = 7.5 Hz), 7.17–7.22 (m, 3H), 7.31 (d, 1H, *J* = 7.5 Hz), 7.69 (t, 1H, *J* = 7.5 Hz), 8.25 (br s, 1H), 10.73 (s, 1H), 11.49 (s, 1H); ^13^C-NMR DEPT-135 (101 MHz, CDCl_3_) *δ* ppm 20.5, 28.0, 28.2, 120.1, 125.8, 128.3, 129.6, 130.7, 138.3.
∗ *2-[2,3-Di(tert-butoxycarbonyl)guanidino]-6-(2-trifluoromethylphenyl)pyridine* (**4m**). Following general method A and starting from **5e** (304 mg, 1.28 mmol), **4m** was obtained as a white solid (411 mg, 0.86 mmol, 71%). ^1^H-NMR (400 MHz, CDCl_3_) *δ* ppm 1.54 (s, 18H), 7.13 (dd, 1H, *J* = 8.7 Hz, *J* = 11.2 Hz), 7.24 (t, 1H, *J* = 7.4 Hz), 7.33–7.39 (m, 1H), 7.57 (d, 1H, *J* = 6.4 Hz), 7.78 (t, 1H, *J* = 7.9 Hz), 8.04 (br s, 1H), 8.37 (br s, 1H), 10.86 (s, 1H), 11.58 (s, 1H); ^13^C DEPT-135 NMR (101 MHz, CDCl_3_) *δ* ppm 28.1, 28.2, 116.1, 116.3, 120.6, 124.4 (q, *J* = 3.7 Hz), 130.4 (q, *J* = 8.1 Hz), 131.1, 138.6.
∗ *2-[2,3-Di(tert-butoxycarbonyl)guanidino]-6-(2,3-dichlorophenyl)pyridine* (**4o**). Following general method A and starting from **5g** (158 mg, 0.66 mmol), **4o** was obtained as a white solid (269 mg, 0.56 mmol, 89%). ^1^H-NMR (400 MHz, CDCl_3_) *δ* ppm 1.52 (s, 18H), 7.24–7.29 (m, 2H), 7.42 (dd, 1H, *J* = 1.6 Hz, *J* = 7.9 Hz), 7.49 (dd, 1H, *J* = 1.6 Hz, *J* = 7.9 Hz), 7.78 (t, 1H, *J* = 7.9 Hz), 8.39 (br s, 1H), 10.81 (s, 1H), 11.57 (s, 1H); ^13^C-NMR (101 MHz, CDCl_3_) *δ* ppm 28.1, 79.9, 80.1, 120.8, 127.2, 129.7, 130.3, 130.9, 133.6, 138.2, 140.9, 142.2, 146.5, 146.7, 153.5, 155.2.
∗ *2-[2,3-Di(tert-butoxycarbonyl)guanidino]-6-(2,4-dichlorophenyl)pyridine* (**4p**). Following general method A and starting from **5h** (182 mg, 0.76 mmol), **4p** was obtained as a colorless oil (330 mg, 0.69 mmol, 95%). ^1^H-NMR (400 MHz, CDCl_3_) *δ* ppm 1.53 (s, 18H), 7.32 (dd, 1H, *J* = 2.0 Hz, *J* = 8.4 Hz), 7.35–7.37 (m, 1H), 7.47 (d, 1H, *J* = 2.0 Hz), 7.54 (d, 1H, *J* = 8.4 Hz), 7.77 (t, 1H, *J* = 7.9 Hz), 8.38 (br s, 1H), 10.81 (s, 1H), 11.57 (s, 1H); ^13^C-NMR (101 MHz, CDCl_3_) *δ* ppm 28.2, 80.0, 80.1, 115.3, 120.9, 127.2, 128.6, 129.8, 132.6, 132.9, 134.8, 139.9, 143.4, 145.0, 149.6, 150.5, 153.2, 155.5.
∗ *2-[2,3-Di(tert-butoxycarbonyl)guanidino]-6-(2,5-dichlorophenyl)pyridine* (**4q**). Following general method A and starting from **5i** (143 mg, 0.60 mmol), **4q** was obtained as a yellow solid (264 mg, 0.55 mmol, 97%). ^1^H-NMR (400 MHz, CDCl_3_) *δ* ppm 1.54 (s, 18H), 7.28 (dd, 1H, *J* = 2.6 Hz, *J* = 8.5 Hz), 7.36–7.39 (m, 2H), 7.57 (s, 1H), 7.79 (t, 1H, *J* = 7.9 Hz), 8.39 (br s, 1H), 10.84 (s, 1H), 11.57 (s, 1H); ^13^C-NMR DEPT-135 (101 MHz, CDCl_3_) *δ* ppm 28.0, 28.2, 115.3, 120.9, 129.5, 130.5, 131.2, 131.5, 132.8, 138.2, 139.9.
∗ *2-[2,3-Di(tert-butoxycarbonyl)guanidino]-6-(4-pyridyl)pyridine* (**4r**). Following general method A and starting from **5j** (99 mg, 0.58 mmol), **4r** was obtained as a white solid (156 mg, 0.38 mmol, 68%). ^1^H-NMR (400 MHz, CDCl_3_) *δ* ppm 1.55 (s, 18H), 7.54 (d, 1H, *J* = 7.8 Hz), 7.83 (t, 1H, *J* = 7.8 Hz), 7.89 (br s, 2H), 8.48 (br s, 1H), 8.70 (d, 2H, *J* = 6.3 Hz), 10.94 (s, 1H), 11.59 (s, 1H); ^13^C DEPT-135 NMR (101 MHz, CDCl_3_) *δ* ppm 28.1, 116.5, 120.9, 139.3, 150.3.
∗ *2-[2,3-Di(tert-butoxycarbonyl)guanidino]-6-(3-pyridyl)pyridine* (**4s**). Following general method A and starting from 6-(pyridin-3-yl)pyridin-2-amine **5k** (15 mg, 0.07 mmol), **4s** was obtained as a white solid (20 mg, 62%) and immediately used in the BOC deprotection step without analytical characterization.
∗ *2-[2,3-Di(tert-butoxycarbonyl)guanidino]-6-(2-chloropyridin-3-yl)pyridine* (**4t**). Following general method A and starting from **5l** (75 mg, 0.36 mmol), **4t** was obtained as a white solid (121 mg, 0.27 mmol, 74%). ^1^H-NMR (400 MHz, CDCl_3_) *δ* ppm 1.52 (s, 18H), 7.33 (dd, *J* = 7.6, 4.8 Hz, 1H), 7.48 (d, *J* = 7.1 Hz, 1H), 7.80 (t, *J* = 7.9 Hz, 1H), 7.96 (d, *J* = 7.0 Hz, 1H), 8.41 (dd, *J* = 4.7, 1.9 Hz, 2H), 10.85 (s, 1H), 11.57 (s, 1H).
∗ *2-[2,3-Di(tert-butoxycarbonyl)guanidino]-6-(2-methoxypyridin-3-yl)pyridine* (**4u**). Following general method A and starting from **5m** (145 mg, 0.72 mmol), **4u** was obtained as a white solid (241 mg, 0.54 mmol, 76%). ^1^H-NMR (400 MHz, CDCl_3_) *δ* ppm 1.54 (s, 18H), 4.03 (s, 3H), 7.02 (dd, *J* = 7.2, 5.1 Hz, 1H), 7.78 (dt, *J* = 15.6, 7.6 Hz, 2H), 8.19 (dd, *J* = 4.9, 1.9 Hz, 1H), 8.33 (d, *J* = 5.6 Hz, 1H), 10.82 (s, 1H), 11.59 (s, 1H).
∗ *6-Benzyl-1-[2,3-di(tert-butoxycarbonyl)guanidino]pyridine* (**16**). Following general method A and starting from 15 (111 mg, 0.60 mmol), 16 was obtained as a white solid (73 mg, 0.17 mmol, 29%). 1H-NMR (300 MHz, CDCl_3_) δ ppm 1.52 (s, 9H), 1.55 (s, 9H), 4.05 (s, 2H), 6.80 (d, 1H, *J* = 7.6 Hz), 7.20–7.32 (m, 6H), 7.58 (t, 1H, *J* = 7.6 Hz).

#### 4.1.3. General Method B: Pd-Catalyzed Suzuki-Miyaura Cross-Coupling Using Pd(PPh_3_)_4_. Preparation of **5a**, **5b**, **35b**–**g**, **45a**, **45b**

Under argon a microwave vial was charged with the corresponding halogeno derivative (1.0 equiv.), the corresponding phenylboronic acid (1.2 equiv.), Pd(PPh_3_)_4_ (5 mol%), Na_2_CO_3_ (3.0 equiv.) and a mixture toluene:ethanol:water (5:1:1, 5.32 mL/mmol)). The vial was capped properly, flushed with argon and heated to 120 °C until complete conversion of the starting material. After it was cooled, the reaction mixture was concentrated under vacuum. The crude residue was diluted in water. The organic phase was extracted 3 times with EtOAc. The organic layers were combined, washed with brine, dried over Na_2_SO_4_, filtered, concentrated and purified by silica gel column chromatography, eluting with the appropriate hexane:EtOAc mixture.

∗ *6-Phenylpyridin-2-amine* (**5a**) [21]. Following general method B and starting from 2-amino-6-chloropyridine (**2a**, 200 mg, 1.56 mmol) and phenylboronic acid (228 mg, 1.87 mmol), **5a** was obtained as a yellow oil (242 mg, 1.42 mmol, 92%). ^1^H-NMR (400 MHz, CDCl_3_) *δ* ppm 4.54 (s, 2H), 6.45 (d, 1H, *J* = 7.8 Hz), 7.10 (d, 1H, *J* = 7.8 Hz), 7.38 (t, 1H, *J* = 7.7 Hz), 7.44 (t, 2H, *J* = 7.7 Hz), 7.50 (t, 1H, *J* = 7.8 Hz), 7.94 (d, 2H, *J* = 7.7 Hz); ^13^C-NMR (101 MHz, CDCl_3_) δ ppm 107.1, 111.0, 126.8, 128.5, 128.6, 138.4, 139.7, 156.2, 158.3.
∗ *6-(2-Fluorophenyl)pyridin-2-amine* (**5b**). Following general method B and starting from 2-amino-6-chloropyridine (**2a**, 200 mg, 1.56 mmol) and 2-fluorophenylboronic acid (261 mg, 1.87 mmol), **5b** was obtained as a white solid (274 mg, 1.45 mmol, 93%). ^1^H-NMR (400 MHz, CDCl_3_) *δ* ppm 4.52 (s, 2H), 6.48 (d, 1H, *J* = 8.2 Hz), 7.10–7.16 (m, 2H), 7.22 (td, 1H, *J* = 1.3 Hz, *J* = 7.8 Hz), 7.30–7.36 (m, 1H), 7.50 (t, 1H, *J* = 7.8 Hz), 7.90 (td, 1H, *J* = 1.9 Hz, *J* = 7.8 Hz); ^19^F-NMR (376 MHz, CDCl_3_) δ ppm −116.6; ^13^C-NMR (101 MHz, CDCl_3_) δ ppm 107.5, 115.0 (d, *J* = 8.8 Hz), 116.1 (d, *J* = 23.5 Hz), 124.3 (d, *J* = 3.7 Hz), 127.7 (d, *J* = 11.7 Hz), 129.9 (d, *J* = 8.8 Hz), 130.8 (d, *J* = 2.9 Hz), 138.1, 151.7, 158.3, 160.4 (d, *J* = 248.7 Hz).
∗ *6-(2-Methylphenyl)pyridin-2-amine* (**5d**). Following general method B and starting from 2-amino-6-chloropyridine (**2a**, 150 mg, 1.17 mmol) and 2-tolylboronic acid (190 mg, 1.40 mmol), **5d** was obtained as a yellow solid (195 mg, 1.06 mmol, 90%). ^1^H-NMR (400 MHz, CDCl_3_) *δ* ppm 2.27 (s, 3H), 4.43 (s, 2H), 6.37 (d, 1H, *J* = 7.9 Hz), 6.65 (d, 1H, *J* = 7.3 Hz), 7.15–7.19 (m, 3H), 7.26–7.29 (m, 1H), 7.41 (t, 1H, *J* = 7.9 Hz); ^13^C-NMR (101 MHz, CDCl_3_) δ ppm 20.3, 106.5, 114.3, 125.7, 127.9, 129.3, 130.6, 135.6, 137.8, 140.8, 157.8, 158.6.
∗ *6-[2-(Trifluoromethyl)phenyl]pyridin-2-amine* (**5e**). Following general method B and starting from 2-amino-6-chloropyridine (**2a**, 200 mg, 1.56 mmol) and 2-trifluoromethylphenylboronic acid (355 mg, 1.87 mmol), **5e** was obtained as a white solid (325 mg, 1.36 mmol, 88%). ^1^H-NMR (400 MHz, CDCl_3_) *δ* ppm 4.52 (s, 2H), 6.50 (dd, 1H, *J* = 0.6 Hz, *J* = 8.3 Hz), 6.75 (d, 1H, *J* = 7.4 Hz), 7.46–7.50 (m, 3H), 7.58 (t, 1H, *J* = 7.4 Hz), 7.73 (d, 1H, *J* = 7.4 Hz); ^19^F-NMR (376 MHz, CDCl_3_) δ ppm -56.9; ^13^C-NMR (101 MHz, CDCl_3_) δ ppm 107.5, 114.2 (q, *J* = 2.2 Hz), 124.1 (q, *J* = 274.4 Hz), 126.3 (q, *J* = 5.1 Hz), 128.0, 131.3, 131.5, 137.7, 140.3, 156.2, 157.7.
∗ *6-(2,3-Dichlorophenyl)pyridin-2-amine (***5g**). Following general method B and starting from 2-amino-6-bromopyridine (**2b**, 200 mg, 1.16 mmol) and 2,3-dichlorophenylboronic acid (265 mg, 1.39 mmol), **5g** was obtained as a yellow solid (178 mg, 0.74 mmol, 64%). ^1^H-NMR (400 MHz, CDCl_3_) *δ* ppm 4.55 (s, 2H), 6.51 (d, 1H, *J* = 8.0 Hz), 6.87 (d, 1H, *J* = 8.0 Hz), 7.25 (t, 1H, *J* = 7.8 Hz), 7.38 (dd, 1H, *J* = 1.6 Hz, *J* = 7.8 Hz), 7.47 (dd, 1H, *J* = 1.6 Hz, *J* = 7.8 Hz), 7.51 (t, 1H, *J* = 8.0 Hz); ^13^C-NMR (101 MHz, CDCl_3_) δ ppm 107.7, 114.8, 127.2, 129.3, 130.0, 130.8, 133.6, 137.8, 141.9, 155.3, 158.1.
∗ *6-(2,4-Dichlorophenyl)pyridin-2-amine* (**5h**). Following general method B and starting from 2-amino-6-bromopyridine (**2b**, 200 mg, 1.16 mmol) and 2,4-dichlorophenylboronic acid (265 mg, 1.39 mmol), **5h** was obtained as a white solid (207 mg, 0.86 mmol, 75%). ^1^H-NMR (400 MHz, CDCl_3_) *δ* ppm 4.56 (s, 2H), 6.50 (dd, 1H, *J* = 0.5 Hz, *J* = 8.2 Hz), 6.92 (dd, 1H, *J* = 0.5 Hz, *J* = 8.2 Hz), 7.31 (dd, 1H, *J* = 2.0 Hz, *J* = 8.4 Hz), 7.46 (d, 1H, *J* = 2.0 Hz), 7.47–7.52 (m, 2H); ^13^C-NMR (101 MHz, CDCl_3_) δ ppm 107.7, 114.9, 127.2, 129.8, 132.2, 132.9, 134.4, 137.7, 138.0, 154.2, 158.2.
∗ *6-(2,5-Dichlorophenyl)pyridin-2-amine* (**5i**). Following general method B and starting from 2-amino-6-bromopyridine (**2b**, 50 mg, 0.29 mmol) and 2,5-dichlorophenylboronic acid (66 mg, 0.35 mmol), **5i** was obtained as a white solid (56 mg, 0.23 mmol, 81%). ^1^H-NMR (400 MHz, CDCl_3_) *δ* ppm 4.54 (s, 2H), 6.51 (dd, 1H, *J* = 0.4 Hz, *J* = 7.9 Hz), 6.95 (dd, 1H, *J* = 0.4 Hz, *J* = 7.9 Hz), 7.26 (dd, 1H, *J* = 2.5 Hz, *J* = 8.5 Hz), 7.37 (d, 1H, *J* = 8.5 Hz), 7.51 (t, 1H, *J* = 7.9 Hz), 7.55 (d, 1H, *J* = 2.6 Hz); ^13^C-NMR (101 MHz, CDCl_3_) δ ppm 107.8, 114.9, 129.1, 130.5, 131.1, 131.2, 132.7, 137.8, 140.8, 154.0, 158.2.
∗ *6-(Pyridin-4-yl)pyridin-2-amine* (**5j**). Following general method B and starting from 2-amino-6-chloropyridine (**2a**, 100 mg, 0.78 mmol) and 4-pyridineboronic acid (115 mg, 0.93 mmol), **5j** was obtained as a yellow solid (114 mg, 0.66 mmol, 85%). ^1^H-NMR (400 MHz, DMSO-d6) *δ* ppm 6.14 (s, 2H), 6.53 (d, 1H, *J* = 8.0 Hz), 7.20 (d, 1H, *J* = 8.0 Hz), 7.52 (t, 1H, *J* = 8.0 Hz), 7.92 (d, 2H, *J* = 6.1 Hz), 8.62 (d, 2H, *J* = 6.1 Hz); ^13^C-NMR (101 MHz, DMSO-d6) δ ppm 109.5, 121.0, 138.6, 146.7, 150.5, 152.0, 160.2.
∗ *6-(Pyridin-3-yl)pyridin-2-amine (***5k**). Following general method B and starting from 2-amino-6-chloropyridine (**2a**, 20.5 mg, 0.16 mmol) and 3-pyridineboronic acid (23.1 mg, 0.19 mmol), **5k** was obtained as a white powder (17 mg, 0.1 mmol, 62%). ^1^H-NMR (500 MHz, CDCl_3_) δ ppm 4.63 (bs, 2H), 6.49 (d, *J* = 5 Hz, 1H), 7.09 (d, *J* = 5 Hz, 1H), 7.35 (m, 1H), 7.52 (t, *J* = 5 Hz, 1H), 8.23 (dt, *J* = 5 Hz, 1H), 8.60 (d, *J* = 5 Hz, 1H), 9.14 (s, 1H); ^13^C-NMR (125 MHz, CDCl_3_) δ ppm 107.9, 110.9, 123.4, 134.2, 138.5, 148.3, 149.5, 153.3, 158.5. Analytical data are consistent with previously reported characterization [41].
∗ *6-(2-chloropyridin-3-yl)pyridin-2-amine* (**5l**). Following general method B and starting from 2-amino-6-bromopyridine (**2b**, 173 mg, 0.58 mmol) and 2-chloropyridin-3-ylboronic acid (109 mg, 0.69 mmol), **5k** was obtained as a beige solid (75 mg, 0.66 mmol, 63%). Crude solid was triturated in Et_2_O and used in the next step without further purification. ^1^H-NMR (400 MHz, CDCl_3_) *δ* ppm 4.55 (bs, 2H), 6.55 (dd, *J* = 8.2, 0.8 Hz, 1H), 7.05 (dd, *J* = 7.4, 0.7 Hz, 1H), 7.34 (dd, *J* = 7.6, 4.8 Hz, 1H), 7.55 (dd, *J* = 8.2, 7.5 Hz, 1H), 7.92 (dd, *J* = 7.6, 2.0 Hz, 1H), 8.42 (dd, *J* = 4.8, 2.0 Hz, 1H).
∗ *6-(2-methoxypyridin-3-yl)pyridin-2-amine* (**5m**). Following general method B and starting from 2-amino-6-chloropyridine **2a** (100 mg, 0.78 mmol) and 2-methoxypyridin-3-ylboronic acid (143 mg, 0.93 mmol), **5l** as a yellow solid (143 mg, 0.71 mmol, 91%). Crude solid was triturated in Et_2_O and used without further purification. ^1^H-NMR (400 MHz, CDCl_3_) δ 3.74 (s, 3H), 4.21 (bs, 2H), 6.48 (dd, *J* = 8.1, 0.6 Hz, 1H), 7.00 (dd, *J* = 7.4, 5.0 Hz, 1H), 7.33 (dd, *J* = 7.6, 0.7 Hz, 1H), 7.50 (t, *J* = 7.8 Hz, 1H), 8.24–8.08 (m, 2H).
∗ *2-Amino-6-(4-chlorophenyl)benzonitrile* (**35b**). Following general method B and starting from 2-amino-6-iodobenzonitrile (**34**, 200 mg, 0.82 mmol) and 4-chlorophenylboronic acid (154 mg, 0.98 mmol), **35b** was obtained as a white solid (165 mg, 0.72 mmol, 88%). ^1^H-NMR (400 MHz, CDCl_3_) *δ* ppm 4.54 (s 2H), 6.73–6.75 (m, 2H), 7.35 (t, 1H, *J* = 8.0 Hz), 7.43 (d, 2H, *J* = 8.8 Hz), 7.47 (d, 2H, *J* = 8.8 Hz); ^13^C-NMR (101 MHz, CDCl_3_) *δ* ppm 113.9, 117.1, 118.8, 128.8, 129.9, 133.6, 134.7, 137.3, 144.6, 150.7. Analytical data are consistent with the previously reported characterization [42].
∗ *2-Amino-6-(4-methoxyphenyl)benzonitrile (***35c**). Following general method B and starting from 2-amino-6-iodobenzonitrile (**34**, 200 mg, 0.82 mmol) and 4-methoxyphenylboronic acid (149 mg, 0.98 mmol), **35c** was obtained as a yellow solid (169 mg, 0.76 mmol, 92%). ^1^H-NMR (400 MHz, CDCl_3_) *δ* ppm 3.85 (s, 3H), 4.51 (s, 2H), 6.69 (dd, 1H, *J* = 0.9 Hz, *J* = 8.0 Hz), 6.76 (dd, 1H, *J* = 0.9 Hz, *J* = 8.0 Hz), 6.99 (d, 2H, *J* = 8.8 Hz), 7.32 (t, 1H, *J* = 8.0 Hz), 7.49 (d, 2H, *J* = 8.8 Hz); ^13^C-NMR (101 MHz, CDCl_3_) *δ* ppm 55.3, 113.1, 114.0, 117.6, 118.8, 129.8, 131.3, 133.4, 145.6, 150.6, 159.9. Analytical data are consistent with the previously reported characterization [42].
∗ *2-Amino-6-(3-chlorophenyl)benzonitrile (***35d**). Following general method B and starting from 2-amino-6-iodobenzonitrile (**34**, 200 mg, 0.82 mmol) and 3-chlorophenylboronic acid (154 mg, 0.98 mmol), **35d** was obtained as a yellow solid (176 mg, 0.77 mmol, 94%). ^1^H-NMR (400 MHz, CDCl_3_) *δ* ppm 4.56 (s, 2H), 6.75 (d, 2H, *J* = 7.9 Hz), 7.35 (t, 1H, *J* = 7.9 Hz), 7.38–7.45 (m, 3H), 7.49–7.50 (m, 1H); ^13^C-NMR (101 MHz, CDCl_3_) *δ* ppm 114.1, 117.0, 118.8, 126.8, 128.6, 128.7, 129.8, 133.6, 134.5, 140.6, 144.3, 150.7.
∗ *2-Amino-6-(3-methoxyphenyl)benzonitrile* (**35e**). Following general method B and starting from 2-amino-6-iodobenzonitrile (**34**, 200 mg, 0.82 mmol) and 3-methoxyphenylboronic acid (149 mg, 0.98 mmol), **35e** was obtained as a yellow solid (161 mg, 0.72 mmol, 87%). ^1^H-NMR (400 MHz, CDCl_3_) *δ* ppm 3.86 (s, 3H), 4.53 (s, 2H), 6.7 3 (dd, 1H, *J* = 0.9 Hz, *J* = 8.3 Hz), 6.79 (dd, 1H, *J* = 0.9 Hz, *J* = 7.7 Hz), 6.96 (ddd, 1H, *J* = 0.9 Hz, *J* = 2.6 Hz, *J* = 8.3 Hz), 7.07 (dd, 1H, *J* = 1.6 Hz, *J* = 2.6 Hz), 7.12 (ddd, 1H, *J* = 0.9 Hz, *J* = 1.6 Hz, *J* = 7.7 Hz), 7.32–7.39 (m, 2H); ^13^C-NMR (101 MHz, CDCl_3_) *δ* ppm 55.4, 113.6, 114.1, 114.3, 117.3, 118.9, 121.0, 129.6, 133.4, 140.2, 145.7, 150.6, 159.6.
∗ *2-Amino-6-(2-chlorophenyl)benzonitrile (***35f**). Following general method B and starting from 2-amino-6-iodobenzonitrile (**34**, 393 mg, 1.61 mmol) and 2-chlorophenylboronic acid (302 mg, 1.93 mmol), **35f** was obtained as an orange solid (319 mg, 1.40 mmol, 87%). ^1^H-NMR (400 MHz, CDCl_3_) *δ* ppm 4.51 (s, 2H), 6.71 (dd, 1H, *J* = 0.9 Hz, *J* = 7.4 Hz), 6.77 (dd, 1H, *J* = 0.9 Hz, *J* = 8.3 Hz), 7.32–7.38 (m, 4H), 7.47–7.52 (m, 1H); ^13^C-NMR (101 MHz, CDCl_3_) *δ* ppm 114.2, 116.5, 119.6, 126.8, 129.8, 129.9, 131.0, 132.9, 133.2, 137.7, 143.3, 150.0.
∗ *2-Amino-6-(2-methoxyphenyl)benzonitrile* (**35g**). Following general method B and starting from 2-amino-6-iodobenzonitrile (**34**, 300 mg, 1.23 mmol) and 2-methoxyphenylboronic acid (224 mg, 1.48 mmol), **35g** was obtained as a white solid (221 mg, 0.99 mmol, 80%). ^1^H-NMR (400 MHz, CDCl_3_) *δ* ppm 3.85 (s, 3H), 4.45 (s, 2H), 6.73 (dd, 2H, *J* = 1.0 Hz, *J* = 8.3 Hz), 7.01 (d, 1H, *J* = 8.3 Hz), 7.03 (td, 1H, *J* = 1.0 Hz, *J* = 7.5 Hz), 7.24 (dd, 1H, *J* = 1.8 Hz, *J* = 7.4 Hz), 7.34 (t, 1H, *J* = 7.9 Hz), 7.39 (ddd, 1H, *J* = 1.8 Hz, *J* = 7.5 Hz, *J* = 8.2 Hz); ^13^C-NMR (101 MHz, CDCl_3_) *δ* ppm 55.5, 111.3, 113.6, 117.2, 119.8, 120.7, 127.9, 130.0, 130.8, 133.2, 142.9, 149.8, 156.5.
∗ *2-nitro-3-phenylaniline* (**45a***).* Following general method B and starting from 3-bromo-2-nitroaniline (**44a**, 600 mg, 2.77 mmol) and phenylboronic acid (405 mg, 3.12 mmol) **45a** was obtained as an orange solid (482 mg, 2.25 mmol, 81%).^1^H-NMR (400 MHz, CDCl_3_) δ ppm 4.86 (s, 2H), 6.57 (dd, 1H, *J* = 1.1 Hz, *J* = 7.4 Hz), 6.67 (dd, 1H, *J* = 1.0 Hz, *J* = 8.3 Hz), 7.15 (d, 1H, *J* = 8.3 Hz), 7.18 (dd, 2H, *J* = 1.9 Hz, *J* = 7.4 Hz), 7.23–7.30 (m, 3H). ^13^C-NMR (101 MHz, CDCl_3_) δ ppm 117.1, 120.6, 127.4, 127.8, 128.6, 132.4, 138.5, 138.7, 141.8. Analytical data are consistent with the previously reported ^1^H-NMR characterization [43].
∗ *3-(4-Chlorophenyl)-2-nitroaniline* (**45b**). Following general method B and starting from 3-bromo-2-nitroaniline (**44a**, 100 mg, 0.46 mmol) and 4-chlorophenylboronic acid (86 mg, 0.55 mmol), **45b** was obtained as an orange solid (89 mg, 0.36 mmol, 78%). ^1^H-NMR (400 MHz, CDCl_3_) *δ* ppm 5.09 (s, 2H), 6.66 (d, 1H, *J* = 7.3 Hz), 6.83 (d, 1H, *J* = 8.3 Hz), 7.24–7.32 (m, 3H), 7.39 (d, 2H, *J* = 8.3 Hz); ^13^C-NMR (101 MHz, CDCl_3_) *δ* ppm 117.5, 120.5, 128.7, 128.8, 132.6, 133.9, 137.3, 137.4, 142.1.

#### 4.1.4. General Method C: Pd-Catalyzed Suzuki-Miyaura Cross-Coupling Using Pd(OAc)_2_ and S-Phos for the Preparation of **45c**–**g**

A microwave vial under argon was charged with the corresponding halogeno derivatives (1.0 equiv.), the corresponding phenylboronic acid (1.2 equiv.), Pd(OAc)_2_ (2–10 mol%), S-Phos (4–20 mol%), Na_2_CO_3_ (3.0 equiv.) and a mixture CH_3_CN:H_2_O (7:3; 3.33 mL/mmol). The vial was capped properly, flushed with argon and heated to 100°C until complete conversion of the starting material. After it was cooled, the reaction mixture was concentrated under vacuum. The crude residue was diluted in water. The organic phase was extracted three times with EtOAc. The organic layers were combined, washed with brine, dried over Na_2_SO_4_, filtered, concentrated and purified by silica gel column chromatography, eluting with the appropriate hexane:EtOAc mixture.

∗ *2-Nitro-3-phenylaniline (***45a**). Following general method C and starting from 3-bromo-2-nitroaniline (**44a**, 100 mg, 0.92 mmol) and phenylboronic acid (135 mg, 1.11 mmol) **45a** was obtained as an orange solid (95 mg, 0.44 mmol, 48%). Analytical data are consistent with previously reported characterization (see method B). Besides **45a**, *2-nitro-N^1^-(2-nitro-[1,1′-biphenyl]-3-yl)benzene-1,3-diamine* was also isolated as a red solid (50 mg, 0.14 mmol, 30%).^1^H-NMR (400 MHz, CDCl_3_) *δ* ppm 6.03 (s, 2H), 6.26 (d, 1H, *J* = 8.2 Hz), 6.50 (d, 1H, *J* = 8.2 Hz), 7.08 (t, 1H, *J* = 8.2 Hz), 7.14 (d, 1H, *J* = 7.5 Hz), 7.35–7.48 (m, 6H), 7.52 (d, 1H, *J* = 7.5 Hz), 9.92 (s, 1H). ^13^C-NMR (101 MHz, CDCl_3_) *δ* ppm *δ* 104.5, 108.9, 122.9, 126.1, 127.7, 128.6, 128.9, 131.2, 134.1, 135.0, 136.8, 137.1, 141.8, 146.6.
∗ *3-(4-Chlorophenyl)-2-nitroaniline (***45b**). Following general method C and starting from 3-bromo-2-nitroaniline (**44a**, 150 mg, 0.69 mmol) and 2-chlorophenylboronic acid (130 mg, 0.83 mmol), **45b** was obtained as an orange solid (29 mg, 0.12 mmol, 17%). Analytical data are consistent with previously reported characterization (see method B). Besides **45b**, *1-N-[3-(4-chlorophenyl)-2-nitrophenyl]-2-nitrobenzene-1,3-diamine* was also isolated as a red solid (70 mg, 0.18 mmol, 52%). ^1^H-NMR (400 MHz, CDCl_3_) *δ* ppm 5.97 (br s, 2H), 6.23 (d, 1H, *J* = 8.4 Hz), 6.46 (d, 1H, *J* = 8.4 Hz), 7.02–7.06 (m, 2H), 7.21–7.26 (m, 2H), 7.35–7.37 (m, 2H), 7.41 (t, 1H, *J* = 8.0 Hz), 7.49 (d, 1H, *J* = 8.0 Hz), 9.88 (s, 1H). ^13^C-NMR (101 MHz, CDCl_3_) *δ* ppm 104.6, 109.2, 123.0, 125.8, 129.0, 129.2, 131.3, 134.4, 134.8, 134.9, 135.3, 136.0, 139.4, 141.5, 146.6.
∗ *3-(4-Methoxyphenyl)-2-nitroaniline* (**45c**). Following general method C and starting from 3-bromo-2-nitroaniline (**44a**, 150 mg, 0.69 mmol) and 4-methoxyphenylboronic acid (126 mg, 0.83 mmol), **45c** was obtained as a yellow solid (112 mg, 0.46 mmol, 66%). ^1^H-NMR (400 MHz, CDCl_3_) *δ* ppm 3.87 (s, 3H), 4.93 (s, 2H), 6.72 (dd, 1H, *J* = 1.2 Hz, *J* = 7.5 Hz), 6.79 (dd, 1H, *J* = 1.2 Hz, *J* = 8.3 Hz), 6.96 (d, 2H, *J* = 8.8 Hz), 7.27 (d, 2H, *J* = 8.8 Hz), 7.27–7.31 (m, 1H); ^13^C-NMR (101 MHz, CDCl_3_) *δ* ppm 55.0, 113.8, 116.3, 120.2, 128.3, 130.6, 131.9, 137.6, 141.2, 159.1.
∗ *3-(2-Chlorophenyl)-2-nitroaniline* (**45d**). Following general method C and starting from 3-bromo-2-nitroaniline (**44a**, 150 mg, 0.69 mmol) and 2-chlorophenylboronic acid (130 mg, 0.83 mmol), **45d** was obtained as a yellow solid (149 mg, 0.60 mmol, 87%). ^1^H-NMR (400 MHz, CDCl_3_) *δ* ppm 5.40 (s, 2H), 6.54 (dd, 1H, *J* = 1.3 Hz, *J* = 7.4 Hz), 6.82 (dd, 1H, *J* = 1.3 Hz, *J* = 8.3 Hz), 7.20–7.30 (m, 4H), 7.35–7.39 (m, 1H); ^13^C-NMR (101 MHz, CDCl_3_) *δ* ppm 118.4, 120.8, 126.8, 128.8, 129.3, 129.7, 132.4, 133.0, 136.5, 138.5, 143.0.
∗ *3-(2,3-Dichlorophenyl)-2-nitroaniline* (**45e**). Following general method C and starting from 3-bromo-2-nitroaniline (**44a**, 200 mg, 0.92 mmol) and 2,3-dichlorophenylboronic acid (211 mg, 1.11 mmol), **45e** was obtained as a yellow solid (166 mg, 0.59 mmol, 64%). ^1^H-NMR (400 MHz, CDCl_3_) *δ* ppm 5.55 (s, 2H), 6.54 (dd, 1H, *J* = 1.3 Hz, *J* = 7.9 Hz), 6.87 (dd, 1H, *J* = 1.3 Hz, *J* = 7.9 Hz), 7.15 (dd, 1H, *J* = 1.6 Hz, *J* = 8.0 Hz), 7.24 (t, 1H, *J* = 7.9 Hz), 7.33 (t, 1H, *J* = 8.0 Hz), 7.45 (dd, 1H, *J* = 1.6 Hz, *J* = 8.0 Hz); ^13^C-NMR (101 MHz, CDCl_3_) *δ* ppm 118.7, 120.5, 127.3, 127.8, 129.6, 131.0, 133.1, 133.2, 136.3, 140.9, 143.4.
∗ *3-(2,5-Dichlorophenyl)-2-nitroaniline (***45f**). Following general method C and starting from 3-bromo-2-nitroaniline (**44a**, 200 mg, 0.92 mmol) and 2,5-dichlorophenylboronic acid (211 mg, 1.11 mmol), **45f** was obtained as a yellow solid (120 mg, 0.42 mmol, 46%). ^1^H-NMR (400 MHz, CDCl_3_) *δ* ppm 5.58 (s, 2H), 6.54 (dd, 1H, *J* = 1.3 Hz, *J* = 7.3 Hz), 6.88 (dd, 1H, *J* = 1.3 Hz, *J* = 8.4 Hz), 7.24–7.28 (m, 2H), 7.31–7.35 (m, 2H); ^13^C-NMR (101 MHz, CDCl_3_) *δ* ppm 119.0, 120.6, 128.8, 129.5, 130.3, 130.8, 132.6, 133.3, 135.4, 140.3, 143.4.
∗ *3-(2-Methoxyphenyl)-2-nitroaniline* (**45g**). Following general method C and starting from 3-bromo-2-nitroaniline (**44a**, 150 mg, 0.69 mmol) and 2-methoxyphenylboronic acid (126 mg, 0.83 mmol), **45g** was obtained as a yellow solid (142 mg, 0.58 mmol, 84%). ^1^H-NMR (400 MHz, CDCl_3_) *δ* ppm 3.68 (s, 3H), 5.13 (s, 2H), 6.62 (dd, 1H, *J* = 1.3 Hz, *J* = 7.4 Hz), 6.75 (dd, 1H, *J* = 1.3 Hz, *J* = 8.3 Hz), 6.85 (d, 1H, *J* = 8.3 Hz), 7.02 (t, 1H, *J* = 7.4 Hz), 7.22–7.33 (m, 3H); ^13^C-NMR (101 MHz, CDCl_3_) *δ* ppm 55.3, 110.5, 117.6, 121.1, 121.2, 128.4, 129.3, 129.6, 132.8, 135.3, 141.9, 155.7.

##### Miscellaneous: Pd-Catalyzed Suzuki-Miyaura Cross-Coupling Using Pd(OAc)_2_ and S-Phos and K_3_PO_4_ for the Preparation of **15**

∗ *6-Benzylpyridin-2-amine (***15**). This preparation was adapted from the method described by Cee et al. [44]. A microwave vial (oven-dried and under argon) was charged with 2-amino-6-bromopyridine (**2b**, 100 mg, 0.58 mmol, 1.0 equiv.), *B*-Bn-9-BBN 0.5 M in THF (2.31 mL, 1.16 mmol, 2 equiv.), Pd(OAc)_2_ (6.5 mg, 0.03 mmol, 5 mol%), S-Phos (24 mg, 0.06 mmol, 10 mol%), K_3_PO_4_ (368 mg, 1.73 mmol, 3.0 equiv.) and anhydrous THF (3 mL). The vial was capped properly, flushed with argon and heated to 100 °C for 1.5 h. After it was cooled, the reaction mixture was concentrated under vacuum. The crude residue was diluted in water. The aqueous phase was extracted 3 times with EtOAc. The organic layers were combined, washed with brine, dried over Na_2_SO_4_, filtered, concentrated and purified by silica gel column chromatography (hexane:EtOAc 1:1 to 0:1), yielding **15** as a yellow solid (105 mg, 0.57 mmol, 99%). ^1^H-NMR (300 MHz, CDCl_3_) *δ* ppm 3.97 (s, 2H), 4.46 (br s, 2H), 6.32 (d, 1H, *J* = 8.1 Hz), 6.41 (d, 1H, *J* = 7.3 Hz), 7.20–7.35 (m, 6H); ^13^C-NMR (101 MHz, CDCl_3_) *δ* ppm 44.2, 106.1, 113.1, 126.2, 128.4, 129.2, 138.3.

#### 4.1.5. General Method D: Pd-Catalyzed Suzuki-Miyaura Cross-Coupling Using Pd(OAc)_2_ and X-Phos Preparation of Compounds **4c**, **6**

A microwave vial under argon was charged with the corresponding halogeno derivatives (1.0 equiv.), the corresponding phenylboronic acid (1.2 equiv.), Pd(OAc)_2_ (6 mol%), S-Phos (7 mol%), Cs_2_CO_3_ (2.5 equiv.) and a mixture *n*-BuOH:H_2_O (4:1, 8.5 mL/mmol). The vial was capped properly, flushed with argon and heated to 50 °C until complete conversion of the starting material. After it was cooled, the reaction mixture was concentrated under vacuum. The crude residue was diluted in water. The organic phase was extracted three times with EtOAc. The organic layers were combined, washed with brine, dried over Na_2_SO_4_, filtered, concentrated and purified by silica gel column chromatography, eluting with the appropriate hexane: EtOAc mixture.

∗ *2-[2,3-Di(tert-butoxycarbonyl)guanidino]-6-(4-trifluoromethylphenyl)pyridine* (**4c**). Following general method D and starting from **3** (200 mg, 0.54 mmol) and 4-trifluoromethylphenylboronic acid (123 mg, 0.65 mmol), **4c** was obtained as a white solid (220 mg, 0.46 mmol, 85%). ^1^H-NMR (400 MHz, CDCl_3_) *δ* ppm 1.56 (s, 18H), 7.51 (d, 1H, *J* = 7.8 Hz), 7.70 (d, 2H, *J* = 8.3 Hz), 7.81 (t, 1H, *J* = 7.8 Hz), 8.12 (d, 2H, *J* = 8.3 Hz), 8.42 (br s, 1H), 10.91 (s, 1H), 11.59 (s, 1H); ^19^F-NMR (376 MHz, CDCl_3_) δ ppm −62.6; ^13^C-NMR DEPT-135 (101 MHz, CDCl_3_) *δ* ppm 28.1, 116.5, 125.5 (q, *J* = 4.0 Hz), 127.1, 139.2.
∗ *2-[2,3-Di(tert-butoxycarbonyl)guanidino]-6-(furan-3-yl)pyridine* (**4g**). Following general method D and starting from **3** (150 mg, 0.40 mmol) and 3-furanboronic acid (54 mg, 0.48 mmol), **4g** was obtained as a yellow solid (71 mg, 0.18 mmol, 44%). ^1^H-NMR (400 MHz, CDCl_3_) *δ* ppm 1.54 (s, 18H), 6.89 (s, 1H), 7.17 (d, 1H, *J* = 7.7 Hz), 7.46 (t, 1H, *J* = 1.8 Hz), 7.68 (t, 1H, *J* = 7.7 Hz), 8.04 (br s, 1H), 8.27 (br s, 1H), 10.78 (s, 1H), 11.56 (s, 1H); ^13^C DEPT-135 NMR (101 MHz, CDCl_3_) *δ* ppm 28.1, 28.2, 108.6 (2C), 116.1, 138.8, 141.5, 143.8.
∗ *2-[2,3-Di(tert-butoxycarbonyl)guanidino]-6-(1-benzofuran-2-yl)pyridine* (**4h**). Following general method D and starting from **3** (150 mg, 0.40 mmol) and benzofuran-2-boronic acid (79 mg, 0.48 mmol), **4h** was obtained as a white solid (87 mg, 0.19 mmol, 47%). ^1^H-NMR (400 MHz, CDCl_3_) *δ* ppm 1.55 (s, 18H), 7.25 (t, 1H, *J* = 7.7 Hz), 7.33 (t, 1H, *J* = 8.2 Hz), 7.47 (s, 1H), 7.54 (d, 1H, *J* = 8.0 Hz), 7.61 (br s, 1H), 7.64 (d, 1H, *J* = 7.7 Hz), 7.80 (t, 1H, *J* = 7.7 Hz), 8.38 (br s, 1H), 10.88 (s, 1H), 11.59 (s, 1H); ^13^C-NMR (101 MHz, CDCl_3_) *δ* ppm 28.2, 78.6, 79.8, 105.1, 111.5, 114.2, 115.2, 121.6, 123.2, 125.2, 128.8, 139.0, 148.8, 152.7, 153.2, 153.8, 154.3, 154,7, 155.3.
∗ *2-[2,3-Di(tert-butoxycarbonyl)guanidino]-6-(2-chloro-3-(trifluoromethyl)phenyl)pyridine* (**4i**). Following general method D and starting from **3** (60 mg, 0.14 mmol) and 2-Chloro-3-(trifluoromethyl)-phenylboronic acid (42 mg, 0.19 mmol), **4i** was obtained as an oil and the crude was immediately used in the deprotection step with TFA.

#### 4.1.6. General Method E. Formation of Guanidinium Trifluoroacetate Salts. Preparation of cpds. **1c**, **1g**–**j**, **1l**–**m**, **1o**–**u** and **17**

The appropriate di-Boc protected guanidine **4** and **16** (1 equiv.) was dissolved in a mixture of TFA:DCM (1:1; 8 mL/mmol). The solution was stirred at room temperature for 2 h. The solution was concentrated under vacuum and purified by reverse phase C18 column chromatography (MeOH/H_2_O + 0.05%TFA).

∗ *1-[6-(4-Trifluoromethylphenyl)pyridin-2-yl]* guanidinium *trifluoroacetate* (**1c**). Following general method E and starting from **4c** (100 mg, 0.21 mmol), **1c** was obtained as a white solid (81 mg, 0.20 mmol, 98%). Purity ≥ 98%; mp = 248–249 °C; ^1^H-NMR (400 MHz, DMSO-d6) δ ppm 7.13 (d, 1H, *J* = 8.0 Hz), 7.81 (d, 1H, *J* = 8.0 Hz), 7.88 (d, 2H, *J* = 8.4 Hz), 8.03 (t, 1H, *J* = 8.0 Hz), 8.16 (d, 2H, *J* = 8.4 Hz), 8.53 (br s, 4H), 11.46 (s, 1H); ^19^F-NMR (376 MHz, CDCl_3_) δ ppm −74.0, −61.2; ^13^C-NMR (101 MHz, DMSO-d6) δ ppm 113.7, 117.5, 126.3 (q, *J* = 3.7 Hz), 128.0, 130.2 (q, *J* = 32.3 Hz), 141.2, 141.9, 152.6, 153.1, 155.8, 159.9 (q, *J* = 31.5 Hz); HRMS (M + H)^+^ 281.1003 (calcd for C_13_H_11_F_3_N_4_H^+^ 281.1009).
∗ *1-[6-(Furan-3-yl)pyridin-2-yl]* guanidinium *trifluoroacetate* (**1g**). Following general method E and starting from **4g** (58 mg, 0.14 mmol), **1g** was obtained as a white solid (36 mg, 0.11 mmol, 80%). Purity ≥ 98%; mp = 219–221 °C; ^1^H-NMR (400 MHz, DMSO-d6+D_2_O) δ ppm 6.90 (d, 1H, *J* = 7.9 Hz), 7.04 (d, 1H, *J* = 1.5 Hz), 7.48 (d, 1H, *J* = 7.9 Hz), 7.78 (t, 1H, *J* = 1.5 Hz), 7.87 (t, 1H, *J* = 7.9 Hz), 8.42 (s, 1H); ^13^C-NMR (101 MHz, DMSO-d6) δ ppm 109.1, 111.9, 116.0, 126.1, 140.8, 143.0, 145.3, 149.1, 152.4, 155.8, 159.9 (q, *J* = 32.3 Hz); HRMS (M + H)^+^ 203.0914 (calcd for C_10_H_10_N_4_OH^+^ 203.0927).
∗ *1-[6-(1-Benzofuran-2-yl)pyridin-2-yl]* guanidinium *trifluoroacetate* (**1h**). Following general method E and starting from **4h** (54 mg, 0.12 mmol), **1h** was obtained as a white solid (31 mg, 0.08 mmol, 71%). Purity ≥ 98%; mp = 269–270 °C; ^1^H-NMR (400 MHz, DMSO-d6) δ ppm 7.07 (d, 1H, *J* = 8.2 Hz), 7.33 (td, 1H, *J* = 1.3 Hz, *J* = 7.8 Hz), 7.42 (td, 1H, *J* = 1.3 Hz, *J* = 7.8 Hz), 7.68 (d, 1H, *J* = 8.2 Hz), 7.73–7.76 (m, 3H), 8.02 (t, 1H, *J* = 7.8 Hz), 8.53 (br s, 4H), 11.44 (s, 1H); ^13^C-NMR (101 MHz, DMSO-d6) δ ppm 106.4, 112.0, 113.6, 115.6, 122.4, 124.1, 126.4, 128.7, 141.1, 145.8, 152.6, 153.7, 155.2, 155.8, 159.8 (q, *J* = 31.5 Hz); HRMS (M + H)^+^ 253.1076 (calcd for C_14_H_12_N_4_OH^+^ 253.1084).
∗ *1-[6-(2-chloro-3-trifluoromethylphenyl)pyridin-2-yl]* guanidinium *trifluoroacetate* (**1i**). Following general method D and starting from **3** (60 mg, 0.14 mmol) and 2-chloro-3-(trifluoromethyl)phenylboronic acid (42 mg, 0.19 mmol), **4i** was obtained as an oil and without purification dissolved in a mixture of TFA:DCM (1:1, 1 mL) according to method E. The solution was stirred at room temperature for 2 h. The solution was concentrated under vacuum and purified by reverse chromatography (MeOH/H_2_O + 0.05%TFA) to yield **1i** as a white solid (27 mg, 22%). ^1^H-NMR (400 MHz, DMSO-d6) δ 7.17 (d, *J* = 8.7 Hz, 1H), 7.52–7.42 (m, 1H), 7.71 (t, *J* = 7.8, 7.8 Hz, 1H), 7.90 (dd, *J* = 7.7, 1.3 Hz, 1H), 8.00 (dd, *J* = 7.9, 1.4 Hz, 1H), 8.05 (t, *J* = 7.9Hz, 1H), 8.35 (bs, 3H), 11.29 (s, 1H). ^13^C-NMR (101 MHz, DMSO-d6) δ 155.6, 153.1, 152.1, 140.8, 140.7, 136.0, 129.5, 128.9, 128.5, 128.3.
∗ *1-[6-(2-Fluorophenyl)pyridin-2-yl] guanidiniumtrifluoroacetate* (**1j**). Following general method E and starting from **4j** (166 mg, 0.39 mmol), **1j** was obtained as a white solid (107 mg, 0.31 mmol, 81%). Purity ≥ 98%; mp = 199–200 °C; ^1^H-NMR (400 MHz, DMSO-d6) δ ppm 7.10 (d, 1H, J = 8.3 Hz), 7.35–7.40 (m, 2H), 7.50–7.57 (m, 2H), 7.81 (td, 1H, *J* = 1.8 Hz, *J* = 7.8 Hz), 8.00 (t, 1H, *J* = 7.9 Hz), 8.60 (br s, 4H), 11.55 (s, 1H); ^19^F-NMR (376 MHz, CDCl_3_) δ ppm −111.7, −74.0; ^13^C-NMR (101 MHz, DMSO-d6) δ ppm 112.9, 117.1 (d, *J* = 22.2 Hz), 119.7 (d, *J* = 5.1 Hz), 125.6 (d, *J* = 3.7 Hz), 126.4 (d, *J* = 10.3 Hz), 130.9 (d, *J* = 2.2 Hz), 131.9 (d, *J* = 8.8 Hz), 140.8, 150.7 (d, *J* = 2.2 Hz), 152.4, 155.9, 158.9, 160.3 (q, J = 32.3 Hz), 161.4; HRMS (M + H)^+^ 231.1034 (calcd for C_12_H_11_FN_4_H^+^ 231.1041).
∗ *1-[6-(2-Methylphenyl)pyridin-2-yl] guanidiniumtrifluoroacetate* (**1l**). Following general method E and starting from **4l** (104 mg, 0.24 mmol), **1l** was obtained as a white solid (76 mg, 0.22 mmol, 92%). Purity ≥ 95%; mp = 168–169 °C; ^1^H-NMR (400 MHz, DMSO-d6) δ ppm 2.32 (s, 3H), 7.08 (d, 1H, *J* = 8.2 Hz), 7.29–7.36 (m, 4H), 7.40 (d, 1H, *J* = 7.3 Hz), 7.97 (t, 1H, *J* = 7.9 Hz), 8.58 (br s, 4H), 11.57 (s, 1H); ^13^C-NMR (101 MHz, DMSO) δ ppm 20.4, 112.0, 120.0, 126.6, 129.1, 129.8, 131.3, 135.6, 139.4, 140.5, 152.1, 156.0, 156.7, 160.4 (q, *J* = 32.3 Hz); HRMS (M + H)^+^ 227.1282 (calcd for C_13_H_14_N_4_H^+^ 227.1291).
∗ *1-[6-(2-Trifluoromethylphenyl)pyridin-2-yl] guanidinium trifluoroacetate* (**1m**). Following general method E and starting from **4m** (291 mg, 0.61 mmol), **1m** was obtained as a white solid (176 mg, 0.45 mmol, 74%). Purity ≥ 98%; mp = 157–158 °C; ^1^H-NMR (400 MHz, DMSO-d6) δ ppm 7.15 (d, 1H, *J* = 7.9 Hz), 7.29 (d, 1H, *J* = 7.5 Hz), 7.60 (d, 1H, *J* = 7.5 Hz), 7.71 (t, 1H, *J* = 7.5 Hz), 7.80 (t, 1H, *J* = 7.5 Hz), 7.89 (d, 1H, *J* = 7.9 Hz), 8.01 (t, 1H, *J* = 7.9 Hz), 8.55 (br s, 4H), 11.63 (s, 1H); ^19^F-NMR (376 MHz, CDCl_3_) δ ppm −74.0, −55.7; ^13^C-NMR (101 MHz, DMSO-d6) δ ppm 113.0, 119.8, 124.6 (q, *J* = 273.6), 127.1 (q, *J* = 5.1 Hz), 127.1 (q, *J* = 30.8 Hz), 129.9, 132.1, 133.2, 138.7, 140.1, 151.9, 155.0, 155.8, 160.5 (q, *J* = 32.3 Hz); HRMS (M + H)^+^ 281.1007 (calcd for C_13_H_11_F_3_N_4_H^+^ 281.1009).
∗ *1-[6-(2,3-Dichlorophenyl)pyridin-2-yl] guanidinium trifluoroacetate* (**1o**). Following general method E and starting from **4o** (153 mg, 0.32 mmol), **1o** was obtained as a white solid (75 mg, 0.19 mmol, 60%). Purity ≥ 98%; mp = 190–191 °C; ^1^H-NMR (400 MHz, DMSO-d6) δ ppm 7.15 (d, 1H, *J* = 8.0 Hz), 7.41 (d, 1H, *J* = 8.0 Hz), 7.49 (t, 1H, *J* = 7.9 Hz), 7.56 (dd, 1H, *J* = 1.6 Hz, *J* = 7.9 Hz), 7.75 (dd, 1H, *J* = 1.6 Hz, *J* = 7.9 Hz), 8.02 (t, 1H, *J* = 8.0 Hz), 8.59 (br s, 4H), 11.69 (s, 1H); ^13^C-NMR (101 MHz, DMSO-d6) δ ppm 113.3, 120.4, 129.0, 129.9, 130.5, 131.4, 133.1, 140.3, 140.7, 152.1, 153.6, 155.9, 160.5 (q, *J* = 33.0 Hz); HRMS (M + H)^+^ 281.0341 (calcd for C_12_H_10_Cl_2_N_4_H^+^ 281.0355).
∗ *1-[6-(2,4-Dichlorophenyl)pyridin-2-yl] guanidiniumtrifluoroacetate* (**1p**). Following general method E and starting from **4p** (205 mg, 0.43 mmol), **1p** was obtained as a white solid (95 mg, 0.24 mmol, 57%). Purity ≥ 98%; mp = 196–197 °C; ^1^H-NMR (400 MHz, DMSO-d6) δ ppm 7.13 (d, 1H, *J* = 8.0 Hz), 7.43 (d, 1H, *J* = 8.0 Hz), 7.57 (dd, 1H, *J* = 2.0 Hz, *J* = 8.3 Hz), 7.64 (d, 1H, *J* = 8.3 Hz), 7.78 (d, 1H, *J* = 2.0 Hz), 8.01 (t, 1H, *J* = 8.0 Hz), 8.57 (br s, 4H), 11.62 (s, 1H); ^13^C-NMR (101 MHz, DMSO-d6) δ ppm 113.2, 120.4, 128.4, 130.2, 132.6, 133.2, 134.8, 136.8, 140.7, 152.2, 152.8, 155.8, 160.5 (q, *J* = 33.0 Hz); HRMS (M + H)^+^ 281.0348 (calcd for C_12_H_10_Cl_2_N_4_H^+^ 281.0355).
∗ *1-[6-(2,5-Dichlorophenyl)pyridin-2-yl] guanidiniumtrifluoroacetate* (**1q**). Following general method E and starting from **4q** (109 mg, 0.23 mmol), **1q** was obtained as a white solid (81 mg, 0.20 mmol, 91%). Purity = 95%; mp = 151–152 °C; ^1^H-NMR (400 MHz, DMSO-d6) δ ppm 7.15 (d, 1H, *J* = 8.0 Hz), 7.45 (d, 1H, *J* = 8.0 Hz), 7.57 (dd, 1H, *J* = 2.5 Hz, *J* = 8.6 Hz), 7.65 (d, 1H, *J* = 8.6 Hz), 7.69 (d, 1H, *J* = 2.5 Hz), 8.01 (t, 1H, *J* = 8.0 Hz), 8.32 (br s, 4H), 11.71 (s, 1H); ^13^C-NMR (101 MHz, DMSO) δ ppm 113.4, 120.6, 130.4, 130.8, 131.4, 132.4, 132.7, 139.5, 140.7, 152.1, 152.6, 155.7, 160.2 (q, *J* = 32.3 Hz); HRMS (M + H)^+^ 281.0343 (calcd for C_12_H_10_Cl_2_N_4_H^+^ 281.0355).
∗ *1-[6-(Pyridin-4-yl)pyridin-2-yl] guanidiniumtrifluoroacetate* (**1r**). Following general method E and starting from **4r** (88 mg, 0.21 mmol), **1s** was obtained as a white solid (70 mg, 0.21 mmol, 100%). Purity ≥ 98%; mp = 208–209 °C; ^1^H-NMR (400 MHz, DMSO-d6) δ ppm 7.21 (d, 1H, *J* = 8.0 Hz), 7.96 (d, 1H, *J* = 8.0 Hz), 8.08 (t, 1H, *J* = 8.0 Hz), 8.15 (d, 2H, *J* = 6.3 Hz), 8.57 (br s, 4H), 8.84 (d, 2H, *J* = 6.3 Hz), 11.61 (s, 1H); ^13^C-NMR (101 MHz, DMSO-d6) δ ppm 115.2, 118.1, 122.3, 141.4, 147.4, 148.7, 151.2, 152.9, 155.7, 159.7 (q, *J* = 33.7 Hz); HRMS (M + H)^+^ 214.1070 (calcd for C_11_H_11_N_5_H^+^ 214.1087).
∗ *1-[6-(Pyridin-3-yl)pyridin-2-yl]* guanidinium *trifluoroacetate (**1s**)*. Following general method E and starting from crude **4s** (20 mg, 0.048 mmol), **1s** was obtained as a white hygroscopic solid (11 mg, 0.02 mmol, 52%). Purity ≥ 97%; ^1^H-NMR (400 MHz, CD_3_OD) δ 7.10 (d, *J* = 7.9 Hz, 1H), 7.74 (d, *J* = 7.4 Hz, 1H), 7.90–7.83 (m, 1H), 7.95 (t, *J* = 8.0 Hz, 1H), 8.73 (d, *J* = 8.0 Hz, 2H), 9.22 (s, 1H). LC/MS (M + H)^+^ = 214.062.
∗ *1-(2′-Chloro-[2,3′-bipyridin]-6-yl) guanidiniumtrifluoroacetate* (**1t**). Following general method E and starting from **4t** (120 mg, 0.027 mmol), **1t** was obtained as a white hygroscopic solid (85 mg, 0.18 mmol, 66%). Purity ≥ 98%;^1^H-NMR (500 MHz, CD_3_OD) δ 7.16 (d, *J* = 8.2 Hz, 1H), 7.54–7.49 (m, 1H), 7.57 (dd, *J* = 7.6, 4.8 Hz, 1H), 8.05–7.99 (m, 1H), 8.07 (dd, *J* = 7.6, 1.9 Hz, 1H), 8.49 (dd, *J* = 4.8, 1.9 Hz, 1H); ^13^C-NMR (126 MHz, MeOD) δ 112.71, 123.17, 123.62, 134.48, 140.02, 140.28, 148.37, 149.44, 151.75, 152.59, 155.75. HRMS (M + H)^+^ 248.0703 (calcd for C_11_H_10_ClN_5_H^+^ 248.0698).
∗ *1-(2′-Methoxy-[2,3′-bipyridin]-6-yl) guanidiniumtrifluoroacetate* (**1u**). Following general method E and starting from **4u** (234 mg, 0.53 mmol), **1u** was obtained as a white hygroscopic solid (70 mg, 0.37 mmol, 70%). Purity ≥ 97%;^1^H-NMR (400 MHz, MeOD) δ4.03 (s, 3H), 7.03 (dd, *J* = 8.2, 0.6 Hz, 1H), 7.14 (dd, *J* = 7.5, 5.0 Hz, 1H), 7.67 (dd, *J* = 7.8, 0.6 Hz, 1H), 7.94 (t, *J* = 8.0 Hz, 1H), 8.07 (dd, *J* = 7.5, 1.9 Hz, 1H), 8.26 (dd, *J* = 5.0, 1.9 Hz, 1H); ^13^C-NMR (101 MHz, MeOD) δ 52.7, 111.5, 117.2, 119.5, 121.6, 138.8, 139.6, 147.4, 151.3, 152.2, 156.1, 160.8. HRMS (M + H)^+^ 244.1198 (calcd for C_12_H_13_N_5_OH^+^ 244.1189).
∗ *1-(6-Benzylpyridin-2-yl) guanidiniumtrifluoroacetate* (**17**). Following general method E and starting from **16** (73 mg, 0.17 mmol), **17** was obtained as a white solid (56 mg, 0.17 mmol, 96%). Purity ≥ 98%; mp = 161–162 °C; ^1^H-NMR (500 MHz, DMSO-d_6_) δ ppm 4.10 (s, 4H), 6.87 (d, 1H, *J* = 7.8 Hz), 7.10 (d, 1H, *J* = 7.8 Hz), 7.22 (t, 1H, *J* = 6.7 Hz), 7.27–7.33 (m, 4H), 7.79 (t, 1H, *J* = 7.8 Hz), 8.41 (br s, 4H), 11.21 (s, 1H); ^13^C-NMR (125 MHz, DMSO) δ ppm 43.2, 111.0, 118.7, 126.8, 129.0, 129.5, 139.6, 140.6, 152.1, 155.8, 158.9, 160.0 (q, *J* = 31.8 Hz); HRMS (M + H)^+^ 227.1285 (calcd for C_13_H_14_N_4_H^+^ 227.1291).

#### 4.1.7. General Method F: Reduction of Aromatic Nitriles to Amines Using the comPlex BH_3_.SMe_2_: Preparation of cpd. **36**

In a one-neck round-bottom flask under argon, the cyano derivative (1 equiv.) was dissolved in THF (13.5 mL/mmol) and BH_3_.SMe_2_ (2 equiv.) was added dropwise. The resulting solution was heated at reflux for 2 h. After it was cooled, 2N HCl was added and the solution was heated at 90 °C for 1 h. After it was cooled, the solution was concentrated under vacuum and purified by reverse chromatography (MeOH/H_2_O + 0.05%TFA). The desired salt was solubilized in a saturated solution of NaHCO_3_ was added to reach a pH of 8. The aqueous phase was extracted twice with EtOAc. The combined organic layers were washed with brine, dried over Na_2_SO_4_, filtered and concentrated.

∗ *2-(Aminomethyl)-3-(4-chlorophenyl)aniline* (**36b**). Following general method F and starting from **35b** (100 mg, 0.44 mmol), **36b** was obtained as a yellow solid (74 mg, 0.32 mmol, 73%). ^1^H-NMR (400 MHz, CDCl_3_) *δ* ppm 2.77 (br s, 4H), 3.78 (s, 2H), 6.61 (d, 1H, *J* = 7.8 Hz), 6.71 (d, 1H, *J* = 7.8 Hz), 7.10 (t, 1H, *J* = 7.8 Hz), 7.21 (d, 2H, *J* = 8.4 Hz), 7.36 (d, 2H, *J* = 8.4 Hz); ^13^C-NMR (101 MHz, CDCl_3_) *δ* ppm 40.6, 115.5, 119.9, 123.4, 127.6, 128.2, 130.4, 132.9, 140.5, 141.6, 147.4.
∗ *2-(Aminomethyl)-3-(4-methoxyphenyl)aniline* (**36c**). Following general method F and starting from **35c** (100 mg, 0.45 mmol), **36c** was obtained as a colorless oil (77 mg, 0.34 mmol, 76%). ^1^H-NMR (400 MHz, CDCl_3_) *δ* ppm 3.85 (s, 3H), 6.64 (dd, 1H, *J* = 0.9 Hz, *J* = 7.7 Hz), 6.69 (dd, 1H, *J* = 0.9 Hz, *J* = 7.7 Hz), 6.93 (d, 2H, *J* = 8.7 Hz), 7.09 (t, 1H, *J* = 7.7 Hz), 7.20 (d, 2H, *J* = 8.7 Hz); ^13^C-NMR (101 MHz, CDCl_3_) *δ* ppm 41.7, 55.3, 113.4, 119.1, 120.3, 127.5, 130.1, 134.4, 142.5, 147.2, 158.7.
∗ *2-(Aminomethyl)-3-(3-chlorophenyl)aniline* (**36d**). Following general method F and starting from **35d** (100 mg, 0.44 mmol), **36d** was obtained as a colorless oil (77 mg, 0.33 mmol, 75%). ^1^H-NMR (400 MHz, CDCl_3_) *δ* ppm 2.88 (br s, 4H), 3.78 (s, 2H), 6.61 (d, 1H, *J* = 7.7 Hz), 6.71 (d, 1H, *J* = 7.7 Hz), 7.10 (t, 1H, *J* = 7.7 Hz), 7.14–7.17 (m, 1H), 7.28–7.33 (m, 3H); ^13^C-NMR (101 MHz, CDCl_3_) *δ* ppm 40.6, 115.6, 119.8, 123.2, 127.0, 127.3, 127.7, 129.1, 129.2, 133.9, 141.4, 143.8, 147.3.
∗ *2-(Aminomethyl)-3-(3-methoxyphenyl)aniline* (**36e**). Following general method F and starting from **35e** (100 mg, 0.45 mmol), **36e** was obtained as a yellow oil (72 mg, 0.32 mmol, 71%). ^1^H-NMR (400 MHz, CDCl_3_) *δ* ppm 3.80 (s, 2H), 3.83 (s, 3H), 6.66 (dd, 1H, *J* = 1.0 Hz, *J* = 7.8 Hz), 6.70 (dd, 1H, *J* = 1.0 Hz, *J* = 7.8 Hz), 6.83–6.91 (m, 3H), 7.10 (t, 1H, *J* = 7.8 Hz), 7.31 (t, 1H, *J* = 7.9 Hz); ^13^C-NMR (101 MHz, CDCl_3_) *δ* ppm 40.6, 55.3, 112.3, 114.8, 115.2, 119.9, 121.6, 123.5, 127.5, 128.9, 142.7, 143.5, 147.2, 159.2.
∗ *2-(Aminomethyl)-3-(2-chlorophenyl)aniline* (**36f**). Following general method F and starting from **35f** (200 mg, 0.87 mmol), **36f** was obtained as a yellow solid (292 mg, 0.84 mmol, 96%). ^1^H-NMR (400 MHz, DMSO-d6 + D_2_O) *δ* ppm 6.47 (dd, 1H, *J* = 1.0 Hz, *J* = 7.8 Hz), 6.86 (dd, 1H, *J* = 1.0 Hz, *J* = 7.8 Hz), 7.17 (t, 1H, *J* = 7.8 Hz), 7.35–7.38 (m, 1H), 7.40–7.44 (m, 2H), 7.51–7.54 (m, 1H); ^13^C-NMR (101 MHz, DMSO) *δ* ppm 36.9, 115.9, 116.3, 127.7, 129.7, 129.9, 130.0, 132.1, 132.7, 139.4, 141.0, 148.0.

##### Miscellaneous: Reduction of Aromatic Nitrile with the Use of LiAlH_4_ and AlCl_3_: Preparation of cpd. **36g**

▪ *2-(Aminomethyl)-3-(2-methoxyphenyl)aniline* (**36g**). In a two-neck round bottom-flask (oven dried and under argon), anhydrous THF (15.0 mL) was introduced and LiAlH_4_ (406 mg, 10.7 mmol, 12 equiv.) was added slowly followed by AlCl_3_ (357 mg, 2.67 mmol, 3 equiv.). The reaction mixture was cooled at 0 °C and stirred 5 min. A solution of **35g** (200 mg, 0.89 mmol, 1 equiv.) in anhydrous THF (5.0 mL) was added dropwise. The resulting solution was stirred overnight at rt. Water was added dropwise followed by H_2_SO_4_ (6N). The solution was stirred 30 min, filtered and concentrated under vacuum. The residue was basified until pH 9 using NaOH. The resulting solid was filtered and washed with water, yielding to **36g** as a brown solid (143 mg, 0.63 mmol, 70%).^1^H-NMR (400 MHz, CDCl_3_) *δ* ppm 2.83 (br s, 4H), 3.67 (s, 3H), 3.69 (s, 2H), 6.48–6.52 (m, 1H), 6.62 (d, 1H, *J* = 7.8 Hz), 6.85–6.88 (m, 1H), 6.91–6.95 (m, 1H), 7.09 (t, 2H, *J* = 7.0 Hz), 7.25 (t, 1H, *J* = 7.8 Hz); ^13^C-NMR (101 MHz, CDCl_3_) *δ* ppm 41.1, 55.6, 110.7, 114.2, 119.3, 120.7, 127.8, 128.5, 131.0, 131.3, 136.9, 138.8, 142.3, 156.5.

#### 4.1.8. General Method G: Reduction of Aromatic Nitro Compounds Using Tin and HCl. Preparation of cpds. **47a**–**g**

In a one-neck round-bottom flask, the aromatic nitro derivative **45** (1 equiv.) was dissolved in ethanol (4.4 mL/mmol). The reaction media was cooled at 0 °C and Tin (2.63 equiv.) was added, followed by small portions of 37% HCl (36 equiv.). The resulting solution was heated at reflux for 1 h. After it was cooled, the reaction mixture was filtered through a pad of Celite^®^ and washed with ethanol. The filtrate was concentrated under vacuum. The residue was dissolved in a saturated solution of NaHCO_3_. The aqueous phase was extracted twice with EtOAc. The combined organic layers were washed with brine, dried over Na_2_SO_4_, filtered and concentrated. Compounds **47** were used without further purification in the next step of the synthesis.

∗ *3-Phenylbenzene-1,2-diamine* (**47a**). Following general method G and starting from **45a** (70 mg, 0.33 mmol), **47b** was obtained as an orange oil (61 mg, 0.33 mmol, 100%).^1^H-NMR (400 MHz, DMSO-d6) *δ* ppm 5.45 (br s, 4H), 6.50 (d, 1H, *J* = 7.5 Hz), 6.57 (t, 1H, *J* = 7.5 Hz), 6.72 (d, 1H, *J* = 7.5 Hz), 7.32–7.47 (m, 5H). ^13^C-NMR (101 MHz, DMSO-d6) *δ* ppm 116.3, 118.3, 121.5, 127.3, 127.9, 129.2, 129.3, 132.7, 132.9, 140.4. Analytical data are consistent with the previously reported characterization [43].
∗ *3-(4-Chlorophenyl)benzene-1,2-diamine* (**47b**). Following general method G and starting from **45b** (85 mg, 0.34 mmol), **47b** was obtained as a white solid (51 mg, 0.23 mmol, 68%). ^1^H-NMR (400 MHz, DMSO-d6) *δ* ppm 4.12 (s, 2H), 4.61 (s, 2H), 6.33 (d, 1H, *J* = 7.5 Hz), 6.49 (t, 1H, *J* = 7.5 Hz), 6.57 (d, 1H, *J* = 7.5 Hz), 7.39 (d, 2H, *J* = 8.3 Hz), 7.47 (d, 2H, *J* = 8.3 Hz); ^13^C-NMR (101 MHz, DMSO-d6) *δ* ppm 114.6, 118.1, 119.2, 125.7, 129.1, 131.1, 131.7, 131.8, 136.0, 139.8. Analytical data are consistent with the previously reported characterization [45].
∗ *3-(4-Methoxyphenyl)benzene-1,2-diamine* (**47c**). Following general method G and starting from **45c** (80 mg, 0.33 mmol), **47c** was obtained as an orange oil (60 mg, 0.28 mmol, 86%). ^1^H-NMR (400 MHz, DMSO-d6) *δ* ppm 3.78 (s, 3H), 4.27 (br s, 4H), 6.33 (dd, 1H, *J* = 1.5 Hz, *J* = 7.7 Hz), 6.47 (t, 1H, *J* = 7.7 Hz), 6.54 (dd, 1H, *J* = 1.5 Hz, *J* = 7.7 Hz), 6.99 (d, 2H, *J* = 8.7 Hz), 7.29 (d, 2H, *J* = 8.7 Hz); ^13^C-NMR (101 MHz, DMSO-d6) *δ* ppm 55.5, 114.2, 114.6, 118.0, 119.4, 126.9, 130.3, 131.8, 133.0, 135.9, 158.5.
∗ *3-(2-Chlorophenyl)benzene-1,2-diamine* (**47d**). Following general method G and starting from **45d** (193 mg, 0.78 mmol), **47d** was obtained as an orange solid (172 mg, 0.78 mmol, 100%). ^1^H-NMR (400 MHz, DMSO-d6) *δ* ppm 3.86 (s, 2H), 4.60 (s, 2H), 6.24 (dd, 1H, *J* = 1.5 Hz, *J* = 7.5 Hz), 6.48 (t, 1H, *J* = 7.5 Hz), 6.58 (dd, 1H, *J* = 1.5 Hz, *J* = 7.5 Hz), 7.26–7.30 (m, 1H), 7.35–7.42 (m, 2H), 7.52–7.55 (m, 1H); ^13^C-NMR (101 MHz, DMSO-d6) *δ* ppm 114.6, 117.6, 119.0, 124.8, 127.9, 129.4, 130.1, 132.2, 132.4, 135.8, 139.2.
∗ *3-(2,3-Dichlorophenyl)benzene-1,2-diamine* (**47e**). Following general method G and starting from **45e** (137 mg, 0.48 mmol), **47e** was obtained as an orange solid (122 mg, 0.48 mmol, 100%). ^1^H-NMR (400 MHz, DMSO-d6) *δ* ppm 5.01 (br s, 4H), 6.33 (dd, 1H, *J* = 1.1 Hz, *J* = 7.5 Hz), 6.51 (t, 1H, *J* = 7.5 Hz), 6.68 (dd, *J* = 1.1 Hz, *J* = 7.5 Hz), 7.24 (dd, 1H, *J* = 1.4 Hz, *J* = 7.9 Hz), 7.40 (t, 1H, *J* = 7.9 Hz), 6.62 (dd, 1H, *J* = 1.4 Hz, *J* = 7.8 Hz); ^13^C-NMR (101 MHz, DMSO-d6) *δ* ppm 116.1, 117.5, 120.3, 124.8, 128.8, 130.0, 131.1, 131.8, 132.6, 133.0, 133.5, 141.5.
∗ *3-(2,5-Dichlorophenyl)benzene-1,2-diamine* (**47f**). Following general method G and starting from **45f** (90 mg, 0.32 mmol), **47f** was obtained as an orange solid (80 mg, 0.32 mmol, 100%). ^1^H-NMR (400 MHz, DMSO-d6) *δ* ppm 4.00 (s, 2H), 4.63 (s, 2H), 6.23 (dd, 1H, *J* = 1.5 Hz, *J* = 7.5 Hz), 6.47 (t, 1H, *J* = 7.5 Hz), 6.58 (dd, 1H, *J* = 1.5 Hz, *J* = 7.5 Hz), 7.30 (d, 1H, *J* = 2.5 Hz), 7.43 (dd, 1H, *J* = 2.5 Hz, *J* = 8.7 Hz), 7.56 (d, 1H, *J* = 8.7 Hz); ^13^C-NMR (101 MHz, DMSO-d6) *δ* ppm 114.9, 117.5, 118.8, 123.4, 129.2, 131.7, 131.9, 132.1, 132.3, 132.4, 135.9, 141.2.
∗ *3-(2-Methoxyphenyl)benzene-1,2-diamine* (**47g**). Following general method G and starting from **45g** (80 mg, 0.33 mmol), **47g** was obtained as an orange solid (69 mg, 0.32 mmol, 98%). ^1^H-NMR (400 MHz, DMSO-d6) *δ* ppm 3.72 (s, 3H), 4.74 (br s, 4H), 6.32 (dd, 1H, *J* = 1.5 Hz, *J* = 7.5 Hz), 6.49 (t, 1H, *J* = 7.5 Hz), 6.60 (dd, 1H, *J* = 1.5 Hz, *J* = 7.5 Hz), 7.00 (t, 1H, *J* = 7.3 Hz), 7.07–7.11 (m, 2H), 7.24 (td, 1H, *J* = 1.9 Hz, *J* = 8.4 Hz); ^13^C-NMR (101 MHz, DMSO-d6) *δ* ppm 55.7, 112.0, 114.9, 117.8, 120.8, 121.1, 125.1, 129.0, 129.1, 131.7, 132.8, 134.6, 156.9.

#### 4.1.9. General Method H. Cyclisation of a Diamino Derivatives 36 and 47 Using BrCN: Preparation of Dihydroquinazolines **37** and Benzimidazoles **49**

An appropriate benzene-1,2 diamine **36** or 2-(aminomethyl)aniline **47** derivative (1 equiv.) was dissolved in toluene (1.5 mL/mmol), followed by dropwise addition of a solution of BrCN (1.5 equiv.) in toluene (1 mL/mmol). The resulting solution was heated at 110 °C for 4 h. After it was cooled, the solution was concentrated under vacuum and immediately purified by reverse C18 phase chromatography (MeOH/H_2_O+0.05%HBr) to afford 5-aryl dihydroquinazolin 2-amine (**37**) and benzimidazole **49**.

∗ *5-(4-Chlorophenyl)-3,4-dihydroquinazolin-2-amine Hydrobromide* (**37b**). Following general method H and starting from **36b** (38 mg, 0.17 mmol), **37b** was obtained as a white solid (44 mg, 0.13 mmol, 79%). Purity ≥ 98 %; mp = 196–197 °C; ^1^H-NMR (400 MHz, DMSO-d6) *δ* ppm 4.36 (s, 2H), 7.05 (d, 1H, *J* = 7.5 Hz), 7.06 (d, 1H, *J* = 7.5 Hz), 7.36 (t, 1H, *J* = 7.5 Hz), 7.37 (d, 2H, *J* = 8.4 Hz), 7.54 (d, 2H, *J* = 8.4 Hz), 7.56 (s, 2H), 8.14 (s, 1H), 10.64 (s, 1H); ^13^C-NMR (101 MHz, DMSO-d6) *δ* ppm 40.2, 115.5, 116.5, 125.8, 129.0, 129.1, 131.0, 133.3, 134.2, 137.7, 138.8, 153.1; HRMS (M + H)^+^ 258.0782 (calcd for C_14_H_12_ClN_3_H^+^ 258.0793).
∗ *5-(4-Methoxyphenyl)-3,4-dihydroquinazolin-2-amine Hydrobromide* (**37c**). Following general method H and starting from **36c** (48 mg, 0.22 mmol), **37c** was obtained as a white solid (51 mg, 0.16 mmol, 71%). Purity ≥ 98%; ^1^H-NMR (400 MHz, DMSO-d6) *δ* ppm 3.80 (s, 3H), 4.37 (s, 2H), 7.00–7.04 (m, 4H), 7.26 (d, 2H, *J* = 8.4 Hz), 7.33 (t, 1H, *J* = 7.8 Hz), 7.55 (s, 2H), 8.15 (s, 1H), 10.62 (s, 1H); ^13^C-NMR (101 MHz, DMSO) *δ* ppm 40.4, 55.7, 114.5, 114.8, 116.5, 125.9, 128.8, 130.3, 131.1, 134.1, 139.9, 153.0, 159.4; HRMS (M + H)^+^ 254.1281 (calcd for C_15_H_15_N_3_OH^+^ 254.1288).
∗ *5-(3-Chlorophenyl)-3,4-dihydroquinazolin-2-amine Hydrobromide* (**37d**). Following general method H and starting from **36d** (15 mg, 0.065 mmol), **37d** was obtained as a white solid (11 mg, 0.032 mmol, 49%). Purity ≥ 98%; mp = 228–230 °C; ^1^H-NMR (400 MHz, DMSO-d6) *δ* ppm 4.38 (s, 2H), 7.07 (d, 2H, *J* = 7.8 Hz), 7.31 (t, 1H, *J* = 3.8 Hz), 7.38 (t, 1H, *J* = 7.8 Hz), 7.42 (s, 1H), 7.50–7.52 (m, 2H), 7.56 (s, 2H), 8.07 (s, 1H), 10.61 (s, 1H); ^13^C-NMR (101 MHz, DMSO-d6) *δ* ppm 115.7, 116.5, 125.9, 127.9, 128.3, 128.8, 129.0, 130.9, 133.7, 134.2, 138.6, 141.0, 153.0; HRMS (M + H)^+^ 258.0780 (calcd for C_14_H_12_ClN_3_H^+^ 258.0793).
∗ *5-(3-Methoxyphenyl)-3,4-dihydroquinazolin-2-amine Hydrobromide* (**37e**). Following general method H and starting from **36e** (19 mg, 0.081 mmol), **37e** was obtained as a yellow solid (18 mg, 0.053 mmol, 65%). Purity ≥ 98%; mp = 198–199 °C; ^1^H-NMR (400 MHz, DMSO-d6) *δ* ppm 3.79 (s, 3H), 4.38 (s, 2H), 6.86–6.89 (m, 2H), 6.99 (dd, 1H, *J* = 2.5 Hz, *J* = 8.0 Hz), 7.03–7.07 (m, 2H), 7.33–7.40 (m, 2H), 7.57 (s, 2H), 8.12 (s, 1H), 10.65 (s, 1H); ^13^C-NMR (101 MHz, DMSO-d6) *δ* ppm 40.3, 55.7, 113.8, 114.7, 115.2, 116.4, 121.3, 125.8, 128.9, 130.1, 134.1, 140.0, 140.3, 153.1, 159.7; HRMS (M + H)^+^ 254.1280 (calcd for C_15_H_15_N_3_OH^+^ 254.1288).
∗ *5-(2-Chlorophenyl)-3,4-dihydroquinazolin-2-amine Hydrobromide* (**37f**). Following general method H and starting from **36f** (62 mg, 0.27 mmol), **37f** was obtained as a white solid (61 mg, 0.18 mmol, 68%). Purity ≥ 98%; mp = 178–179 °C; ^1^H-NMR (400 MHz, DMSO-d6) *δ* ppm 4.07 (d, 1H, *J* = 14.7 Hz), 4.15 (d, 1H, *J* = 14.7 Hz), 6.96 (d, 1H, *J* = 7.9 Hz), 7.08 (d, 1H, *J* = 7.9 Hz), 7.32 (dd, 1H, *J* = 1.9 Hz, *J* = 6.9 Hz), 7.37 (t, 1H, *J* = 7.9 Hz), 7.42–7.50 (m, 2H), 7.58–7.61 (m, 3H), 8.11 (s, 1H), 10.69 (s, 1H); ^13^C-NMR (101 MHz, DMSO-d6) *δ* ppm 40.1, 115.6, 115.7, 116.8, 125.8, 128.0, 128.9, 130.0, 130.6, 131.5, 132.3, 133.9, 137.3, 137.4, 152.8; HRMS (M + H)^+^ 258.0788 (calcd for C_14_H_12_ClN_3_H^+^ 258.0793).
∗ *5-(2-Methoxyphenyl)-3,4-dihydroquinazolin-2-amine Hydrobromide* (**37g**). Following general method H and starting from **36g** (20 mg, 0.086 mmol), **37g** was obtained as an orange solid (20 mg, 0.061 mmol, 70%). Purity ≥ 98%; mp = 226–229 °C; ^1^H-NMR (400 MHz, DMSO-d6) *δ* ppm 3.37 (s, 3H), 4.05 (d, 1H, *J* = 14.6 Hz), 4.17 (d, 1H, *J* = 14.6 Hz), 6.94 (d, 1H, *J* = 7.5 Hz), 7.01 (d, 1H, *J* = 8.0 Hz), 7.05 (d, 1H, *J* = 7.5 Hz), 7.10–7.14 (m, 2H), 7.31 (t, 1H, *J* = 8.0 Hz), 7.42 (td, 1H, *J* = 1.8 Hz, J = 8.0 Hz), 7.55 (br s, 2H), 8.06 (br s, 1H), 10.60 (br s, 1H); ^13^C-NMR (101 MHz, DMSO-d6) *δ* ppm 40.2, 55.9, 111.8, 114.9, 117.6, 121.1, 126.4, 127.4, 128.6, 130.2, 130.9, 133.7, 137.1, 153.0, 156.4; HRMS (M + H)^+^ 254.1281 (calcd for C_15_H_15_N_3_OH^+^ 254.1288).
∗ *4-Phenyl-1H-benzo[d]imidazol-2-amine hydrobromide* (**49a**). Following general method H and starting from **47a** (153 mg, 0.83 mmol), **49a** was obtained as a yellow solid (178 mg, 0.61 mmol, 74%). ^1^H-NMR (400 MHz, DMSO-d6) *δ* ppm 7.27 (d, 1H, *J* = 7.7 Hz), 7.33 (t, 1H, *J* = 7.7 Hz), 7.39 (d, 1H, *J* = 7.7 Hz), 7.48 (t, 1H, *J* = 7.0 Hz), 7.54–7.61 (m, 4H), 8.06 (s, 2H), 12.50 (s, 2H). ^13^C-NMR (101 MHz, DMSO-d6) *δ* ppm 111.2, 123.6, 124.2, 125.8, 127.3, 128.6, 129.7, 130.8, 136.8, 151.7. Analytical data are consistent with previously reported characterization [46].
∗ *4-(4-Chlorophenyl)-1H-benzo[d]imidazol-2-amine Hydrobromide* (**49b**). Following general method H and starting from **47b** (39 mg, 0.18 mmol), **49b** was obtained as a white solid (29 mg, 0.09 mmol, 49%). Purity ≥ 98%; mp = 254–256 °C; ^1^H-NMR (400 MHz, DMSO-d6) *δ* ppm 7.26 (dd, *J* = 0.9 Hz, *J* = 7.8 Hz), 7.33 (t, 1H, *J* = 7.8 Hz), 7.40 (dd, 1H, *J* = 0.9 Hz, *J* = 7.8 Hz), 7.61 (s, 4H), 8.12 (s, 2H), 12.54 (s, 2H); ^13^C-NMR (101 MHz, DMSO-d6) *δ* ppm 111.5, 123.6, 124.2, 124.6, 127.5, 129.6, 130.6, 130.9, 133.4, 135.6, 151.7; HRMS (M + H)^+^ 244.0626 (calcd for C_13_H_10_ClN_3_H^+^ 244.0636).
∗ *4-(4-Methoxyphenyl)-1H-benzo[d]imidazol-2-amine Hydrobromide* (**49c**). Following general method H and starting from **47c** (48 mg, 0.22 mmol), **49c** was obtained as a white solid (51 mg, 0.16 mmol, 71%). Purity ≥ 98%; mp = 218–220 °C; ^1^H-NMR (400 MHz, DMSO-d6) *δ* ppm 3.83 (s, 3H), 7.11 (d, 2H, *J* = 8.7 Hz), 7.22 (d, 1H, *J* = 7.2 Hz), 7.29 (t, 1H, *J* = 7.2 Hz), 7.35 (d, 1H, *J* = 7.2 Hz), 7.53 (d, 2H, *J* = 8.7 Hz), 8.05 (s, 2H), 12.46 (s, 2H); ^13^C-NMR (101 MHz, DMSO-d6) *δ* ppm 55.8, 110.6, 115.1, 123.4, 124.2, 125.6, 127.2, 129.0, 129.8, 130.7, 131.6, 159.7; HRMS (M + H)^+^ 240.1124 (calcd for C_14_H_13_N_3_OH^+^ 240.1131).
∗ *4-(2-Chlorophenyl)-1H-benzo[d]imidazol-2-amine Hydrobromide* (**49d**). Following general method H and starting from **47d** (50 mg, 0.23 mmol), **49d** was obtained as an orange solid (35 mg, 0.11 mmol, 48%). Purity ≥ 98%; mp = 255–256 °C; ^1^H-NMR (400 MHz, DMSO-d6) *δ* ppm 7.13 (d, 1H, *J* = 7.5 Hz), 7.32 (t, 1H, *J* = 7.5 Hz), 7.43–7.54 (m, 4H), 7.65 (d, 1H, *J* = 7.3 Hz), 8.15 (s, 2H), 12.54 (s, 2H); ^13^C-NMR (101 MHz, DMSO-d6) *δ* ppm 111.7, 123.4, 123.7, 124.8, 128.1, 128.2, 130.2, 130.3, 130.7, 132.3, 132.9, 135.6, 151.4; HRMS (M + H)^+^ 244.0629 (calcd for C_13_H_10_ClN_3_H^+^ 244.0636).
∗ *4-(2,3-Dichlorophenyl)-1H-benzo[d]imidazol-2-amine Hydrobromide* (**49e**). Following general method H and starting from **47e** (122 mg, 0.48mmol), **49e** was obtained as an orange solid (53 mg, 0.15 mmol, 31%). Purity ≥ 98%; mp = 314–315 °C; ^1^H-NMR (400 MHz, DMSO-d6) *δ* ppm 7.14 (d, 1H, *J* = 7.9 Hz), 7.32 (t, 1H, *J* = 7.9 Hz), 7.43 (dd, 1H, *J* = 1.5 Hz, *J* = 7.9 Hz), 7.46 (d, 1H, *J* = 7.9 Hz), 7.51 (t, 1H, *J* = 7.9 Hz), 7.78 (dd, 1H, *J* = 1.5 Hz, *J* = 7.9 Hz), 8.24 (s, 2H), 12.55 (s, 1H), 12.61 (s, 1H); ^13^C-NMR (101 MHz, DMSO) *δ* ppm 112.1, 123.1, 123.8, 124.5, 128.2, 129.0, 130.3, 131.0, 131.1, 131.3, 132.7, 138.1, 151.4; HRMS (M + H)^+^ 278.0235 (calcd for C_13_H_9_Cl_2_N_3_H^+^ 278.0246).
∗ *4-(2,5-Dichlorophenyl)-1H-benzo[d]imidazol-2-amine Hydrobromide* (**49f**). Following general method H and starting from **47f** (80 mg, 0.32 mmol), **49f** was obtained as a white solid (70 mg, 0.19 mmol, 61%). Purity ≥ 98%; mp = 202–205 °C; ^1^H-NMR (400 MHz, DMSO-d6) *δ* ppm 7.15 (d, 1H, *J* = 7.8 Hz), 7.31 (t, 1H, *J* = 7.8 Hz), 7.45 (d, 1H, *J* = 7.8 Hz), 7.55–7.61 (m, 2H), 7.68 (d, 1H, *J* = 8.5 Hz), 8.26 (s, 2H), 12.65 (s, 2H); ^13^C-NMR (101 MHz, DMSO-d6) *δ* ppm 112.1, 122.0, 123.6, 124.7, 128.3, 130.4, 131.7, 131.8, 132.4, 137.4, 151.5; HRMS (M + H)^+^ 278.0240 (calcd for C_13_H_9_Cl_2_N_3_H^+^ 278.0246).
∗ *4-(2-Methoxyphenyl)-1H-benzo[d]imidazol-2-amine Hydrobromide* (**49g**). Following general method H and starting from **47g** (55 mg, 0.26 mmol), **49g** was obtained as an orange solid (44 mg, 0.14 mmol, 54%). Purity ≥ 95%; mp = 61–66 °C; ^1^H-NMR (400 MHz, DMSO-d6) *δ* ppm 3.77 (s, 3H), 7.09 (t, 1H, *J* = 7.4 Hz), 7.14 (d, 1H, *J* = 7.4 Hz), 7.19 (d, 1H, *J* = 7.9 Hz), 7.28 (t, 1H, *J* = 7.9 Hz), 7.33 (d, 1H, *J* = 7.4 Hz), 7.37 (d, 1H, *J* = 7.9 Hz), 7.47 (t, 1H, *J* = 7.4 Hz), 8.05 (s, 2H), 12.09 (s, 1H), 12.48 (s, 1H); ^13^C-NMR (101 MHz, DMSO) *δ* ppm 55.9, 110.9, 112.0, 121.1, 123.1, 123.7, 124.8, 125.3, 128.2, 129.9, 130.4, 131.3, 151.0, 156.7; HRMS (M + H)^+^ 240.1126 (calcd for C_14_H_13_N_3_OH^+^ 240.1131).

#### 4.1.10. Preparation of Dihydrobenzodiazepine **57**

∗ *(6-Bromo-2-nitrophenyl)ethanol* (**52**). Paraformaldehyde (114 mg, 3.80 mmol, 1.0 equiv.), 4-bromo-6-nitrotoluene **51** (2.00 g, 9.26 mmol, 2.44 equiv.) and Triton-B (114 µL, 40% in methanol) was dissolved in DMSO (2.0 mL). The resulting mixture was heated overnight at 90 °C. After it was cooled, the reaction mixture was diluted with a saturated solution of NH_4_Cl. The aqueous phase was extracted twice with EtOAc. The organic layers were combined, washed with brine, dried over Na_2_SO_4_, filtered, concentrated and purified by silica gel column chromatography (hexane:EtOAc 3:1 to 1:2), yielding to **52** as a white solid (720 mg, 2.93 mmol, 77%). ^1^H-NMR (300 MHz, CDCl_3_) δ ppm 3.31 (t, 2H, *J* = 6.9 Hz), 3.97 (t, 2H, *J* = 6.9 Hz), 7.26 (t, 1H, *J* = 7.8 Hz), 7.75 (d, 1H, *J* = 7.8 Hz), 7.83 (d, 1H, *J* = 7.8 Hz).
∗ *(2-Nitro-6-phenylphenyl)ethanol* (**53**). A microvawe vial under argon was charged with **52** (490 mg, 1.99 mmol, 1 equiv.), phenylboronic acid (267 mg, 2.19 mmol, 1.1 equiv.), Pd(OAc)_2_ (9 mg, 0.04 mmol, 20 mol%), K_2_CO_3_ (688 mg, 4.98 mmol, 2.5 equiv.) and TBAB (642 mg, 1.99 mmol, 1 equiv.) in water (2.2 mL). The vial was capped properly, flushed with argon and heated to 70°C for 3 h. After it was cooled, the reaction mixture was filtered through a pad of Celite^®^, washed with EtOAc, concentrated under vacuum and purified by silica gel column chromatography (hexane:EtOAc 2:1), yielding to **53** a white solid (441 mg, 1.81 mmol, 91%). ^1^H-NMR (300 MHz, CDCl_3_ ) δ ppm 1.39 (t, 1H, *J* = 5.9 Hz), 3.11 (t, 1H, *J* = 6.9 Hz), 3.64 (q, 1H, *J* = 6.5 Hz), 7.27–7.31 (m, 2H), 7.42–7.49 (m, 5H), 7.81 (d, 1H, *J* =5 7.5 Hz).
∗ *(2-Amino-6-phenylphenyl)ethanol* (**54**). Following general method H and starting from **53** (420 mg, 1.73 mmol), **54** was obtained as a purple solid (367 mg, 1.73 mmol, 100%). ^1^H-NMR (400 MHz, CDCl_3_) δ ppm 2.79 (t, 2H, *J* = 6.4 Hz), 3.75 (t, 2H, *J* = 6.4 Hz), 4.02 (br s, 2H), 6.70 (d, 1H, *J* = 7.6 Hz), 6.75 (d, 1H, *J* = 7.6 Hz), 7.10 (t, 1H, *J* = 7.6 Hz), 7.27–7.43 (m, 5H).
∗ *{2-[2,3-Di(tert-butoxycarbonyl)guanidino]-6-phenylphenyl}ethanol* (**55**). Following general method A and starting from **54** (100 mg, 0.47 mmol), **55** was obtained as a white solid (210 mg, 0.46 mmol, 98%). ^1^H-NMR (300 MHz, CDCl_3_) *δ* ppm 1.49 (s, 9H), 1.57 (s, 9H), 2.87 (t, 2H, *J* = 6.6 Hz), 3.61 (t, 2H, *J* = 6.6 Hz), 7.07 (d, 1H, *J* = 7.5 Hz), 7.26–7.44 (m, 6H), 7.76 (d, 1H, *J* = 7.5 Hz), 10.25 (s, 1H), 11.75 (s, 1H).
∗ *6-Phenyl-3,4-dihydrobenzodiazepin-2-amine Hydrochloride* (**57**). Compound **55** (105 mg, 0.23 mmol, 1 equiv.) was dissolved in THF (5.0 mL), and PPh_3_ (121 mg, 0.46 mmol, 2 equiv.) and DIAD (89.0 µL, 0.46 mmol, 2 equiv.) were added. The resulting mixture was stirred at rt for 1.5 h. The solution was concentrated under vacuum and purified by silica gel column chromatography (hexane:EtOAc 3:1). The intermediate **56** was diluted in a solution of HCl (4.0 N) in dioxane. The reaction mixture was stirred overnight at rt. The solution was concentrated under vacuum, yielding to **57** as a white solid (31 mg, 0.11 mmol, 49%). Purity ≥ 98 %; mp = 207–209 °C; ^1^H-NMR (500 MHz, DMSO-d6) δ ppm 3.21 (t, 2H, *J* = 8.1 Hz), 4.03 (t, 2H, *J* = 8.1 Hz), 7.16 (t, 1H, *J* = 4.6 Hz), 7.37–7.39 (m, 2H), 7.41 (m, 1H), 7.49 (m, 4H), 8.02 (br s, 4H); ^13^C-NMR (125 MHz, DMSO-d6) *δ* ppm 27.8, 51.2, 114.0, 124.9, 128.1, 128.5, 128.6, 129.1, 131.4, 139.2, 139.5, 141.3, 154.6; HRMS (M + H)^+^ 238.1331 (calcd for C_15_H_15_N_3_H^+^ 238.1339).

### 4.2. Biology

#### 4.2.1. High-Throughput Screening and Assays for Hit Validation

Primary screening for MSK1 inhibitors was performed on a fully robotized Beckman platform using the Homogeneous Time-Resolved Fluorescence (HTRF) technology (Cisbio Bioaasays, Codolet, France), using a purified active human kinase MSK1 (14-438-K, Millipore, Molsheim, France), and a substrate mimeticthe sequence surrounding the Ser276 of the p65 sub-unit of NF-κB (STK S3, 61ST3BLC, Cisbio Bioaasays, Codolet, France). Enzymatic reactions were performed for 30 min at room temperature in presence of 100 µM ATP, 1 µM of the substrate and biotin/streptavidin in a ratio of 2. Fluorescence was measured with EnVision Plate Reader (Perkin Elmer, Villebon-sur-Yvette, France). Assays were done into 384 well microplates (3824, Corning, Wiesbaden, Germany). The Z’ factor [20], a guaranty of quality, when it is > 0.5, was comprised between 0.5 and 1 in our assays.

An orthogonal luminescence-based assay Kinase-Glo^®^ (Promega, Madison, WI, USA) was used to validate the hits, quantifying the amount of the remaining ATP in the solution following the kinase reaction. Enzymatic reactions were performed up to 4h at room temperature in presence of 3 µM ATP. All compounds were tested in quadruplicates at 1 µM and 10 µM. Further, to determine IC_50_ values of best molecules, compound activities were determined at 8 concentrations ranging from 0.03 µM to 100 µM. Luminescence was measured with EnVision Plate Reader (Perkin Elmer).

#### 4.2.2. In Vitro Evaluation of the Effect of MSK1 Inhibitors

Primary human lung fibroblasts were cultured in DMEM/F-12 medium (Gibco Waltham, MA, USA) complemented with 10% fetal bovine serum (Gibco), penicillin (50 U/mL, Gibco), streptomycin (50 µg/mL, Gibco), L-glutamine (2 mM, Gibco), non-essential amino acids (0.1 mM, Gibco) and insulin (0.12 U/mL, Lilly, Indianapolis, IN, USA) Cells were used at passage 7. After attending confluence, cells were starved with medium containing 0.3% fetal bovine serum for 48 h. Cells were then treated with compounds at concentration range from 0.3 µM to 30 µM, incubated at 37 °C and 1h later cells were stimulated with IL-1β (20 U/mL, Roche, Meylan, France) for 5 h. Supernatant was then withdrawn and ELISA for IL-6 was realized following the manufacturer’s instructions (BD Opteia, San Jose, CA, USA).

#### 4.2.3. Cytotoxicity Test

A WST1 (Roche) reagent assay was used to determine the cytotoxicity of compounds at 24 h of incubation. Reagent was used at dilution 1:30 and incubation was done for 1.5 h at 37 °C with 5% CO_2_. The absorbance was measured at 450 nm.

#### 4.2.4. Evaluation of the In Vivo Toxicity of MSK1 Inhibitors

Selected compounds were injected by intraperitoneal (*i.p*.) route in 9-week old Balb/C mice (*n* = 2) at 10 and 30 mg/kg by unique injection. Mice were than observed closely for the next one hour, then every hour for 8h. Observations were continued for the next two days for signs of pain or toxicity such as change in the behavior and in the activity in the cage (decreased locomotion) or change in the appearance (curved back, eyes mi-closed, bristle hair, hips hollowed out). 

#### 4.2.5. Evaluation of Compound Activity in an Asthma Model in Mice

Animal experimentation was conducted in accordance with the European Communities Council Directive of 24 November 1986 (86/609/EEC) and the French ministry with the approval of the Regional Ethics Committee for animal research at the Strasbourg University (Authorization number 2015080309399690). Nine week-old male Balb/c mice (Janvier) were sensitized on days 0 and 7 by *i.p*. injection of ovalbumin (OVA, 50 μg, grade V, Sigma-Aldrich) adsorbed on aluminium hydroxide (2 mg, Sigma-Aldrich) in PBS. Mice were challenged intranasally (*i.n*.) with OVA (10 μg) in 25 μL of saline on days 18–21. Control mice received *i.n.* administrations of saline alone. Mice received compound (10 or 30 mg/kg) in saline solution with DMSO 5% or vehicle (saline, DMSO 5%) administered *i.p.* 2 h before each saline or OVA challenge. Collection of the bronchoalveolar lavage (BAL) fluid was performed 24 h after the last OVA challenge. Cell counts were assessed by flow cytometry (LSRII^®^ cytometer, BD bioscience). BAL cells were added with FCblock (5 μL, 553142, BD bioscience) in a black microplate, incubated for 20 min at room temperature. Then, marker antibodies were added: CD45-AlexaFluor700 (103128, BioLegend, San Diego, CA, USA), CD11b-APC-Cy7 (557657, BD bioscience), CD11c-FITC (557400, BD bioscience, Franklin Lakes, NJ, USA), Gr-1-Pe-eFluor610 (61-5931-82, eBioscience). Antibodies were incubated for 30 min before DAPI (5 μL, BD bioscience) addition, and flow cytometry was performed immediately. Eosinophils were selected by CD45^+^, CD11b^+^, CD11C^-^ and GR-1^-^ gating.

#### 4.2.6. Statistical Analysis

All data are expressed as means ± SEM. Statistical analyses were performed using a one-way ANOVA followed by Bonferroni’s multiple comparison post-test. Data were considered significantly different when *p* < 0.05.

## Figures and Tables

**Table 1:** Homologation and position of the phenyle on the pyridine scaffold.

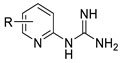			MSK1 ^a^		
			% Inhibition		IC_50_
Entry	Cpd	R	10 µM	1 µM	µM
1	**14**	3-Ph	11 ± 5	1 ± 5	nd
2	**13**	4-Ph	5 ± 1	−4 ± 1	nd
3	**12**	5-Ph	−11 ± 1	−10 ± 2	nd
4	**25**	5-(CH_2_)_2_Ph	6 ± 4	2 ± 4	nd
5	**1a**	6-Ph	42 ± 9	4 ± 4	17.9 ± 3.9
6	**17**	6-Bn	−2 ± 3	−2 ± 1	nd
7	**24**	6-(CH_2_)_2_Ph	−8 ± 1	−4 ± 1	nd
8	**H89**	-	93 ± 10	80 ± 8	0.25 ± 0.06
9	**Fasudil**	-	82 ± 10	50 ± 10	1.74 ± 0.16
10	**PHA767491**	-	94 ± 4	72 ± 5	0.62 ± 0.13

^a^ Standard Deviation (*n* = 4); nd = not determined.

**Table 2:** Effect of the aromatic substitution at position 6.

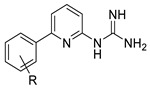			MSK1 ^a^		
			% Inhibition		IC_50_
Entry	Cpd	R	10 µM	1 µM	µM
1	**1a**	H	42 ± 9	4 ± 4	17.9 ± 3.9
2	**1b**	4-Cl	23 ± 1	−4 ± 5	nd
3	**1c**	4-CF_3_	−1 ± 5	−6 ± 2	nd
4	**1d**	4-OMe	−3 ± 4	−5 ± 3	nd
5	**1e**	3-Cl	45 ± 5	0 ± 4	9.7 ± 1.0
6	**1f**	3-OMe	16 ± 4	−7 ± 4	nd
7	**1j**	2-F	45 ± 9	6 ± 3	5.0 ± 0.5
8	**1k**	2-Cl	95 ± 8	62 ± 8	0.6 ± 0.1
9	**1l**	2-Me	48 ± 15	4 ± 8	3.5 ± 0.3
10	**1m**	2-CF_3_	38 ± 9	4 ± 2	8.1 ± 1.2
11	**1n**	2-OMe	66 ± 12	16 ± 3	2.3 ± 0.3
12	**1o**	2,3-Cl_2_	71 ± 13	7 ± 3	2.3 ± 0.3
13	**1p**	2,4-Cl_2_	31 ± 7	2 ± 2	ns
14	**1q**	2,5-Cl_2_	84 ± 4	23 ± 6	0.9 ± 0.1
15	**1i**	2-Cl, 3-CF_3_	62 ± 15	10 ± 5	8.6 ± 0.9

^a^**Fasudil** (MSK1, IC_50_ = 1.74 ± 0.16), **PHA767491** (MSK1, IC_50_ = 0.62 ± 0.13), **H89** (MSK1, IC_50_ = 0.25 ± 0.06) as positive controls; Standard Deviation (n = 4); ns = not significative, IC_50_ > 30 µM; nd = not determined.

**Table 3:** Replacement of the phenyl group by heterocycles.

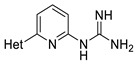			MSK1 ^a^		
			% inhibition	IC_50_	
Entry	Cpd	Het	10 µM	1 µM	µM
1	**1g**	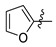	33 ± 8	4 ± 3	ns
2	**1h**	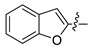	1 ± 6	−7 ± 5	nd
3	**1r**	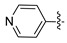	18 ± 2	1 ± 4	nd
4	**1s**	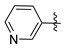	70 ± 7	9 ± 4	5.8 ± 0.6
5	**1t**	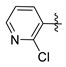	69 ± 11	10 ± 6	12.6 ± 4.0
6	**1u**	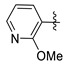	42 ± 7	3 ± 7	ns
7	**31**	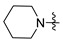	16 ± 4	−7 ± 4	nd

^a^**Fasudil** (MSK1, IC50 = 1.74 ± 0.16), **PHA767491** (MSK1, IC_50_ = 0.62 ± 0.13), **H89** (MSK1, IC_50_ = 0.25 ± 0.06) as positive controls; Standard Deviation (n = 4); ns = not significative, IC_50_ > 30 µM; nd = not determined.

**Table 4:** Dihydroquinazolines derivatives.

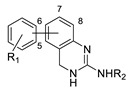					MSK1 ^a^		
					% Inhibition		IC_50_
Entry	Cpd	Position	R_1_	R_2_	10 µM	1 µM	µM
1	**37a**	5	H	H	44 ± 1	−19 ± 1	15.8 ± 3.2
2	**37b**	5	4-Cl	H	0 ± 7	−9 ± 2	nd
3	**37c**	5	4-OMe	H	−5 ± 5	−7 ± 4	nd
4	**37d**	5	3-Cl	H	10 ± 7	−5 ± 4	nd
5	**37e**	5	3-OMe	H	3 ± 7	−6 ± 4	nd
6	**37f**	5	2-Cl	H	38 ± 4	0 ± 2	16.4 ± 4.0
7	**37g**	5	2-OMe	H	−5 ± 1	−14 ± 3	nd
8	**60a**	5	H	*n*-Pr	2 ± 3	0 ± 1	nd
9	**60b**	5	H	Ph	1 ± 2	−2 ± 1	nd
10	**43**	6	H	H	9 ± 6	7 ± 9	nd

^a^**Fasudil** (MSK1, IC_50_ = 1.74 ± 0.16), **PHA767491** (MSK1, IC_50_ = 0.62 ± 0.13), **H89** (MSK1, IC_50_ = 0.25 ± 0.06), as positive controls; Standard Deviation (*n* = 4); ns = not significative, IC_50_ > 30 µM; nd = not determined.

**Table 5:** Benzimidazoles derivatives.

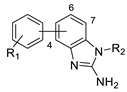					MSK1 ^a^		
					% Inhibition		IC_50_
Entry	Cpd	Position	R_1_	R_2_	10 µM	1 µM	µM
1	**49a**	4	H	H	46 ± 6	5 ± 3	3.6 ± 0.5
2	**49b**	4	4-Cl	H	49 ± 9	−3 ± 6	nd
3	**49c**	4	4-OMe	H	25 ± 5	−2 ± 6	nd
4	**49d**	4	2-Cl	H	71 ± 7	21 ± 5	1.6 ± 0.1
5	**49e**	4	2,3-Cl_2_	H	75 ± 8	20 ± 3	3.2 ± 0.2
6	**49f**	4	2,5-Cl_2_	H	61 ± 5	6 ± 3	6.8 ± 0.8
7	**49g**	4	2-OMe	H	39 ± 7	−3 ± 5	nd
8	**74a**	4	2-Cl	(CH_2_)_2_-Ph	41 ± 6	−6 ± 3	nd
9	**74b**	4	2-Cl	(CH_2_)_2_-OH	49 ± 13	−2 ± 4	nd
10	**74c**	4	2-Cl	(CH_2_)_3_-NHCOPh	37 ± 5	−4 ± 2	nd
11	**74d**	4	2-Cl	(CH_2_)_4_-Ph	5 ± 3	1 ± 3	nd
12	**50**	5	H	H	4 ± 4	−7 ± 5	nd
13	**75**	7	2-Cl	(CH_2_)_2_-Ph	1 ± 2	−6 ± 2	nd

^a^**Fasudil** (MSK1, IC_50_ = 1.74 ± 0.16), **PHA767491** (MSK1, IC_50_ = 0.62 ± 0.13), **H89** (MSK1, IC_50_ = 0.25 ± 0.06). as positive controls; Standard Deviation (n = 4); ns = not significative, IC_50_ > 30 µM; nd = not determined.

**Table 6:** Inhibition of IL-6 production by the selected compounds in an in vitro assay of human primary pulmonary fibroblasts in inflammatory conditions.

				Il-6 ^a^				
Entry	Group	R	Cpd	% Inhibition		IC_50_ (µM)	Cell Viability	
				10 µM	30 µM		10 µM	30 µM
1	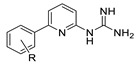	H	**1a**	17 ± 7	60 ± 4	16.3 ± 6.1	122 ± 14	103 ± 5
2		2-Cl	**1j**	42 ± 4	T	nd	100 ± 8	41 ± 9
3		2,5-Cl_2_	**1q**	T	T	nd	31 ± 19	13 ± 1
4	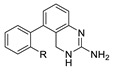	H	**37a**	18 ± 7	23 ± 6	ns	122 ± 14	103 ± 5
5		Cl	**37f**	17 ± 5	T	nd	100 ± 8	41 ± 9
6	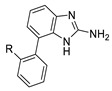	H	**49a**	12 ± 4	22 ± 1	ns	93 ± 17	91 ± 15
7		Cl	**49d**	39 ± 15	57 ± 2	13.9 ± 9.7	85 ± 13	76 ± 12
8	Reference compounds	**H89**		38 ± 5	96 ± 1	10.5 ± 5.1	96 ± 4	104 ± 3
		**Fasudil**		38 ± 6	81 ± 6	8.5 ± 5.1	95 ± 3	92 ± 3
		**PHA767491**		97 ± 1	98 ± 2	1.0 ± 0.1	95 ± 9	87 ± 7

^a^ Standard Deviation (*n* = 4); ns = not significative, IC_50_ > 30 µM; nd = not determined; T = toxic, cell viability < 75%.

## Data Availability

All data presented in this study are available in the article and in the Supplementary Material.

## Supplementary Materials

Supplementary Materials are available online.
