# Supplementary material for: Design, Synthesis and Biological Evaluation of Arylpyridin-2-yl Guanidine Derivatives and Cyclic Mimetics as Novel MSK1 Inhibitors. An Application in an Asthma Model
Source: Molecules. 2021 Jan 13;26(2):391. doi: 10.3390/molecules26020391 (PMC7828447; doi:10.3390/molecules26020391)
Supplement: Supplementary file 1 [file molecules-26-00391-s001.pdf]

Article

Design, Synthesis and Biological Evaluation of Arylpyridin-2-yl Guanidine Derivatives and Cyclic Mimetics as Novel MSK1 Inhibitors. An Application in an Asthma Model

Maud Bollenbach [1,2,†], Simona Nemska [1,2,†], Patrick Wagner [1,2], Guillaume Camelin [1], François Daubeuf [1,2,3],
Adeline Obrecht [2,3], Pascal Villa [2,3], Didier Rognan [1,2], Frédéric Bihel [1,2], Jean-Jacques Bourguignon [1],
Martine Schmitt [1,2,*] and Nelly Frossard [1,2,*]

1 Laboratoire d'Innovation Thérapeutique UMR 7200, LabEx Medalis, CNRS, Faculté de Pharmacie, Université de Strasbourg, Illkirch, France; maudbollenbach@gmail.com (M.B.); simona.nemska@humanitasresearch.it (S.N.); pwagner@unistra.fr (P.W.); camelin.guillaume@gmail.com (G.C.); daubeuf@unistra.fr (F.D.); drognan@unistra.fr (D.R.) ; fbihel@unistra.fr (F.B.); jjb@unistra.fr (J.-J.B.)
2 Labex MEDALIS, F-67000 Strasbourg, France; aobrecht@unistra.fr (A.O.); pvilla@unistra.fr (P.V.)
3 CNRS, Université de Strasbourg, PCBIS Plate-forme de Chimie Biologique Intégrative de Strasbourg UMS 3286, F-67412 Illkirch, France
* Correspondence: mschmitt@unistra.fr (M.S.); nelly.frossard@unistra.fr (N.F.); Tel.: +33-3-6885-4231 (M.S.)

Content

General Methods	p.2
*1-Preparation of 1-[6-(Piperidin-1-yl)pyridin-2-yl]guanidine Trifluoroacetate (**31**)*	p.3-4
*2-Preparation of 2-thiourea-6-phenylpyridine (**33**)*	p.4
*3-Preparation of N-substitured quinazolines (**60**)*	p.5-6
*4- Preparation of 4-Aryl dihydrobenzoimidazolone (**64**) and 4-aryl-2 amino benzothiazole (**67**)*	p.6-7
*5- Preparation of N1-alkyl-2-amino benzimidazoles (**74**) and (**75**)[a].*	p.7-10

General methods:

General Method A: Preparation of Boc-Protected guanidines derivatives

A solution of appropriate 2-amino pyridine derivatives (1.0 equiv.), N,N'-bis-(tert-butoxycarbonyl)-S-methylisothiourea (0.95 equiv.), triethylamine (4.4 equiv.) and mercury chloride (1.1 equiv.) in CH_2Cl_2 (3.2 mL / mmol) were stirred at rt overnight. After completion of the reaction, the reaction mixture was filtered through a pad of Celite® with CH_2Cl_2 as the *washing* solvent. The filtrate was concentrated under vacuum and purified by silica gel column chromatography, eluting with the appropriate hexane:EtOAc mixture.

General method B: *Pd-Catalyzed Suzuki-Miyaura cross-coupling using $Pd(PPh_3)_4$. Preparation of* ***64-65, 69 (see SI).***

A microwave vial under argon was charged with the corresponding halogeno derivative (1.0 equiv.), the corresponding phenylboronic acid (1.2 equiv.), $Pd(PPh_3)_4$ (5 mol%), Na_2CO_3 (3.0 equiv.) and a mixture toluene:ethanol:water (5:1:1, 5.32 mL /mmol)). The vial was capped properly, flushed with argon and heated to 120°C until complete conversion of the starting material. After it was cooled, the reaction mixture was concentrated under vacuum. The crude residue was diluted in water. The organic phase was extracted 3 times with EtOAc. The organic layers were combined, washed with brine, dried over Na_2SO_4, filtered, concentrated and purified by silica gel column chromatography, eluting with the appropriate hexane:EtOAc mixture.

General Method E*: N-BOC removal with trifluoroacetic acid*

The appropriate di- BOCprotected guanidine (1 equiv.) was dissolved in a mixture of TFA:DCM (1:1; 8 ml / mmol). The solution was stirred at room temperature for 2 hours. The solution was concentrated under vacuum and purified by reverse phase C18 column chromatography ($MeOH/H_2O$ + 0.05%TFA).

General method H*. Cyclisation of a diamino derivatives* ***72*** *and* ***73*** *using BrCN: Preparation of N1-alkyl-2-amino benzimidazoles* ***74*** *and* ***75***

Appropriate benzene-1,2 diamine derivative **72** or **73** (1 equiv.) was dissolved in toluene (1.5 mL/ mmol), followed by dropwise addition of a solution of BrCN (1.5 equiv.) in toluene (1 mL/mmol). The resulting solution was heated at 110 °C for 4 hours. After it was cooled, the solution was concentrated under vacuum and immediately purified by reverse C18 phase chromatography ($MeOH/H_2O$ + 0.05%HBr) to afford N-alkyl 2-amino benzimidazoles **74** and **75**.

1. Preparation of 1-[6-(Piperidin-1-yl)pyridin-2-yl]guanidine Trifluoroacetate (**31**)[a].

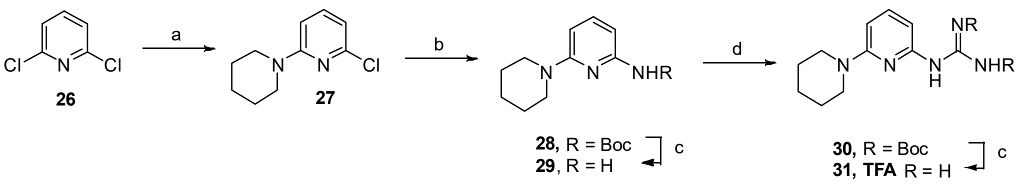

[a]Reaction conditions : (a) Piperidine, K_3PO_4, dioxane, 105°C, 36h, 81%; (b) NH_2Boc, $Pd(OAc)_2$, XantPhos, Cs_2CO_3, dioxane, 70°C, 4h, 83%; (c) TFA:DCM (1:1), rt, 2h, 82–94%; (d) N,N′-diBoc-S-methylisothiourea, $HgCl_2$, NEt_3, DCM, rt, 16h, 57%.

* *2-Chloro-6-(piperidin-1-yl)pyridine (*27*).* 2,6-Dichloropyridine **26** (150 mg, 1.01 mmol, 1 equiv.), piperidine (120.1 µL, 1.22 mmol, 1.2 equiv.) and K_3PO_4 (861 mg, 4.05 mmol, 4 equiv.) were dissolved in dioxane (3.0 mL). The reaction media was heated at 105°C for 36 hours. After it was cooled, the reaction mixture was concentrated under vacuum. The crude residue was diluted in water. The organic phase was extracted 3 times with EtOAc. The organic layers were combined, washed with brine, dried over Na_2SO_4, filtered, concentrated and purified by silica gel column chromatography (hexane:EtOAc 95:5), yielding to **27** as a colorless oil (162 mg, 0.82 mmol, 81%). ^{1}H NMR (400 MHz, $CDCl_3$) δ ppm 1.59–1.65 (m, 6H), 3.50–3.53 (m, 4H), 6.47 (d, 1H, *J* = 8.4 Hz), 6.51 (d, 1H, *J* = 7.4 Hz), 7.34 (dd, 1H, *J* = 7.4 Hz, *J* = 8.4 Hz); ^{13}C NMR (101 MHz, $CDCl_3$) δ ppm 24.6, 25.4, 46.0, 104.4, 111.1, 139.5, 149.4, 159.3. Analytical data are consistent with the previously reported characterization [1] .
* *tert-Butyl N-[6-(piperidin-1-yl)pyridine-2-yl]carbamate (*28*).* A microvawe vial (oven-dried and under argon) was charged with **27** (132 mg, 0.67 mmol, 1.0 equiv.), *tert*-butyl carbamate (86 mg, 0.74 mmol, 1.1 equiv.), $Pd(OAc)_2$ (4.5 mg, 0.02 mmol, 3 mol%), XantPhos (23 mg, 0.04 mmol, 6 mol%), Cs_2CO_3 (328 mg, 1.01 mmol, 1.5 equiv.) and anhydrous dioxane. The vial was capped properly, flushed with argon and heated to 70°C for 4 hours. After it was cooled, the reaction mixture was concentrated under vacuum. The crude residue was diluted in water. The organic phase was extracted 3 times with EtOAc. The organic layers were combined, washed with brine, dried over Na_2SO_4, filtered, concentrated and purified by silica gel column chromatography (hexane:EtOAc 95:5), yielding to **28** as a colorless oil (154 mg, 0.55 mmol, 83%). ^{1}H NMR (400 MHz, $CDCl_3$) δ ppm 1.51 (s, 18H), 1.58–1.62 (m, 6H), 3.44–3.47 (m, 4H), 6.29 (d, 1H, *J* = 8.3 Hz), 6.90 (s, 1H), 7.12 (d, 1H, *J* = 7.8 Hz), 7.42 (dd, 1H, *J* = 7.8 Hz, *J* = 8.3 Hz); ^{13}C NMR (101 MHz, $CDCl_3$) δ ppm 24.8, 25.4, 28.3, 46.1, 80.4, 99.8, 101.4, 139.5, 150.0, 152.3, 158.4.
* *6-(Piperidin-1-yl)pyridin-2-amino Trifluoroacetate (*29*).* Following general method E and starting from **28** (138 mg, 0.50 mmol), **29** was obtained as a colorless oil (137 mg, 0.47 mmol, 94%). ^{1}H NMR (400 MHz, DMSO) *δ* ppm 1.57–1.62 (m, 6H), 3.43–3.46 (m, 4H), 6.05 (d, 1H, *J* = 8.3 Hz), 6.18 (d, 1H, *J* = 8.3 Hz), 7.41 (br s, 2H), 7.59 (t, 1H, *J* = 8.3 Hz); ^{13}C NMR (101 MHz, DMSO) δ ppm 23.8, 25.1, 47.9, 95.7, 97.1, 145.3, 151.4, 153.8.
* *1-[2,3-Di(*tert*-butoxycarbonyl)guanidino]-6-(piperidin-1-yl)pyridine (*30*).* Following general method A and starting from **29** (118 mg, 0.40 mmol) and 5.4 equiv. of NEt_3**,** **30** was obtained as a white solid (92 mg, 0.22 mmol, 57%). ^{1}H NMR (400 MHz, $CDCl_3$) *δ* ppm 1.52 (s, 18H), 1.60–1.63 (m, 6H), 3.54–3.49 (m, 4H), 6.36 (d, 1H, *J* = 8.5 Hz), 7.45 (t, 1H, *J* = 8.5 Hz), 7.62 (br s, 1H), 10.43 (s, 1H), 11.56 (s, 1H); ^{13}C NMR (101 MHz, $CDCl_3$) *δ* ppm 24.7, 25.5, 28.2, 102.9, 139.5.
* *1-[6-(Piperidin-1-yl)pyridin-2-yl]guanidine Trifluoroacetate (*31*).* Following general method E and starting from **30** (49 mg, 0.12 mmol), **31** was obtained as a white solid (32 mg, 0.10 mmol, 82%). Purity ≥ 98%; mp = 196–197°C; ^{1}H NMR (400 MHz, DMSO-d6) δ ppm 1.52–1.63 (m, 6H), 3.40–3.43 (m, 4H), 6.24 (d, 1H, *J* = 7.7 Hz), 6.58 (d, 1H, *J* = 8.5 Hz), 7.55 (dd, 1H, *J* = 7.7 Hz, *J* = 8.5 Hz), 8.36 (br s, 4H), 10.93 (s, 1H); ^{13}C NMR (101 MHz, DMSO) δ ppm 24.4, 25.3, 46.6, 100.7, 103.0, 140.8, 150.7, 155.7, 157.9, 159.7 (q, *J* = 33.0 Hz); HRMS $(M + H)^+$ 220.1549 (calcd for $C_{11}H_{17}N_5H^+$ 220.1557).

2. Preparation of 2-thiourea-6-phenylpyridine (**33**)[a].

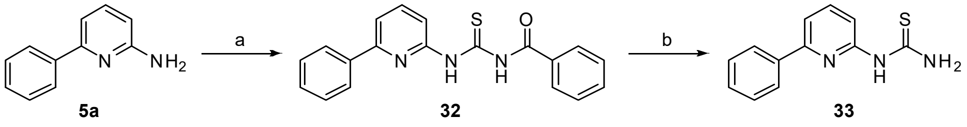

[a]Reaction conditions : (a) Benzoyl isothiocyanate, THF, 70°C, 14h, 97%; (b) NaOH, EtOH:H_2O (1:1), 80°C, 1.5h, 36%.

* *1-Benzoyl-3-(6-phenylpyridin-2-yl)thiourea (***32***).* **5a** (217 mg, 1.28 mmol, 1 equiv.) and benzoyl isothiocyanate (189 μL, 1.40 mmol, 1.1 equiv.) were dissolved in THF (10.5 mL). The resulting mixture was heated at reflux for 14 hours. After it was cooled, the reaction mixture was concentrated under vacuum and purified by silica gel column chromatography (hexane:EtOAc 9:1 to 7:3), yielding to **32** as a yellow solid (413 mg, 1.24 mmol, 97%). ^1H NMR (400 MHz, $CDCl_3$) δ ppm 7.40–7.50 (m, 3H), 7.52–7.58 (m, 2H), 7.63–7.68 (m, 2H), 7.85 (t, 1H, *J* = 8.0 Hz), 7.93 (d, 2H, *J* = 7.3 Hz), 8.07 (d, 2H, *J* = 7.3 Hz), 8.79 (d, 1H, *J* = 8.0 Hz), 9.08 (s, 1H), 13.15 (s, 1H); ^{13}C NMR (101 MHz, $CDCl_3$) δ ppm 114.3, 117.9, 126.9, 127.6, 128.7, 129.3, 129.4, 131.7, 133.8, 138.1, 138.5, 151.0, 156.2, 166.4, 176.9.
* *(6-Phenylpyridin-2-yl)thiourea (***33***).* **32** (258 mg, 0.77 mmol, 1 equiv.) was dissolved in ethanol (5.1 mL). NaOH (93 mg, 2.32 mmol, 3 equiv.) was dissolved in water (5.1 mL) and added in the previous solution. The resulting solution was heated at reflux for 1.5 h. After it was cooled, the reaction mixture was concentrated under vacuum. The crude residue was diluted in water. The organic phase was extracted 3 times with EtOAc. The organic layers were combined, washed with brine, dried over Na_2SO_4, filtered, concentrated and purified by silica gel column chromatography (hexane:EtOAc 4:1), yielding to **33** as a white solid (63 mg, 0.28 mmol, 36%). Purity ≥ 98%; mp = 191–193 °C; ^1H NMR (400 MHz, DMSO) δ ppm 7.16 (d, 1H, *J* = 8.2 Hz), 7.43–7.53 (m, 3H), 7.57 (d, 1H, *J* = 7.5 Hz), 7.83–7.88 (m, 3H), 8.98 (s, 1H), 10.65 (s, 1H), 10.72 (s, 1H); ^{13}C NMR (101 MHz, DMSO-d6) δ ppm 112.1, 115.3, 126.8, 129.5, 129.9, 138.6, 140.4, 154.1, 181.2; HRMS $(M + H)^+$ 230.0741 (calcd for $C_{12}H_{11}N_3SH^+$ 230.0746).

3. Preparation of N-substitured quinazolines (**60**)[a]

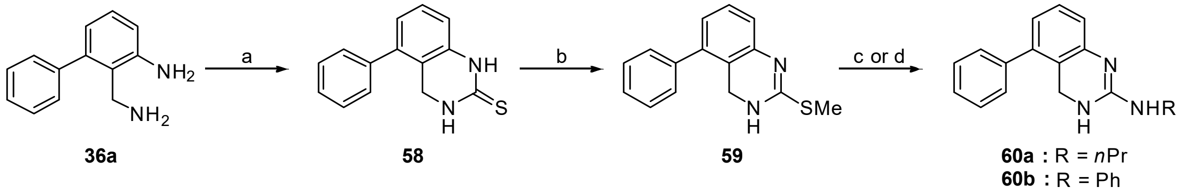

[a]Reaction conditions : (a) $CSCl_2$, NEt_3, Et_2O, −78°C to rt, 10h, 72%; (b) MeI, acetone, rt, 12h, 90%, (c) *for R = nPr* : propylamine, 110 °C, 0.5 h, μW, 31%; (d) *for R = Ph* : aniline, 160 °C, 3 h, 19%.

* *5-Phenyl-1,2,3,4-tetrahydroquinazoline-2-thione (***58***).* **36a** (80 mg, 0.40 mmol, 1 equiv.) was dissolved in diethylic ether (3.0 mL) and NEt_3 (227 μL, 1.62 mmol, 4 equiv.) was added. The resulting solution was cooled at −78°C and a solution of $CSCl_2$ (43 μL, 0.57, 1.4 equiv.) in diethylic ether (1.0 mL) was added dropwise. The reaction mixture was allowed to come back to rt and stirred overnight. The reaction media was diluted using methanol, concentrated under vacuum and purified by reverse chromatography (MeOH/H_2O + 0.05%TFA), yielding to **58** as a white solid (70 mg, 0.29 mmol, 72%). ^1H NMR (400 MHz, DMSO) δ ppm 4.23 (s, 2H), 6.94 (d, 1H, *J* = 8.0 Hz), 7.02 (d, 1H, *J* = 8.0 Hz), 7.28 (t, 1H, *J* = 8.0 Hz), 7.32 (d, 2H, *J* = 7.2 Hz), 7.41–7.49 (m, 3H).
* *2-(Methylsulfanyl)-5-phenyl-3,4-dihydroquinazolinium Iodide (***59***).* **58** (40 mg, 0.12 mmol, 1 equiv.) and MeI (23.3 μL, 0.37 mmol, 3 equiv.) were dissolved in acetone (3.0 mL). The resulting mixture was stirred overnight at rt. The solution was concentrated under vacuum, triturated 3 times with ether and filtered, yielding to **59** as a white solid (43 mg, 0.11 mmol, 90%). ^1H NMR (400 MHz,

DMSO) δ ppm 2.73 (s, 3H), 4.56 (s, 2H), 7.15 (d, 1H, *J* = 8.0 Hz), 7.22 (d, 1H, *J* = 8.0 Hz), 7.36 (d, 2H, *J* = 8.0 Hz), 7.44–7.52 (m, 4H), 10.06 (br s, 1H), 12.15 (br s, 1H).

* *5-Phenyl-N-propyl-3,4-dihydroquinazolin-2-amine Trifluoroacetate (**60a**).* **59** (48 mg, 0.12 mmol, 1 equiv.) was dissolved in propylamine (2.0 mL), and the resulting mixture was microvawe heated at 110°C for 30 minutes. After it was cooled, the solution was concentrated and purified by reverse chromatography (MeOH/H_2O + 0.05%TFA), yielding to **60a** as an orange solid (15 mg, 0.04 mmol, 31%). Purity ≥ 95 %; mp = 70–72 °C; ^{1}H NMR (500 MHz, DMSO) δ ppm 0.88–0.92 (m, 3H), 1.55 (q, 2H, *J* = 7.2 Hz), 3.21 (br s, 2H), 4.35 (s, 2H), 7.06 (d, 1H, *J* = 7.6 Hz), 7.24 (d, 2H, *J* = 7.2 Hz), 7.38 (d, 1H, *J* = 7.6 Hz), 7.43 (t, 1H, *J* = 7.2 Hz), 7.48 (t, 2H, *J* = 7.2 Hz), 7.76 (br s, 1H), 8.50 (br s, 1H), 8.68 (br s, 1H), 11.12 (br s, 1H); ^{13}C NMR (125 MHz, DMSO) δ ppm 11.4, 20.9, 22.2, 43.2, 125.7, 128.3, 128.9, 129.0, 129.1, 129.2, 134.7, 138.9, 139.9, 152.3; ; HRMS (M + H)$^+$ 266.1649 (calcd for $C_{17}H_{19}N_3H^+$ 266.1652).
* *N,5-Diphenyl-3,4-dihydroquinazolin-2-amine Trifluoroacetate (**60b**).* **59** (48 mg, 0.12 mmol, 1 equiv.) was dissolved in aniline (0.5 mL), and the resulting mixture was heated at 160°C for 3 hours. After it was cooled, the solution was concentrated and purified by reverse chromatography (MeOH/H_2O+0.05%TFA), yielding to **60b** as an orange solid (10 mg, 0.02 mmol, 19%). Purity ≥ 98 %; mp = 77–82 °C; ^{1}H NMR (500 MHz, DMSO) δ ppm 4.41 (s, 2H), 7.13 (t, 2H, *J* = 7.6 Hz), 7.30–7.35 (m, 5H), 7.39–7.51 (m, 6H), 8.70 (s, 1H), 10.77 (s, 1H), 11.49 (s, 1H); ^{13}C NMR (125 MHz, DMSO) δ ppm 40.6, 115.8, 117.3, 124.5, 126.3, 127.0, 128.3, 129.0, 129.1, 130.3, 134.4, 135.7, 138.8, 140.0, 151.1; HRMS (M + H)$^+$ 300.1492 (calcd for $C_{20}H_{17}N_3H^+$ 300.1495).

4. Preparation of 4-Aryl dihydrobenzoimidazolone (**64**) and 4-aryl-2 amino benzothiazole (**67**)[a].

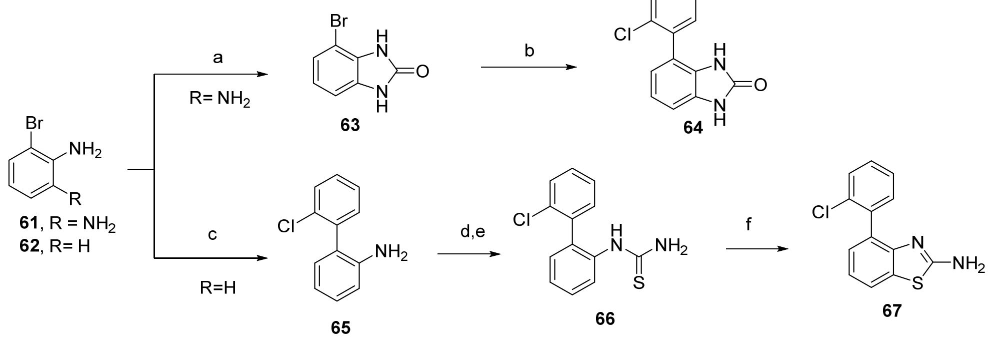

[a]Reaction conditions : (a) CDI, THF, 50°C, 18h, 71%; (b) $Pd(PPh_3)_4$, Na_2CO_3, Tol:EtOH:H_2O (5:1:1), 110°C, 16h, 87%; (d) Pd(dppf)Cl_2.DCM, K_2CO_3, DME: H_2O (4:1), 80 °C, 2 h, 31%; (e) PhCON=C=S, THF, rfx, 14 h, 81%; (d) NaOH, EtOH:H_2O (1:1), 80 °C, 1.5 h, 94%; (f) Br_2, DCM, 40 °C, 2 h, 42%.

* *4-bromo-1 ,3-dihydro- benzoimidazol-2-one* (**63**) To a solution of commercially available 3-bromo-benzene-1,2-diamine (300 mg, 1.6 mmol, 1 equiv.) in THF (3 mL) was added 1,1'-carbonyldiimidazole (520.2 mg, 3.21 mmol, 2 equiv) and the resulting mixture was heated at 50° C overnight. The solvent was evaporated, water (20 mL) was added and the product was extracted with EtOAc (3 x, 15 mL). The combined organic extracts were dried over Na_2SO_4 and the solvent removed, followed by silica gel chromatography to provide **63** (243 mg, 71%). ^{1}H NMR (400 MHz, DMSO-d6) δ 6.90 (dt, *J* = 15.5, 7.6 Hz, 2H), 7.11 (d, *J* = 7.8 Hz, 1H), 10.91 (s, 1H), 11.03 (s, 1H).
* *4-(2-chlorophenyl)-2,3-dihydro-1H-1,3-benzodiazol-2-one (**64**)* Following general method B and starting from 4-bromo-2,3-dihydro-1H-1,3-benzodiazol-2-one 63 (120 mg, 0.563 mmol, 1 equiv.) and 2-chlorophenyl boronic acid (105.7 mg, 0.67 mmol, 1.2 equiv.) the title compound was obtained after 2 h at 120°C as a white solid 119.4 mg, 0.49 mmol, 87%). ^{1}H NMR (400 MHz, DMSO-d6) δ 6.81 (d, *J* = 7.4 Hz, 1H), 6.99 (dt, *J* = 14.9, 7.6 Hz, 2H), 7.37–7.44 (m, 3H), 7.49–7.65 (m, 1H), 10.52 (s, 1H), 10.72 (s, 1H). ^{13}C NMR (101 MHz, DMSO) δ 108.4, 120.7, 120.9, 122.2, 127.7, 128.4, 129.9, 130.1, 130.2, 132.2, 133.0, 136.8, 155.8. HRMS (M + H)$^+$ 245.0471 (calcd for $C_{13}H_9ClN_2OH^+$ 245.0482).

* *2-(2-chlorophenyl)aniline* (***65***) Following general method B and starting from 2 bromo-aniline (516, 1 mg, 3 mmol, 1 equiv.) and 2-chlorophenyl boronic acid (563. Mg, 3.6 mmol, 1.2 equiv.), the title compound was obtained after 5 h at 120 °C as a white solid (374, 6 mg, 1.84 mmol, 61%). The spectral data matched that reported by Stokes and and coworkers [2].

* *[2-(2-chlorophenyl)phenyl]thiourea (**66**)* 2-(2-chlorophenyl)aniline **65** (225 mg, 1.11 mmol, 1 equiv.) was dissolved in THF (9.1 mL) followed by benzoyl isothiocyanate (198 mg, 0.164 mL, 1.22 mmol, 1.1 equiv.) and the resulting mixture was heated at reflux for 14 hours. After it was cooled, the reaction mixture was concentrated under vacuum and purified by silica gel column chromatography (hexane:EtOAc 9:1 to 7:3) to yield 1-benzoyl-3-[2-(2-chlorophenyl)phenyl]thiourea (330.7 mg, 0.90 mmol, 81%). 1H NMR (400 MHz, $CDCl_3$) δ 7.10–7.15 (m, , 3H), 7.14–7.22 (m, 2H), 7.23–7.31 (m, 4H), 7.38 (t, *J* = 7.3 Hz, 1H), 7.54 (d, *J* = 7.9 Hz, 2H), 7.85 (d, *J* = 8.0 Hz, 1H), 8.70 (s, 1H), 11.88 (s, 1H).

* 1-benzoyl-3-[2-(2-chlorophenyl)phenyl]thiourea (306 mg, 0.83 mmol, 1 equiv.) was dissolved in ethanol (5.0 mL). NaOH (100 mg, 2.50 mmol, 3 equiv.) was dissolved in water (5.0 mL) and added in the previous solution. The resulting solution was heated at reflux for 1.5 hours. After it was cooled, the prepicipated was filtered, washed with water and dried. The title compound was obtained as a white solid as a white solid (262.8mg, 0.79 mmol, 94%). ^{1}H NMR (400 MHz, DMSO-d6 + D_2O) δ 7.26 (d, *J* = 7.6 Hz, 1H), 7.32 (t, *J* = 7.4 Hz, 1H), 7.37–7.45 (m, 4H), 7.56 (d, *J* = 7.3 Hz, 2H), 8.85 (s, 1H).^{13}C NMR (101 MHz, DMSO-d6) δ 126.4, 127.4, 128.7, 129.0, 129.8, 129.9, 131.1, 132.3, 132.9, 135.6, 137.2, 137.6, 137.7.

* *4-(2-chlorophenyl)-1,3-benzothiazol-2-amine trifluoroacetate (**67**)* A solution of bromine (78 μL, 1.52 mmol, 2 equiv.) in CH_2Cl_2 (0.33 mL) was added dropwise to a stirred suspension of [2-(2-chlorophenyl)phenyl]thiourea **66** (199.4 mg, 0.76 mmol, 1 quiv.) in CH_2Cl_2 (2.3 mL). The reaction mixture was refluxed for 2.0 hours and allowed to stand at room temperature for 12 hours. The residue was treated with dilute ammonium hydroxide solution and the aqueous phase was extracted twice with EtOAc. The combined organic extracts were dried over Na_2SO_4 and the solvent removed, followed by reverse phase chromatography to provide the title compound **67** as a trifluoroacetate salt (119.5 mg, 0.32 mmol, 42 %). ^{1}H NMR (400 MHz, DMSO-d6) δ 7.10–7.16 (m, 2H), 7.35–7.42 (m, 3H), 7.50–7.56 (m, 1H), 7.74 (t, *J* = 4 Hz, 1H), 7.79 (bs, 2H). HRMS $(M + H)^+$ 261.0276 (calcd for $C_{13}H_9ClN_2SH^+$ 261.0253).

5. Preparation of N1-alkyl-2-amino benzimidazoles (**74**) and (**75**)[a].

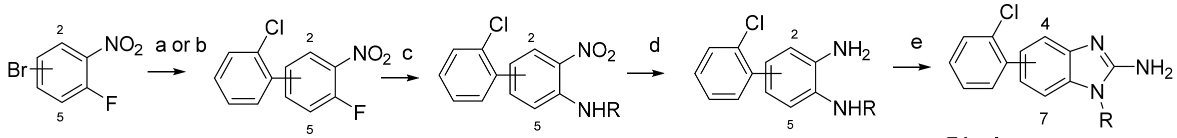

68a: 2-Br
68b, 5-Br

69a : 2-substituted
69b : 5-substituted

70a-d : 2-substituted
71 : 5-substituted

72a-d : 2-substituted
73 : 5-substituted

74a-d : 4-substituted
75 :7-substituted

[a]Reaction conditions : (a) 2-Br: $Pd(PPh_3)_4$, Na_2CO_3, Tol:EtOH:H_2O (5:1:1), 110 °C, 2 h, 94%; (b) 5-Br: $Pd(dppf)Cl_2$ [3] , K_2CO_3, DME: H_2O (4:1), 80 °C, 12 h, 80% (c) RNH_2, DIEA, DMF, 25 °C, 54–74%; (d) Sn, HCl, EtOH, 2 h, rfx then NaOH, 30–53%; (e) BrCN, MeCN:MeOH (1:1), 80 °C, 3 h, 25–77%

* *1-(2-chlorophenyl)-3-fluoro-2-nitrobenzene (**69a**)* Following general method B and starting from 1-bromo-3-fluoro-2-nitrobenzene **68a** (200 mg, 0.91 mmol) and 2-chlorophenylboronic acid (170.6 mg, 1.09 mmol, 1.2 equiv.) the title compound **68a** was obtained (141.8 mg, 0.56 mmol, 62%) and used directly in the SNAr reaction. ^{1}H NMR (400 MHz, CDC13) δ 7.13 (d, *J* = 7.7Hz, 1H), 7.18–7.33 (m, 4H), 7.41 (d, *J* = 7.8 Hz, 1H), 7.47–7.52 (m, 1H). ^{19}F NMR (376 MHz, $CDCl_3$) δ −122.15.

* *1-(2-chlorophenyl)-2-fluoro-3-nitrobenzene* (**69b**) Following general method B and starting from 1-bromo-2-fluoro-3-nitrobenzene **68b** (200 mg, 0.91 mmol) and 2-chlorophenylboronic acid (170.6 mg, 1.09 mmol, 1.2 equiv.) the title compound **68b** (70 mg, 0.28 mmol, 31%) was obtained and used directly in the SNAr reaction. ^{1}H NMR (400 MHz, MeOD) δ 7.34–7.48 (m, 4H), 7.51–7.56 (m, 1H), 7.64 (ddd, *J* = 7.8, 6.2, 1.8 Hz, 1H), 8.11 (ddd, *J* = 8.5, 6.9, 1.8 Hz, 1H).

* *3-(2-chlorophenyl)-1-N-(2-phenylethyl)benzene-1,2-diamine (**72a**)* To a solution of 1-(2-chlorophenyl)-3-fluoro-2-nitrobenzene (230 mg, 0.91 mmol, 1 equiv.) in DMF (4.3 mL) were added phenethylamine (121, 8 mg, 127 μL, 1.0 mmol, 1.1 equiv.) and DIPEA (177.2 mg, 227 μL, 1.37 mmol, 1.5 equiv.). The reaction mixture was stirred overnight at rt. After the completion of the reaction (monitored by TLC), the reaction mixture was

evaporated to dryness, the residue was diluted with water and extracted with EtOAc (3 x 20 mL). The combined organic layer was dried over Na_2SO_4 and concentrated under reduced pressure, and the crude material was purified by flash chromatography (10% EtOAc/hexanes) to afford 3-(2-chlorophenyl)-2-nitro-N-(2-phenylethyl)aniline **70a** which was used directly in the reduction step.

To a stirred ice cooled solution of **70a** (225 mg, 0.64 mmol, 1 equiv.) in EtOH (1.38 mL) was added fine granulated Tin (221, 8mg, 1.87 mmol, 2.93 equiv.). 12N HCl (693 µL) was slowly added while continuing the magnetic stirring. After the addition was completed, the reaction the resulting mixture was refluxed for 2h. After cooling the reaction mixture was then dilute with H_2O and pellets of KOH was added with care until pH reached 14. The suspension was then filtered through a pad of celite and the filtrate was extracted twice with EtOAc. The combined organic phase was dried over Na_2SO_4, filtered and concentrated in vacuo. Compound was purified by chromatography on silica gel using a gradient of 20 to 80% EtOAc in Heptane to afford **72a** as an oil. ^{1}H NMR (400 MHz, $CDCl_3$) δ 2.94 (t, *J* = 7.1 Hz, 2H), 3.17 (bs, 2H), 3.39 (dq, *J* = 13.7, 6.9 Hz, 2H), 6.55 (d, *J* = 7.5 Hz, 1H), 6.70 (d, *J* = 7.9 Hz, 1H), 6.82 (t, *J* = 7.8 Hz, 1H), 7.15–7.29 (m, 8H), 7.40–7.47 (m, 1H). ^{13}C NMR (101 MHz, $CDCl_3$) δ 35.9, 45.6, 103.1, 111.4, 113.2, 119.8, 120.1, 122.5, 122.7, 126.4, 127.1, 128.6, 128.8, 129.0, 129.8, 132.0, 137.6, 139.5.

* *2-{[2-amino-3-(2-chlorophenyl)phenyl]amino}ethan-1-ol* (**72b**). Using the same procedure described for **72a** and starting from 1 -(2-chlorophenyl)-3-fluoro-2-nitrobenzene (120 mg, 0.48 mmol, 1 equiv.) and ethanolamine (34.9 mg, 34.5 µL, 1.0 mmol, 1.2 equiv.), 2-{[3-(2-chlorophenyl)-2-nitrophenyl]amino}ethan-1-ol, **70b** was isolated as an oil. ^{1}H NMR (400 MHz, $CDCl_3$) δ 1.74 (bs, 1H) 3.31–3.58 (m, 2H), 3.93 (t, *J* = 5.4 Hz, 2H), 6.56 (dd, *J* = 7.4, 1.3 Hz, 1H), 6.93 (dd, *J* = 8.7, 1.3 Hz, 1H), 7.06 (bs, 1H), 7.19–7.34 (m, 4H), 7.36–7.46 (m, 2H). Following the reduction step with Sn/HCl described for **70a** and starting from **70b** (76.8 mg, 0.26 mmol, 1 equiv.), the title compound was obtained as an oil (43.5 mg, 0.16 mmol, 63%). ^{1}H NMR (400 MHz, $CDCl_3$) δ 2.83 (bs, 1H), 3.27 (t, *J* = 5.2 Hz, 2H), 3.83 (t, *J* = 5.1 Hz, 2H), 6.57 (dd, *J* = 7.6, 1.4 Hz, 1H), 6.68 (dd, *J* = 8.0, 1.4 Hz, 1H), 6.81 (t, *J* = 7.8 Hz, 1H), 7.22–7.30 (m, 3H), 7.38–7.46 (m, 1H). ^{13}C NMR (101 MHz, $CDCl_3$) δ 46.6, 61.4, 111.9, 119.7, 120.7, 126.9, 127.2, 129.0, 129.9, 131.9, 132.4, 134.0, 137.3, 138.1.
* *N-(3-{[2-amino-3-(2-chlorophenyl)phenyl]amino}propyl)benzamide (**72c**)* Using the same procedure described for **72a** and starting from 1-(2-chlorophenyl)-3-fluoro-2-nitrobenzene (114.7 mg, 0.21 mmol, 1 equiv.) and N-(3-aminopropyl)benzamide (89.2 mg, 0.500 mmol, 1.1 equiv.), N-[3-({2'-chloro-2-nitro-[1,1'-biphenyl]-3-yl}amino)propyl]benzamide, **70c** was obtained as an oil (85 mg, 0.21 mmol, 46%). 1H NMR (400 MHz, $CDCl_3$) δ 2.02 - 2.08 (m, 2H), 3.41 (q, *J* = 6.1 Hz, 2H), 3.63 (q, *J* = 6.4 Hz, 2H), 6.35 (bs, 1H), 6.53 (d, *J* = 6.6 Hz, 1H), 6.91 (d, *J* = 8.5 Hz, 1H), 7.00 (t, *J* = 5.2 Hz, 1H), 7.19–7.32 (m, 3H), 7.36–7.53 (m, 5H), 7.76 (d, *J* = 7.2 Hz, 2H).^{13}C DEPT-135 NMR (101 MHz, MeOD) δ 28.8, 37.5, 41.5, 119.0, 119.4, 126.8, 126.9, 128.2, 128.7, 129.5, 131.2, 131.7. Following the reduction step with Sn/HCl described for **70a** and starting from **70c (**78.2 mg, 0.19 mmol, 1 equiv.), the title compound was obtained (21.1 mg, 0.055 mmol, 29%). ^{1}H NMR (400 MHz, CD_3OD) δ 1.97–2.07 (m, 2H), 3.27 (d, *J* = 7.0 Hz, 2H), 3.58 (t, *J* = 7. Hz, 2H), 6.46 (d, *J* = 7.5 Hz, 1H), 6.65–6.86 (m, 2H), 7.29–7.34 (m, 1H), 7.34–7.42 (m, 2H), 7.43–7.49 (m, 2H), 7.53 (d, *J* = 6.8 Hz, 2H), 7.83 (d, *J* = 7.6 Hz, 2H). ^{13}C DEPT-135 NMR (101 MHz, CD_3OD) δ 28.8, 37.5, 41.5, 111.0, 119.0, 119.4, 126.8, 126.9, 128.2, 128.7, 129.5, 131.2, 131.7.
* *3-(2-chlorophenyl)-1-N-(4-phenylbutyl)benzene-1,2-diamine (**72d**)* Using the same procedure described for **72a** and starting from 1-(2-chlorophenyl)-3-fluoro-2-nitrobenzene (150 mg, 0.60 mmol, 1 equiv.) and 4-phenylbutan-1-amine (97.8 mg, 0.66 mmol, 1.1 equiv.), 3-(2-chlorophenyl)-2-nitro-N-(4-phenylbutyl)aniline **70 d** was obtained as an oil (162.8 mg, 0.43 mmol, 72%). 1H NMR (400 MHz, $CDCl_3$) δ 1.67–1.81 (m, 4H), 2.66 (t, *J* = 7.0 Hz, 2H), 3.25 (dt, *J* = 8.1, 5.4 Hz, 2H), 6.46 (dd, *J* = 7.3, 1.3 Hz, 1H), 6.82 (t, *J* = 8.6 Hz, 1H), 6.93 (bt, *J* = 5.2 Hz, 1H), 7.13–7.19 (m, 3H), 7.19–7.30 (m, 5H), 7.31–7.40 (m, 2H). ^{13}C NMR (101 MHz, $CDCl_3$) δ 28.7, 35.5, 43.4, 113.4, 119.0, 125.9, 126.5, 126.8, 128.4, 128.4, 129.3, 129.6, 133.5, 135.5, 136.7, 137.0, 139.1, 141.8, 144.0.
* Following the reduction step with Sn/HCl described for **70a** and starting from **70d** (162.8 mg, 0.43 mmol, 1 equiv), the title compound was obtained (61.7 mg,0.18 mmol, 41 %). 1H NMR (400 MHz, $CDCl_3$) δ 1.60–1.81 (m, 4H), 2.52–2.71 (m, 2H), 3.10 (dt, *J* = 7.0, 1.0 Hz, 3H), 6.52 (dd, *J* = 7.7, 1.4 Hz, 1H), 6.64 (dd, *J* = 7.9, 1.4 Hz, 1H), 6.81 (t, *J* = 7.8 Hz, 1H), 7.08–7.15 (m, 3H), 7.17–7.28 (m, 5H),

7.38–7.47 (m, 1H). ^{13}C NMR (101 MHz, $CDCl_3$) δ 29.1, 29.3, 35.7, 44.3, 111.1, 119.9, 125.8, 126.8, 127.1, 128.3, 128.5, 129.0, 129.8, 132.0, 134.0, 136.0, 138.1, 138.3, 142.2.

* *6-(2-chlorophenyl)-1-N-(2-phenylethyl)benzene-1,2-diamine (***73***)* Using the same procedure described for **72a** and starting from 1-(2-chlorophenyl)-2-fluoro-3-nitrobenzene (30 mg, 0.12 mmol, 1 equiv.) and phenethylamine (15.9 mg, 0.13 mmol, 1.1 equiv.), the title compound was obtained after general reduction procedure with Sn /HCl (6 mg, 0.018 mmol, 16% over 2 steps). ^{1}H NMR (400 MHz, $CDCl_3$) δ 2.64 (t, *J* = 6.8 Hz, 2H), 2.91–3.15 (m, 3H), 3.49 (bs, 2H), 6.54 (dd, *J* = 7.5, 1.5 Hz, 1H), 6.75 (dd, *J* = 7.9, 1.5 Hz, 1H), 6.93 (t, *J* = 7.7 Hz, 1H), 6.98–7.03 (m, 2H), 7.10–7.31 (m, 6H), 7.36–7.42 (m, 1H).

* *4-(2-chlorophenyl)-1-(2-phenylethyl)-1H-1,3-benzodiazol-2-amine trifluoroacetate (***74a***).* Following general method H and starting from **72a** (40 mg, 0.12 mmol, 1 equiv) and BrCN (39.4 mg, 0.37 mmol, 3 equiv.), the title compound **74a** was obtained as a trifluoroacetate salt (20.1 mg, 0.043 mmol, 35%) after purification by C18 reverse phase chromatography. ^{1}H NMR (400 MHz, CD_3OD) δ 3.05 (t, *J* = 6.8 Hz, 2H), 4.35 (t, *J* = 6.8 Hz, 2H), 6.95–7.03 (m, 2H), 7.05–7.13 (m, 4H), 7.23 (d, *J* = 4.4 Hz, 2H), 7.29–7.42 (m, 3H), 7.47–7.53 (m, 1H).^{13}C NMR (101 MHz, $CDCl_3$) δ 37.1, 47.9, 57.3, 113.8, 127.3, 127.9, 129.0, 130.7, 131.2, 132.3, 132.3, 132.6, 133.6, 133.9, 134.3, 135.3, 137.0, 139.0, 140.8, 155.4. HRMS $(M + H)^+$ 348.1276 (calcd for $C_{13}H_9ClN_2SH^+$ 348.1268)

* *2-[2-amino-4-(2-chlorophenyl)-1H-1,3-benzodiazol-1-yl]ethan-1-ol (***74b***)* Following general method H and starting from **72b** (40 mg, 0.15 mmol, 1 equiv) and BrCN (48.4 mg, 0.46 mmol, 3 equiv.), the title compound **74b** was obtained (10.1 mg, 0.035 mmol, 23%) after trituration of the crude with Et_2O. ^{1}H NMR (400 MHz, Methanol-d4) δ 3.79 (t, *J* = 5.2 Hz, 2H), 4.05 (t, *J* = 5.2 Hz, 2H), 6.87 (dd, *J*= 7.6 Hz, J = 1.1 Hz, 1H), 6.87 (t, *J* = 7.7 Hz, 1H), 7.13 (dd, J= 7.8 Hz, 1.1 Hz, 1H), 7.18–7.27 (m, 2H), 7.30 (dt, *J* = 7.8, 3.0 Hz, 1H), 7.39 (dt, *J* = 8.3, 3.0 Hz, 1H). 13C NMR (101 MHz, MeOD) δ 44.7, 60.2, 107.3, 118.8, 122.5, 126.2, 126.6, 128.2, 129.2, 132.0, 133.4, 134.4, 138.3, 139.2, 155.4. HRMS $(M + H)^+$ 288.0916 (calcd $C_{15}H_{14}ClN_3OH^+$ 288,0904)

* *N-{3-[2-amino-4-(2-chlorophenyl)-1H-1,3-benzodiazol-1-yl]propyl}benzamide trifluoro acetate (***74c***).* Following general method H and starting from **72c** (20 mg, 0.052 mmol, 1 equiv) and BrCN (16.7 mg, 0.16 mmol, 3 equiv.), the title compound **74c** was obtained as a trifluoroacetate salt (9.8 mg, 0.019 mmol, 36%) after purification by C18 reverse phase chromatography.^{1}H NMR (500 MHz, $CDCl_3$) δ 2.03 – 2.14 (m, 2H), 3.43 (t, *J* = 6.7 Hz, 2H), 4.19 (t, *J* = 7.9, Hz, 2H), 7.07 – 7.16 (m, 1H), 7.27 – 7.31 (m 2H), 7.32 – 7.39 (m, 4H), 7.41 – 7.46 (m 2H), 7.47 -7.52 (m, 1H), 7.62 – 7.75 (m, 2H). ^{13}C NMR (126 MHz, $CDCl_3$) δ 31.7, 40.9, 44.7, 113.5, 127.4, 128.0, 130.8, 131.1, 131.1, 132.2, 133.7, 133.9, 134.4, 135.4, 135.4, 136.9, 138.0, 138.9, 154.6, 173.1. HRMS $(M+H^+)$ 405.1496 (calcd $C_{23}H_{21}ClN_4OH^+$ 4051482).

* *4-(2-chlorophenyl)-1-(4-phenylbutyl)-1H-1,3-benzodiazol-2-amine trifluoroacetate (***74d***).* Following general method H and starting from **72d** (60 mg, 0.17 mmol, 1 equiv) and BrCN (54.3 mg, 0.5 mmol, 3 equiv.), the title compound **74d** was obtained as a trifluoroacetate salt (55.2 mg, 0.11 mmol, 66%) after purification by C18 reverse phase chromatography. 1H NMR (400 MHz, Methanol-d4) δ 1.77 (quint, *J* = 7.6 Hz, 2H), 1.89 (quint, *J* = 7.3 Hz, 2H), 2.70 (t, *J* = 7.5 Hz, 2H), 4.22 (t, *J* = 7.3 Hz, 2H), 7.13–7.20 (m, 3H), 7.22–7.31 (m, 3H), 7.42 (t, *J* = 7.9 Hz, 1H), 7.39–7.55 (m, 4H), 7.62 (d, *J* = 7.1 Hz, 1H). ^{13}C NMR (101 MHz, MeOD) δ 27.0, 28.0, 34.9, 42.5, 109.7, 123.4, 124.1, 125.2, 125.6, 127.1, 127.2 (2C), 128.0 (2C), 129.7, 130.0, 130.5, 131.4, 133.0, 135.0, 141.5, 150.4. HRMS $(M + H)^+$ 405.1496 (calcd $C_{23}H_{21}ClN_4OH^+$ 405,1482).

* *7-(2-chlorophenyl)-1-(2-phenylethyl)-1H-1,3-benzodiazol-2-amine trifluoroacetate (***75***).* Following general method H and starting from **72d** (6 mg, 0.018 mmol, 1 equiv) and BrCN (6.0 mg, 0.055 mmol, 3 equiv.), the title compound **74d** was obtained as a trifluoroacetate salt (5 mg, 0.014 mmol, 77%) after purification by C18 reverse phase chromatography. HRMS $(M + H)^+$ 348.1283 (calcd for $C_{13}H_9ClN_2SH^+$ 348.1268)

References

1. Gerber, R.; Frech, C.M. Negishi Cross-Coupling Reactions Catalyzed by an Aminophosphine-Based Nickel System: A Reliable and General Applicable Reaction Protocol for the High-Yielding Synthesis of Biaryls. *Chemistry – A European Journal* **2011**, *17*, 11893–11904, doi:10.1002/chem.201101037.
2. Stokes, B.J.; Jovanović, B.; Dong, H.; Richert, K.J.; Riell, R.D.; Driver, T.G. Rh2(II)-Catalyzed Synthesis of Carbazoles from Biaryl Azides. *J. Org. Chem.* **2009**, *74*, 3225–3228, doi:10.1021/jo9002536.

3. Wiles, Jason Allan; Phadke, Avinash S; Deshpande, Milind; Agarwal, Atul; Chen, Dawei; Gadhachanda, Venkat Rao; Hashimoto, Akihiro; Pais, Godwin; Wang, Qiuping; Wang, Xiangzhu; et al. Preparation of aryl, heteroaryl and heterocyclic compounds as factor D inhibitors for treatment of immune and inflammatory disorders. *PCT Int. Appl* 2017, 2017035408.
